# Retinoic acid and FGF signaling interact to control elongation and lineage specification in a mouse gastruloid model

**DOI:** 10.1242/dev.205153

**Published:** 2026-08-13

**Authors:** Mehmet Yildiz, Noëlle Dommann, Tugce Kardelen Hasdemir, Esther de Vries, Anna Alemany, Pascale F. Dijkers, Niels Geijsen

**Affiliations:** ^1^ Department of Anatomy and Embryology, Leiden University Medical Center, 2300RC Leiden, The Netherlands; ^2^ The Novo Nordisk Foundation Center for Stem Cell Medicine (reNEW), Leiden University Medical Center, 2300RC Leiden, The Netherlands

**Keywords:** Gastruloid, Retinoid acid, FGF signaling, Gastrulation, Development, Cell fate decision

## Abstract

Axial elongation and cell fate decisions during early embryonic development are regulated by gradients of signaling molecules, such as retinoic acid (RA) and fibroblast growth factor (FGF). To assess the role of RA *in vitro*, studies have relied on its direct addition to the medium, which can produce teratogenic effects. Here, we examined the role of a RA precursor, retinol, on axial elongation, anteroposterior development, and FGF-dependent signaling in murine gastruloids, an *in vitro* model system for early embryogenesis. Rather than applying RA ectopically, we supplemented a retinoid-free medium with its precursor, retinol, prompting the cells to produce RA endogenously. Our results showed that the spatiotemporal RA and FGF signaling can be manipulated with different doses of retinol, influencing gastruloid elongation and lineage specification. Gastruloids cultured in high doses of retinol showed an enrichment of ectoderm, whereas low doses resulted in early mesoderm specification and increased FGF signaling. We further used our approach to confirm that RA signaling inhibits FGF in gastruloids. Thus, retinol can modulate cellular composition in gastruloids, allowing us to investigate fate commitment of ectoderm and mesoderm progenitors *in vitro*.

## Teaser

**Summary:** Manipulation of retinol concentration in an *in vitro* mouse gastruloid model reveals effects on cellular composition and interactions with fibroblast growth factor signaling.

## INTRODUCTION

In multicellular organisms, events during early embryonic development, such as symmetry breaking, axial elongation and cell fate decisions are tightly coordinated by spatiotemporal patterns of signaling molecules (Duester, 2008; Ghyselinck and Duester, 2019; Morgani and Hadjantonakis, 2020). One example is the two opposing signaling gradients retinoic acid (RA) and fibroblast growth factor (FGF) (Bernheim and Meilhac, 2020; del Corral et al., 2003). FGF8 is expressed in the posterior region of embryonic day (E) 6.0 mouse embryos and is required for gastrulation and neural formation (Crossley and Martin, 1995). RA not only coordinates later trunk formation but also inhibits FGF8 at both the anterior and posterior parts of the embryo, thereby allowing the formation of posterior neural structures, including the neural tube and hindbrain (del Corral et al., 2003; Duester, 2008; Molotkova et al., 2005; Sirbu and Duester, 2006). Many existing studies rely on knockout mice deficient in enzymes that generate RA, in which RA concentrations cannot be fully controlled due to potential maternal contributions (Duester, 2008; Ghyselinck and Duester, 2019). While the teratogenic effects of RA in gastruloids have been described (Mantziou et al., 2021), the physiological effects of RA signaling during mouse gastruloid development have not been systematically studied.

This gap highlights the need for an *in vitro* system in which RA or FGF signaling can be modulated at physiologically relevant concentrations. To address this, we used a retinoid-free medium to culture gastruloids with varying doses of retinol, allowing us to examine changes in morphology, cellular heterogeneity and gene expression in a controlled environment. The precursor of RA, retinol (vitamin A), is a lipophilic molecule that gets taken up by specific cells through a transporter named STRA6 (signaling receptor and transporter of retinol) (Kawaguchi et al., 2007). Retinol is metabolized to retinaldehyde by retinol dehydrogenase (RDH10) (Sandell et al., 2007) and then to RA by retinaldehyde dehydrogenases (ALDH1A1-3) (Mic et al., 2002; Mic et al., 2000; Sirbu and Duester, 2006). The produced RA can act on the same cell or diffuse to neighboring cells, where it interacts with RA receptors (RARs) (Chawla et al., 2001) that subsequently bind to RA-responsive elements (RAREs) (Marshall et al., 1994) in the DNA. Altogether, this results in activation or repression of downstream genes via recruitment of co-regulators (Duester, 2008; Germain et al., 2002). RA is degraded by specific enzymes, such as CYP26, thus inhibiting RA-induced signaling (Abu-Abed et al., 2001; Sakai et al., 2001).

The antagonism between FGF and RA signaling gradients is regulated by cross-regulation of CYP26, FGF and RA-generating enzymes (Bernheim and Meilhac, 2020). In *Aldh1a2^−^*^/−^ embryos, *Fgf8* expression expanded both at the cardiac mesoderm (indicated by the expression of *Isl1*) and at the tailbud, which resulted in smaller and asymmetric somites across the anterior-posterior axis (Molotkova et al., 2005; Sirbu et al., 2008; Vermot et al., 2005). Nevertheless, somite size was rescued with an FGF receptor antagonist (Cunningham et al., 2015), proving that, during somitogenesis, FGF8 is inhibited by RA (Kumar et al., 2016; Kumar and Duester, 2014). Conversely, in *Cyp26^−/−^* embryos, the concentration of intracellular RA increased in the tailbud, resulting in downregulation of *T* and *Wnt3a*. Also, in this mouse model, RA-positive domains in the hindbrain are expanded (Sakai et al., 2001), implying that RA is also important for anterior neural differentiation. These studies all showed that the antagonistic gradients between FGF and RA signaling are crucial for proper axial elongation, somitogenesis, neural formation and heart development.

Here, we used an *in vitro* mouse gastruloid model to study the spatial and temporal physiological effects of RA signaling during early embryonic development (Beccari et al., 2018; van den Brink et al., 2014, 2020). Gastruloids are 3D stem cell models of post-implantation stage embryos that recapitulate crucial embryonic events such as symmetry breaking, axial elongation, and the emergence of several precursor cell types (Beccari et al., 2018; van den Brink et al., 2014, 2020; Veenvliet et al., 2020). Since ectopic RA exposure can induce teratogenic effects (Mantziou et al., 2021), and retinaldehyde bypasses the initial regulatory steps involving retinol dehydrogenase enzymes (Duester, 2008; Sandell et al., 2007), we utilized a retinoid-free medium supplemented with specific concentrations of retinol. This approach ensures that RA signaling is driven by the metabolic capacity of each cell: only cells expressing the required uptake and metabolic genes will convert retinol into functional RA (Duester, 2008; Kawaguchi et al., 2007; Mic et al., 2002; Mic et al., 2000; Sandell et al., 2007; Sirbu and Duester, 2006). Additionally, as commercially available media typically contain retinol, this methodology maintains comparability with standard gastruloid culture conditions while allowing us to study the role of endogenous RA signal activation *in vitro* (Beccari et al., 2018; van den Brink et al., 2020).

Our results demonstrate that gastruloids generate endogenous RA from retinol, subsequently modulating FGF signaling. With high levels of retinol, RA negatively regulated FGF signaling, resulting in inhibition of symmetry breaking and elongation. Furthermore, we demonstrate that cellular composition in gastruloids can be modulated with different retinol concentrations. In the absence of retinol, neuromesodermal progenitors (NMPs) and presomitic mesoderm (PSM) were promoted, while moderate retinol levels promoted the generation of somite and neural progenitors. High retinol levels resulted exclusively in ectoderm-related structures. This establishes a system in which the development of specific cellular progenitors can be directed by simply modulating the retinol dosage.

## RESULTS

### Retinol modulates elongation in mouse gastruloids

**Fig. 1.**
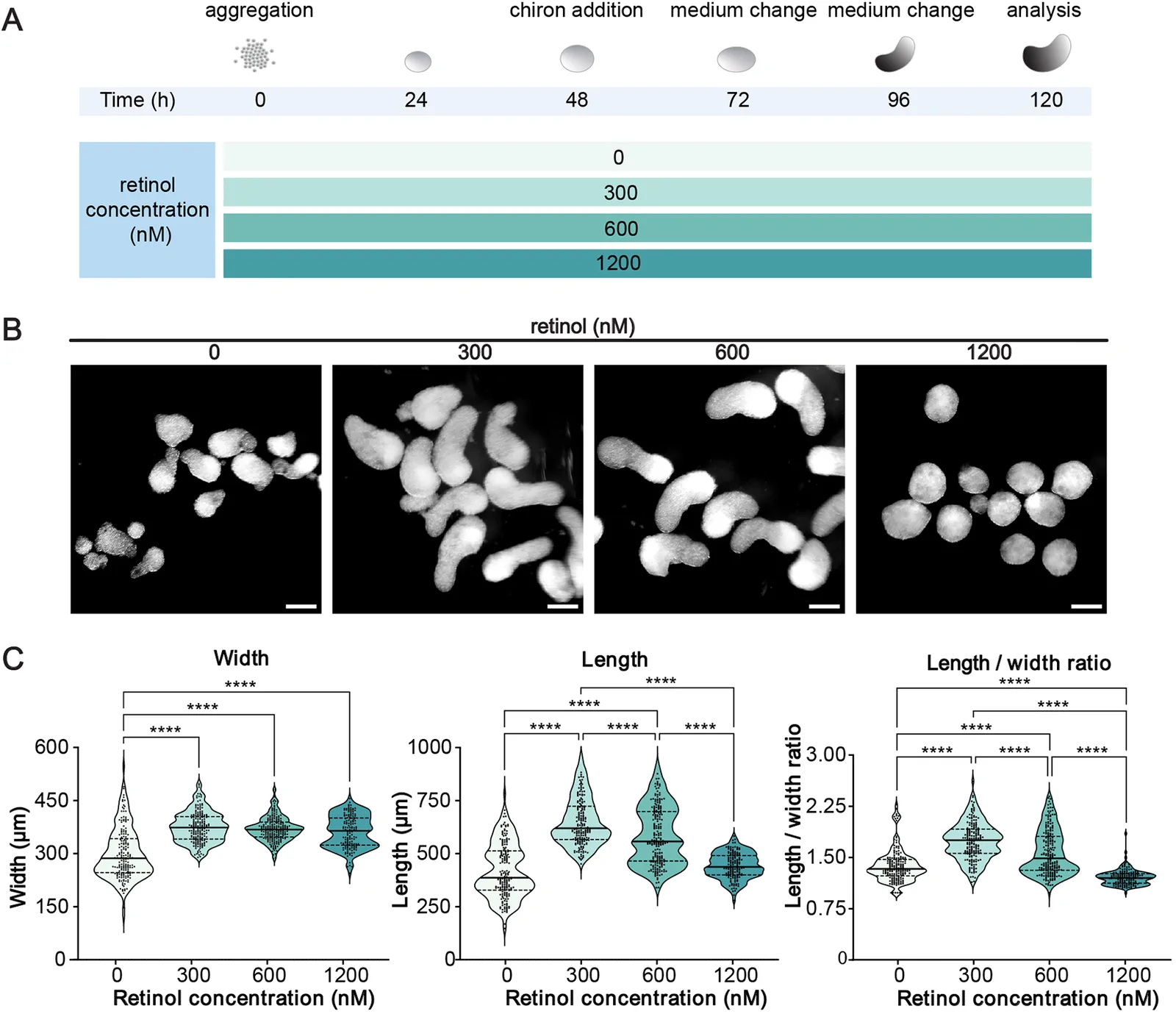
**The effect of exogenous retinol on gastruloid elongation.** (A) Experimental setup for gastruloid generation. (B) Representative images of gastruloids generated from E14 mESCs 120 h after aggregation, and cultured with 0, 300, 600 or 1200 nM retinol. Scale bars: 500 μm. (C) Violin plots showing the distribution of width, length, and length/width ratio of gastruloids 120 h after aggregation treated with 0 nM (*n*=179), 300 nM (*n*=186), 600 nM (*n*=210) or 1200 nM (*n*=150) retinol over *N*=4 biological replicates. Image analysis was performed using the IncuCyte S3 imaging system. Each dot represents a gastruloid; lines in the violin plots show median (solid lines) and quartiles (dashed lines). Experimental groups were compared with one-way ANOVA and *P*-values were corrected with the Tukey test. ****P<0.001.

To examine dose-dependent retinol presence during gastruloid formation, we generated gastruloids (Fig. 1A) from E14 mouse embryonic stem cells (mESCs) using a retinoid-free N2B27 medium with different retinol concentrations (0, 300, 600 and 1200 nM retinol) (van den Brink et al., 2014, 2020). In the absence of retinol, gastruloids displayed little elongation and were small (Fig. 1B,C). Retinol supplementation enhanced elongation, which peaked at 300 nM but decreased at higher concentrations (Fig. 1B,C). Gastruloids became wider with retinol, but this effect did not vary significantly with dose (Fig. 1B,C). Thus, the average length and length/width ratio reached their maximum at a concentration of 300 nM retinol and decreased in a dose-dependent manner, indicating that the excessive retinol adversely affects gastruloid elongation (Fig. 1B,C).

### Retinol treatment suppresses early mesoderm formation and promotes neural differentiation in mouse gastruloids

**Fig. 2.**
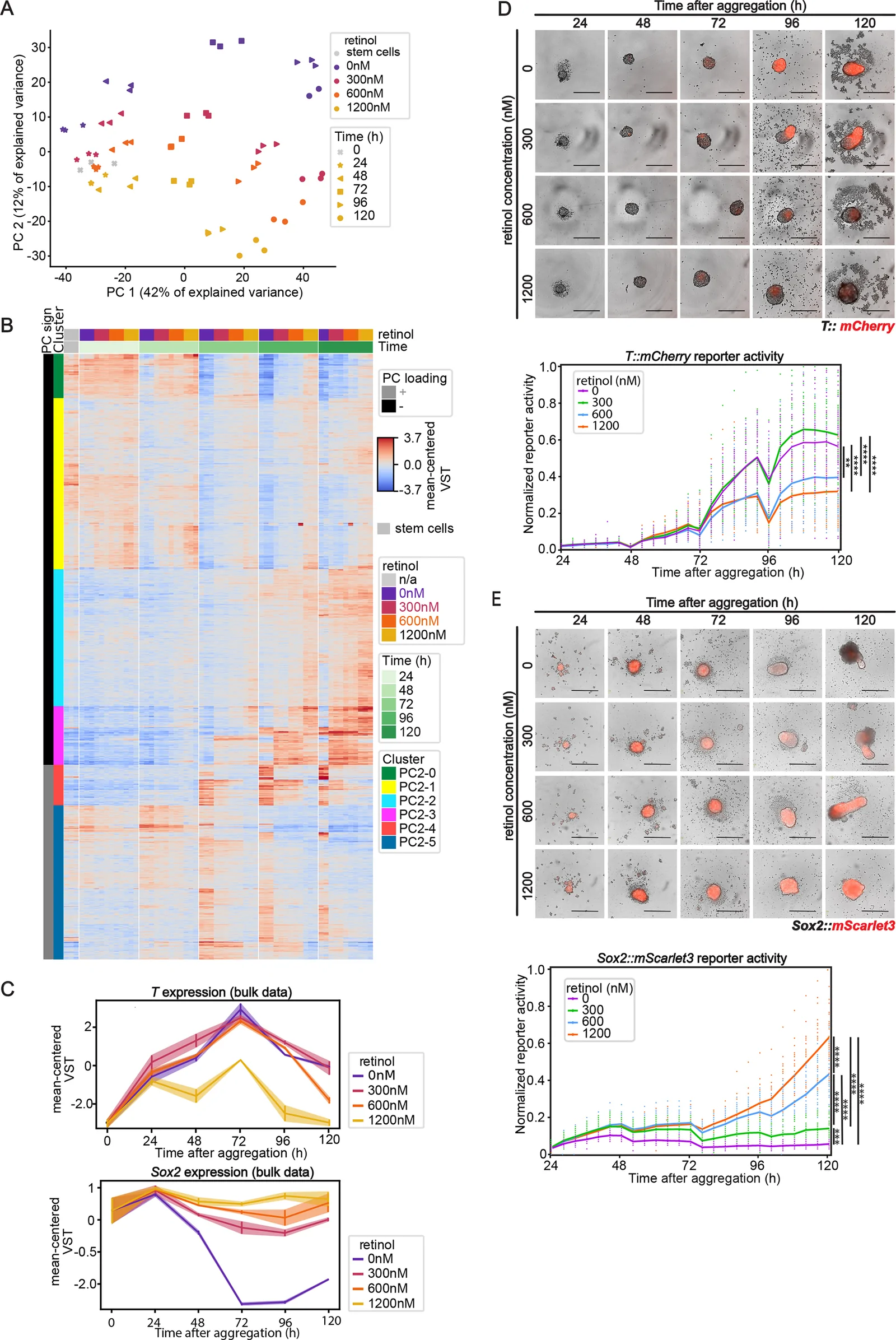
**Time-course bulk RNA-seq and imaging of gastruloids treated with different doses of retinol.** (A) Principal component (PC) analysis of time course RNA-seq data showing PC1 and PC2 and their corresponding explained variance ratio, which captures the effects of time and retinol concentration in gastruloids. Each point represents pooled bulk RNA isolated from mESCs or gastruloids (*n*=40-45; *N*=2 or *N*=3 biological replicates). (B) Heatmap showing the time course mean-centered variance stability transformed (VST) RNA-seq data of genes significantly contributing to PC2 either positively or negatively, clustered according to expression patterns. (C) Line graphs depicting the mean-centered VST levels of *T* and *Sox2* in gastruloids detected from bulk RNA-seq at 0 h (mESCs), or 24 h, 48 h, 72 h and 120 h after aggregation. Solid lines represent the mean of the replicates and the shaded area represents the corresponding s.d. (D) Top: Representative images showing T::mCherry reporter activity (red) in gastruloids treated with 0, 300, 600 or 1200 nM retinol 24 h, 48 h, 72 h, 96 h and 120 h after aggregation. Scale bars: 400 μm. Bottom: Normalized T::mCherry reporter activity. Each dot represents a gastruloid (*n*=38, *n*=37, *n*=38, *n*=41, respectively; *N*=2 biological replicates), and the solid line represents the mean among retinol conditions per time point. (E) Top: Representative images showing Sox2::mScarlet3 reporter activity (red) in gastruloids treated as in D. Scale bars: 400 μm. Bottom: Normalized Sox2::mScarlet3 reporter activity. Each dot represents a gastruloid (*n*=30, *n*=26, *n*=26, *n*=38, respectively; *N*=2 biological replicates), and the solid line represents the mean among retinol conditions per time point. Experimental groups in D and E were compared with mixed-effects analysis with Geisser–Greenhouse correction. Statistics are shown only for the last time point. **P<0.01, ***P<0.001, ****P<0.0001.

To investigate the effect of retinol on dynamic gene expression patterns during gastruloid development, we performed time-course bulk RNA-sequencing (RNA-seq) during gastruloid formation with different concentrations of retinol. Differential gene expression analysis was performed for each sample, characterized by time point and retinol concentration, relative to the stem cells. The results revealed a significant increase in the number of differentially expressed genes with increasing time points, with a similar set of genes being detected in all samples (Fig. S1). Principal component analysis (PCA) was performed as an unsupervised approach to identify the main sources of variation in the dataset. PC1 primarily captured temporal variation, whereas PC2 accounted for differences in retinol concentration, with this effect becoming most evident at later time points (Fig. 2A,B, Fig. S1A).

We identified genes with significant loadings on PC1 (both positive and negative) and performed hierarchical clustering based on their expression patterns, which separated them into two groups (clusters PC1-0 and PC1-1) (Fig. S1A, Table S1). We performed Metascape analysis to identify biological processes that were significantly enriched in each gene set. Results indicated that cluster PC1-0, containing genes for which the expression pattern decreased with time regardless of retinol concentration, is related to pluripotency (Fig. S1B, Table S1). Conversely, cluster PC1-1, consisting of genes for which expression increased with time, is associated with crucial events occurring during early development, such as anterior-posterior pattern specification, neural tube formation and tissue morphogenesis as well as mesenchyme development (Fig. S1B, Table S1).

Next, we identified genes with significant loadings on PC2 upon hierarchical clustering. They were classified into six clusters (clusters PC2-0 to PC2-5) based on their expression patterns (Fig. 2B, Table S1). Metascape analysis revealed that cluster PC2-0 was enriched with genes associated with pluripotency (Fig. S1C, Table S1); cluster PC2-1 contained genes associated with primordial germ cell differentiation as well as early neural differentiation; clusters PC2-2 and PC2-3 contained genes associated with neural development and differentiation; and clusters PC2-4 and PC2-5 contained genes associated with mesoderm, skeletal system as well as heart development. Gene expression levels of clusters PC2-0 and PC2-1 were highest at earlier time points (between 24 and 48 h after aggregation), whereas the expression levels of clusters PC2-2-4 were higher at later time points (between 72 and 120 h after aggregation). Hence, we observed that at later time points higher retinol concentrations resulted in increased expression levels of neural development genes (clusters PC2-2 and PC2-3), and decreased expression of genes associated with mesodermal structures (clusters PC2-4 and PC2-5), showing an inverse relationship between these two gene sets (Fig. S1D).

While PCA showed that retinol concentrations drive sample segregation as early as 24 h (Fig. 2A), we investigated whether the underlying gene signatures remained constant throughout the time course. Differential expression analysis (DESeq2) against the stem cell baseline (0 h) revealed that the drivers of early divergence (24 h) are distinct from those at 120 h (Fig. S2A, Table S2), with a significantly larger and non-overlapping gene set emerging at the later time point (Fig. S2B-D, Table S2).

Next, we decided to zoom into the expression patterns of two key markers of mesodermal and ectodermal differentiation: *T* (a mesodermal differentiation marker present in cluster PC2-5) and *Sox2* (pluripotency and an early ectoderm marker present in cluster PC2-0) (Thomson et al., 2011; Veenvliet et al., 2020). With increasing concentrations of retinol, expression of *T* was downregulated, whereas expression of *Sox2* was upregulated (Fig. 2C). Given the observed effect of retinol on the mesodermal and ectodermal commitment during gastruloid development, we set out to validate their expression dynamics using a T (*T::mCherry*) reporter cell line (Deluz et al., 2016) and a Sox2 (*Sox2::mScarlet3*) reporter cell line. We cultured gastruloids and quantified reporter activity by time-lapse imaging (Incucyte S3, 24-120 h post-aggregation) and measured median fluorescence intensity of T::mCherry- and Sox2::mScarlet3-positive cells by fluorescence-activated cell sorting (FACS) at 120 h. T::mCherry expression was induced around 72 h, independently of retinol dose, peaked at 300 nM, and was reduced at higher concentrations (600 and 1200 nM) at later time points (Fig. 2D, Table S3). This trend was confirmed by FACS, in which median T::mCherry intensity was highest at 300 nM and decreased with increasing retinol concentrations (Fig. S3A). In contrast, Sox2::mScarlet3 reporter activity and median fluorescence intensity increased with increasing retinol doses in a dose- and time-dependent manner (Fig. 2E, Table S3, Fig. S3B).

Together with the bulk RNA-seq data, these time-lapse imaging and FACS results demonstrate that retinol promotes ectoderm formation while suppressing mesodermal fates in gastruloids.

### RA modulates cellular composition in mouse gastruloids

**Fig. 3.**
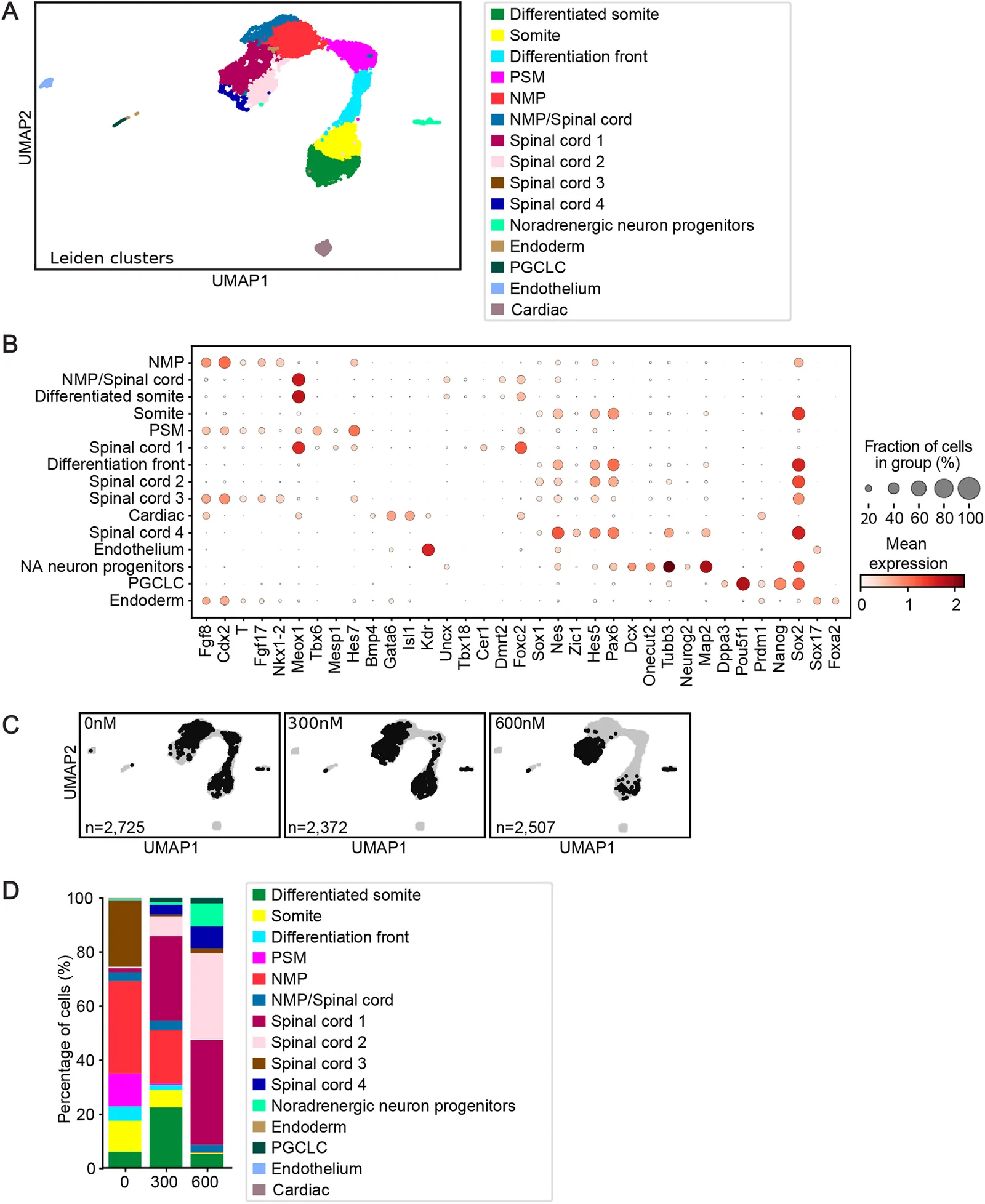
**The effect of retinol on cell heterogeneity.** (A) UMAP representing cells from 120 h *Rarb2-lacZ* gastruloids, treated with different concentrations of retinol (*N*=1 replicate, *n*=80-90 gastruloids were used per condition) and the cell types associated with the Leiden clusters. (B) Dot plot showing both the proportion of cells expressing selected genes and their average expression levels across different cell groups. (C) UMAPs showing cells from 120 h gastruloids, where cells belonging to each retinol concentration are colored in black, with the rest colored in gray. *n*=2725 (0 nM retinol), *n*=2372 (300 nM retinol) and *n*=2507 (600 nM retinol). (D) Bar graph representing the percentages of different cell types within 120 h gastruloids for different concentrations of retinol.

To investigate how retinol affects cellular identity, we performed single-cell RNA sequencing (scRNA-seq) on gastruloids 120 h after aggregation. Gastruloids were generated from the *Rarb2*-*lacZ* reporter line (Shen et al., 1992) with different retinol concentrations; however, *lacZ* reporter expression was not detected in the sequencing data. Experimental samples were integrated with previously published gastruloid scRNA-seq datasets (van den Brink et al., 2020) and jointly visualized in uniform manifold approximation and projection (UMAP) space, with cell-type annotations transferred where possible from the reference dataset (Fig. S4A,B, Tables S4, S5). This demonstrates that our dataset is transcriptionally consistent with the previously established reference gastruloid scRNA-seq dataset. For retinol-dependent populations not represented in the reference atlas, we performed manual annotation using established tissue markers, integration with the mouse embryo atlas (Pijuan-Sala et al., 2019), and GO analysis (Fig. S4C, Tables S6, S7). We confirmed cell-type annotations using selected marker genes (Fig. 3B). This yielded the following cellular identities: ‘differentiated somite’, ‘somite’, ‘differentiation front’, ‘PSM’, ‘NMP’, ‘NMP/spinal cord’, ‘spinal cord 1-4’, ‘noradrenergic neuron progenitors’, ‘endoderm’, ‘PGCLC’, ‘endothelium’ and ‘cardiac’ (Fig. 3A, Fig. S4A,C, Tables S6, S7). Notably, cardiac cells were absent, and endothelial cells were under-represented across all conditions, consistent with known variability in gastruloid differentiation between studies, likely reflecting differences in cell line bias and experimental processing.

Localization of relevant genes with expression in ectoderm and mesoderm (Fig. 3A,B) provided further insight into the ‘spinal cord 1-4’ clusters. Clusters in ‘spinal cord 1’ displayed predominantly mesodermal gene expression (i.e. *Meox1*, *Tbx6*, *Foxc2*), whereas ‘spinal cord 3’ showed NMP (i.e. *Cdx2*, *T*, *Fgf8*) and neural expression (i.e. *Sox1*, *Nes*, *Pax6*). In contrast, ‘spinal cord 2’ and ‘spinal cord 4’ appeared more ectodermal (i.e. *Sox1*, *Nes*, *Pax6*) (Fig. 3B).

Next, we aimed to examine closely the effect of retinol on spinal cord populations. We performed differential gene expression analysis across retinol concentrations, focusing on the spinal cord 1-4 populations, which we combined into a single cell type for this analysis. Consistently, differential gene expression and Gene Ontology (GO) analysis indicated that high retinol promotes neural maturation. At 600 nM retinol, there was significant enrichment of the ‘nervous system development’ pathway (GO:0007399), with upregulation of key regulators, including *Pax3*, *Neurog1* and *Pax7* (Fig. S4D).

To assess retinol-dependent shifts in cell composition, we stratified the UMAP by treatment condition (Fig. 3C,D). Differential abundance analysis revealed a dose-dependent bias in lineage output (Fig. 3C,D): mesodermal populations, including NMPs and PSM and spinal cord 3 were enriched in the absence of retinol, while intermediate mesodermal derivatives (somite and differentiated somite) were most abundant at 0-300 nM. In contrast, high retinol (600 nM) increased the proportion of ectoderm-derived populations, including spinal cord clusters 2-4 and neural progenitors.

Together, these data demonstrate that retinol acts as a key regulator of cell fate decisions in gastruloids, exerting opposing effects on mesodermal and ectodermal lineages. In the absence of retinol, gastruloids predominantly adopt NMPs and mesodermal identities, including PSM and somite, whereas retinol exposure promotes the ectodermal program, favoring the emergence of neural progenitors.

### Retinol modulates mesoderm-ectoderm specification and mesoderm patterning in gastruloids

To validate our scRNA-seq findings, we analyzed reporter lines for ectoderm and mesoderm to quantify the effect of retinol on the relative contribution of early mesodermal (T::mCherry) and ectodermal (Sox2::Venus and Sox1::GFP) populations. Gastruloids from two independent double-reporter embryonic stem cell lines, *T::mCherry;Sox2::Venus* (Veenvliet et al., 2020) and *T::mCherry;Sox1::GFP* (Deluz et al., 2016), were analyzed by FACS at 120 h after aggregation to determine the proportion of reporter-positive populations under increasing retinol concentrations.

**Fig. 4.**
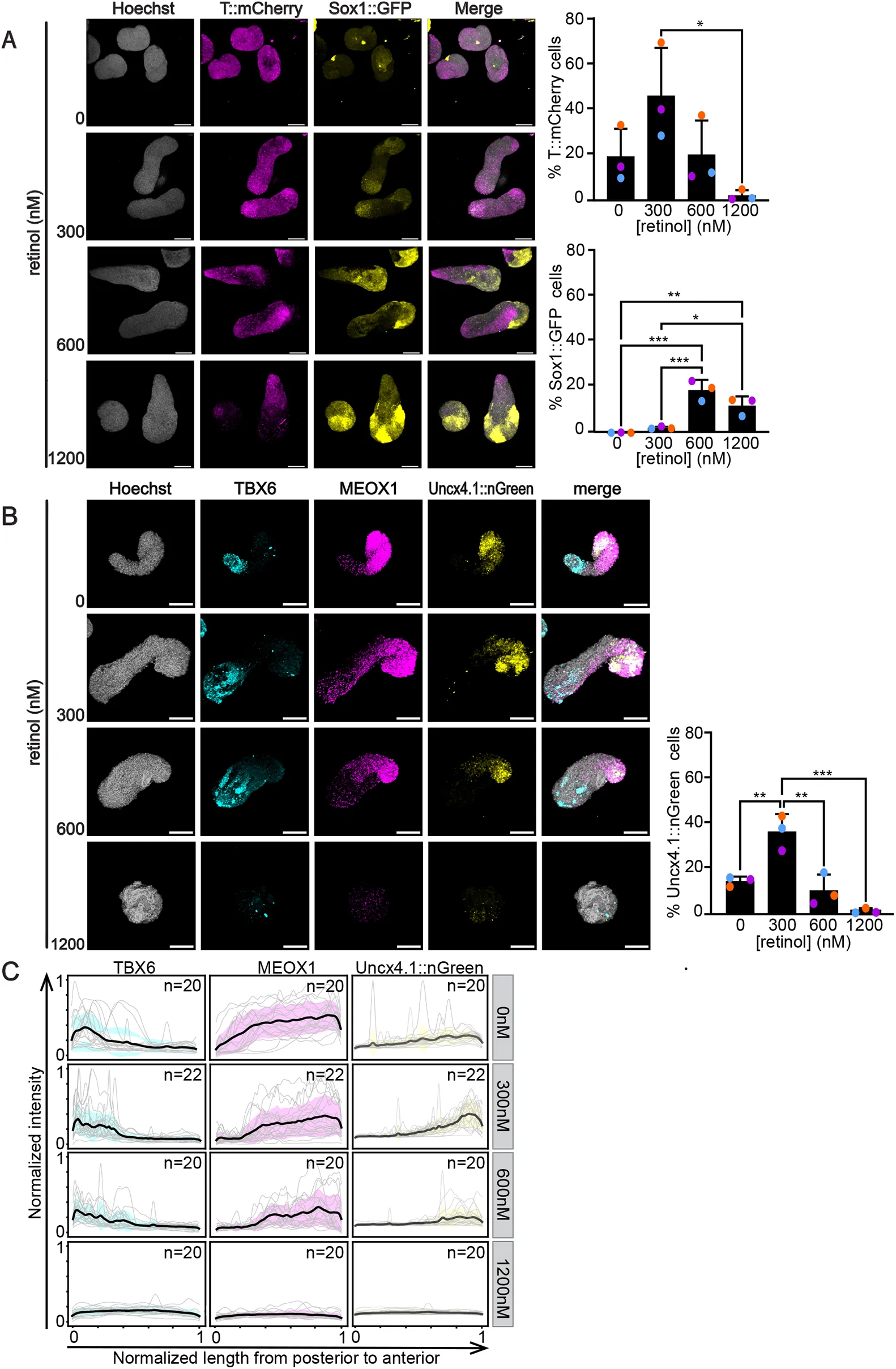
**The effect of retinol on mesoderm-ectoderm specification and mesoderm patterning in gastruloids.** (A) Left: Representative images of 120 h gastruloids expressing T::mCherry (magenta) and Sox1::GFP (yellow), treated with different concentrations of retinol, co-stained with Hoechst (gray). Scale bars: 200 μm. Right: Percentages of mCherry (*T::mCherry* reporter)- and GFP (Sox1::GFP)-positive cells in 120 h gastruloids, measured by FACS. Between 40 and 45 gastruloids were used per experimental group (*N*=3). Each colored dot represents a biological replicate. Graphs show mean±s.d. Experimental groups were compared using one-way ANOVA, with *P*-values adjusted by Tukey's test (*P<0.05, **P<0.01, ***P<0.001). (B) Left: Representative images of gastruloids treated as in A stained with TBX6 (cyan) or MEOX1 (magenta) antibodies, or with an *Uncx4.1::NeonGreen* reporter (Uncx4.1::nGreen; yellow). Gray: Hoechst. Scale bars: 200 μm. Right: Percentages of Uncx4.1::NeonGreen-positive cells in 120 h gastruloids measured by FACS. Between 40 and 45 gastruloids were used per experimental group (*N*=3). Each colored dot represents a biological replicate. Graphs show mean±s.d. Experimental groups were compared using one-way ANOVA, with *P*-values adjusted by Tukey's test (**P<0.01, ***P<0.001). (C) Relative intensity profiles of TBX6 (cyan), MEOX1 (magenta) and Uncx4::NeonGreen (yellow) measured from posterior to anterior in the gastruloids shown in B. Each gray line represents one gastruloid; shaded areas indicate s.d., and black lines indicate the mean. *n*=20 (0 nM retinol), *n*=22 (300 nM retinol), *n*=20 (600 nM retinol), *n*=20 (1200 nM retinol); *N*=2 independent biological experiments.

In agreement with both our time-lapse imaging (Fig. 2D,E) and scRNA-seq analysis (Fig. 3), increasing retinol concentrations resulted in a progressive decrease in the fraction of T::mCherry-positive cells (Fig. 4A, Fig. S5A,B), indicative of reduced mesodermal specification, and a concomitant increase in cells positive for Sox2::Venus (Fig. S5A,B) or Sox1::GFP (Fig. 4A), consistent with enhanced ectodermal identity. This confirms our RNA-seq data, showing a dose-dependent increase in ectodermal cells and a corresponding decrease in mesodermal cells with increasing retinol concentrations.

Given that mesoderm maturation proceeds sequentially from mesoderm to PSM and somite stages in both embryos and gastruloids, we next examined spatial patterning, using staining for TBX6 (mesoderm), MEOX1 (PSM) and *Uncx4.1::NeonGreen* reporter (somite) (Tani et al., 2020; van den Brink et al., 2020; Veenvliet et al., 2020). This allowed us to assess how retinol influences mesoderm organization along the anterior-posterior axis.

At lower retinol concentrations, TBX6 expression was enriched in the posterior region of gastruloids and gradually decreased anteriorly (Fig. 4B,C). At 1200 nM retinol, expression dramatically decreased. MEOX1 staining increased from posterior to anterior across conditions and similarly decreased with higher retinol concentrations, indicating a general suppression of mesodermal gene expression (Fig. 4B,C). A reporter line for Uncx4.1 (*Uncx4.1::NeonGreen*) showed highest expression in more differentiated somite-like regions at 300 nM retinol (Fig. 4B,C). We quantified this *Uncx4.1* driven NeonGreen expression with FACS analysis (Fig. 4B, right). In line with staining, the percentage of NeonGreen-positive cells was maximal at 300 nM retinol and decreased with higher retinol concentrations. At 1200 nM retinol, expression of all three mesodermal markers was dramatically decreased and displayed no clear anterior-posterior patterning (Fig. 4B,C).

Together, these data demonstrate that retinol promotes ectodermal identity while suppressing early mesodermal specification. At intermediate concentrations, it supports somite differentiation, whereas at higher concentrations it disrupts mesoderm formation and abolishes axial patterning.

### Gastruloids metabolize intracellular RA and recapitulate anterior-posterior RA/FGF signaling polarity

Mouse embryos exhibit opposing RA and FGF signaling gradients that direct development (Cunningham et al., 2013; del Corral et al., 2003; Duester, 2008). From E6.5 to E8, *Fgf8* and *Fgf17* are first transcribed at the posterior primitive streak and then in the heart field (Maruoka et al., 1998). When RA is generated at the trunk, *Fgf8* expression is downregulated both at the anterior heart field and at the posterior part of the embryo (i.e. in the tailbud), resulting in neural differentiation (del Corral et al., 2003; Sirbu et al., 2008; Zhao and Duester, 2009). In gastruloids, it has previously been shown that FGF signaling promotes mesoderm differentiation, and the ectopic RA disrupts elongation and promotes neural differentiation (Mantziou et al., 2021; Underhill and Toettcher, 2023).

**Fig. 5.**
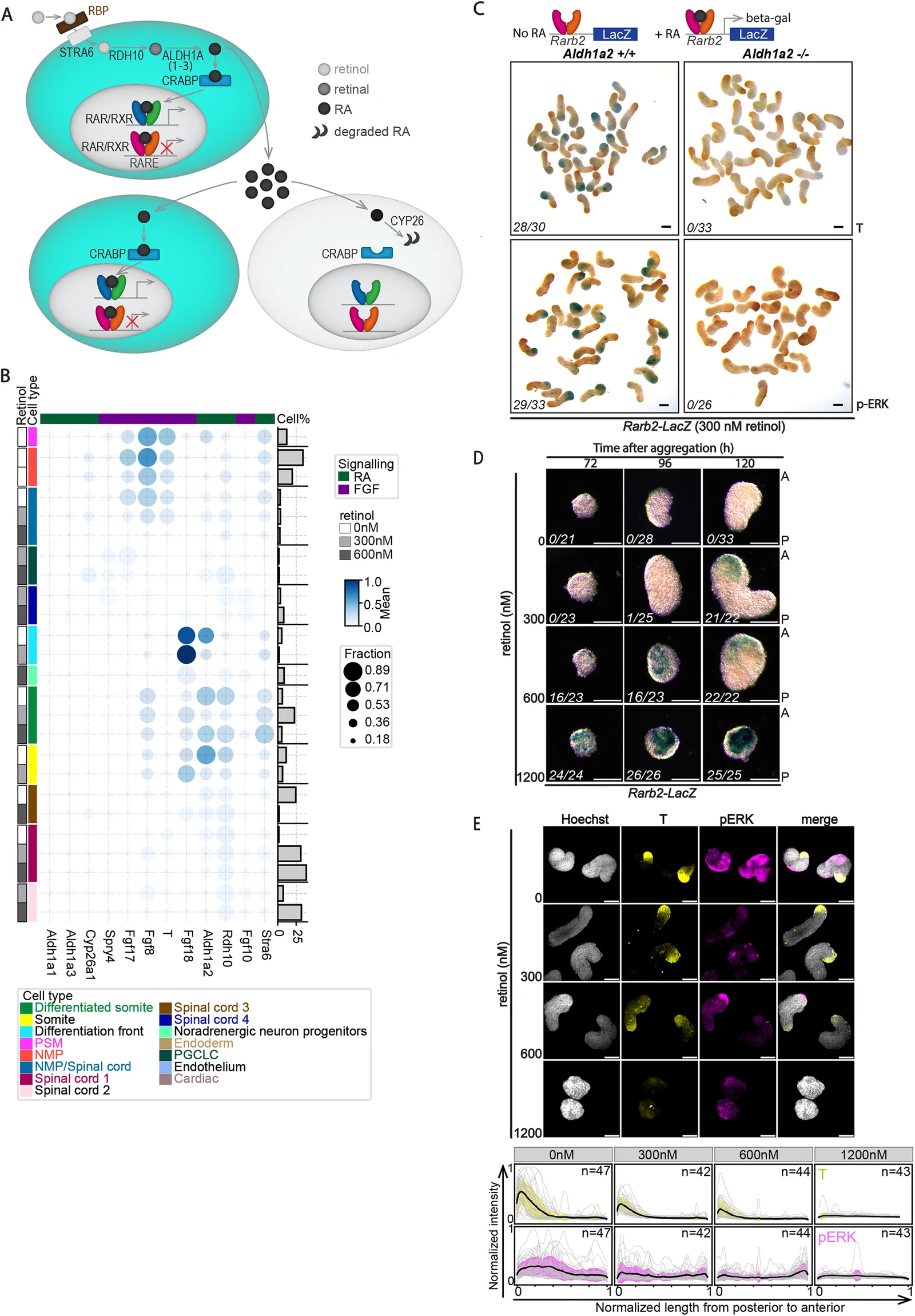
**Opposing RA and FGF signaling localization in gastruloids.** (A) Scheme describing the generation and fate of RA. Retinol is taken up by the cell through the transporter STRA6, and is subsequently metabolized to retinaldehyde and RA by RDH10 and retinal dehydrogenase (ALDH1A1-3), respectively. RA can translocate to the nucleus and modulate transcription by binding to RAR/RXR receptors. RA can also be secreted and diffuse to neighboring cells, where it can also modulate transcription, or, alternatively, be degraded by Cyp26a1 (CYP26). (B) Dot plot showing different cell types in gastruloids associated with different retinol concentrations (left), their expression levels of RA signaling-related genes (*Stra6*, *Rdh10*, *Aldh1a1*, *Aldh1a2*, *Aldh1a3*, *Cyp26a1*) and FGF signaling-related genes *(Fgf8*, *Fgf10*, *Fgf17*, *Fgf18*, *Spry4*), as well as the fraction of each cell type (right). (C) Top: Schematics representing the *Rarb2-lacZ* reporter mESC cell line, expressing β-gal under control of the *Rarb2* promoter. Below: Comparison of *Rarb2-lacZ* reporter activity (X-gal staining, blue) in the presence (*+/+*) or absence (*−/−*) of *Aldh1a2* stained together with either T (HRP staining, brown; top) or pERK (HRP staining, brown; bottom). Scale bars: 200 μm. (D) The effect of different retinol concentrations on *Rarb2-lacZ* reporter activity at 72 h, 96 h and 120 h after aggregation. RA-driven β-gal expression is shown by X-gal staining (blue). Scale bars: 200 μm. The numbers in the images show the X-gal stained versus total number of gastruloids. (E) Top: Gastruloids treated as in Fig. 1A were stained with T (yellow) and pERK (magenta). Gray: Hoechst. Scale bars: 200 μm. Bottom: Relative intensity of T (yellow) and pERK (magenta) staining measured along the posterior-to-anterior axis in gastruloids. The gray lines show individual gastruloids, the black line shows the mean intensity across all replicates (indicated in each panel), and the colored area indicates the s.e.m. *n*=47 (0 nM retinol), *n*=42 (300 nM retinol), *n*=44 (600 nM retinol), *n*=43 (1200 nM retinol); *N*=3 independent biological experiments.

We examined crosstalk and dynamics between RA and FGF, studying genes related to RA metabolism (Fig. 5A) and FGF signaling in our scRNA-seq dataset to identify which cell types express genes involved in retinol uptake (*Stra6*) (Kawaguchi et al., 2007), subsequent RA synthesis (*Rdh10*, *Aldh1a1-3)* (Fig. 5A) (Mic et al., 2002; Mic et al., 2000; Sandell et al., 2007; Sirbu and Duester, 2006), RA degradation (CYP26; e.g. *Cyp26a1*) (Fig. 5A) (Abu-Abed et al., 2001; Sakai et al., 2001), and involvement in FGF signaling (*Fgf8*, *Fgf10*, *Fgf17*, *Fgf18* and *Spry4*) (Kelly et al., 2001; Maruoka et al., 1998; Morgani et al., 2018). scRNA-seq revealed that FGF pathway-associated genes, including *Fgf8*, *Fgf17* and *Spry4*, as well as *Cyp26a1* (encoding the RA-degrading enzyme CYP26), were predominantly expressed in posterior cell populations such as NMPs, PSM and NMP/spinal cord-like cells (Fig. 5B). Notably, these populations were enriched under low retinol conditions (Fig. 5B). In contrast, *Fgf18* expression was detected in cell populations of the differentiation front, somites and differentiated somites, where it was co-expressed with RA signaling-associated genes, including *Stra6*, *Rdh10* and *Aldh1a2* (Fig. 5B). Interestingly, in these cell clusters, the expression level of *Aldh1a2* was highest at 0 nM retinol compared to higher retinol concentrations, which could be due to a negative-feedback loop at higher retinol concentrations (Fig. 5B) (Dobbs-McAuliffe et al., 2004; Feng et al., 2010; Niederreither et al., 1997). Together, these patterns suggest a spatial segregation of signaling activities, with higher FGF signaling in posterior regions and elevated RA signaling in anterior regions of gastruloids.

Since scRNA-seq lacks spatial resolution, we next sought to validate these observations by staining. To investigate further the spatiotemporal dynamics of RA signaling in response to retinol treatment, we employed mESCs expressing a *Rarb2-lacZ* reporter, driving β-galactosidase expression under control of the RA-dependent *Rarb2* promoter (Shen et al., 1992) (Fig. 5C). To confirm that *lacZ* activity depends on the metabolic conversion of retinol into RA, we generated a knockout of *Aldh1a2*, an enzyme that is crucial for the conversion of retinol to RA, in the *Rarb2-lacZ* background (see Materials and Methods) (Fig. S6A) (Ghyselinck and Duester, 2019; Niederreither et al., 1999; Shen et al., 1992). Gastruloids derived from wild-type and *Aldh1a2^−/−^* cells were cultured in the presence of 300 nM retinol and analyzed at 120 h post-aggregation (Fig. 5C). While wild-type gastruloids exhibited robust β-galactosidase expression (57/63), *Aldh1a2^−/−^* gastruloids showed a complete absence of staining (0/59) (Fig. 5C), as previously shown *in vivo* (Niederreither et al., 1999), while treatment of *Rarb2*-*lacZ Aldh1a2^−/−^* cells with RA induced robust staining (Fig. S6B). These results demonstrate that *lacZ* reporter activity is strictly dependent on endogenous RA synthesis from retinol.

To visualize a spatial relationship between RA and FGF signaling in gastruloids, we analyzed *Rarb2-lacZ* reporter activity (Shen et al., 1992) together with staining for either T (brachyury) as a posterior marker or phosphorylated ERK (pERK) as a downstream readout of FGF signaling (Underhill and Toettcher, 2023) (Fig. 5C). In *Aldh1a2^+/+^* gastruloids, β-galactosidase expression was confined to anterior regions, demonstrating RA-dependent signaling. However, both T and pERK staining were restricted to the posterior domain (Fig. 5C). These observations support the existence of robust polarization of RA and FGF signaling in gastruloids.

We next assessed dose-dependent effects of retinol on RA signaling dynamics; *Rarb2-lacZ* gastruloids were cultured with increasing concentrations of retinol and analyzed at different time points after aggregation (Fig. 5D; Shen et al., 1992). Without retinol, no β-galactosidase activity was detected, in agreement with lack of induction in the *Rarb2-lacZ Aldh1a2^−/−^* gastruloids shown in Fig. 5C (Fig. 5D). In contrast, retinol-treated gastruloids exhibited dose-dependent staining for β-galactosidase activity at 72, 96 and 120 h post-aggregation (Fig. 5D). At 300 nM, the staining was restricted to the anterior region and present only at 120 h, whereas with higher retinol concentrations it was detectable as early as 72 h and more pronounced, indicating increased RA signaling (Fig. 5D).

Finally, we examined how retinol influences posterior identity and FGF signaling by analyzing T and pERK expression at 120 h (Fig. 5E). We also quantified the anterior-posterior localization of T and pERK expression (Fig. 5E, bottom). At 0 nM, both T and pERK activity were the highest at posterior part of gastruloids (Fig. 5E). Increasing retinol concentrations progressively reduced both posterior T and pERK expression (Fig. 5E). Posterior localization of these markers was lost entirely at 1200 nM retinol (Fig. 5E). These data reveal that retinol downregulates both posterior T expression and FGF signaling, coinciding with a failure of gastruloid elongation.

Taken together, our results demonstrate that distinct cell populations in gastruloids locally produce RA, giving rise to a polarized anterior-posterior RA/FGF signaling axis. With no retinol or lower retinol concentrations, FGF signaling predominates posteriorly, supporting axial elongation, whereas higher retinol concentrations increase RA signaling, disrupt posterior FGF activity, and impair elongation. These findings suggest that RA acts as a modulator of FGF signaling, and the balance between these pathways is crucial for proper anterior-posterior patterning in gastruloids.

### RA inhibits FGF signaling, which is necessary for early mesoderm formation and elongation in gastruloids

Elongated gastruloids exhibited opposing FGF and RA gradients that were disturbed with increasing retinol concentrations (Fig. 5D,E). Since increased RA signaling correlated with loss of FGF spatial patterning and decreased FGF-dependent target gene expression (Fig. 5D,E), we hypothesized that excessive RA suppresses FGF signaling, mesoderm formation and gastruloid elongation, while promoting ectodermal differentiation.

**Fig. 6.**
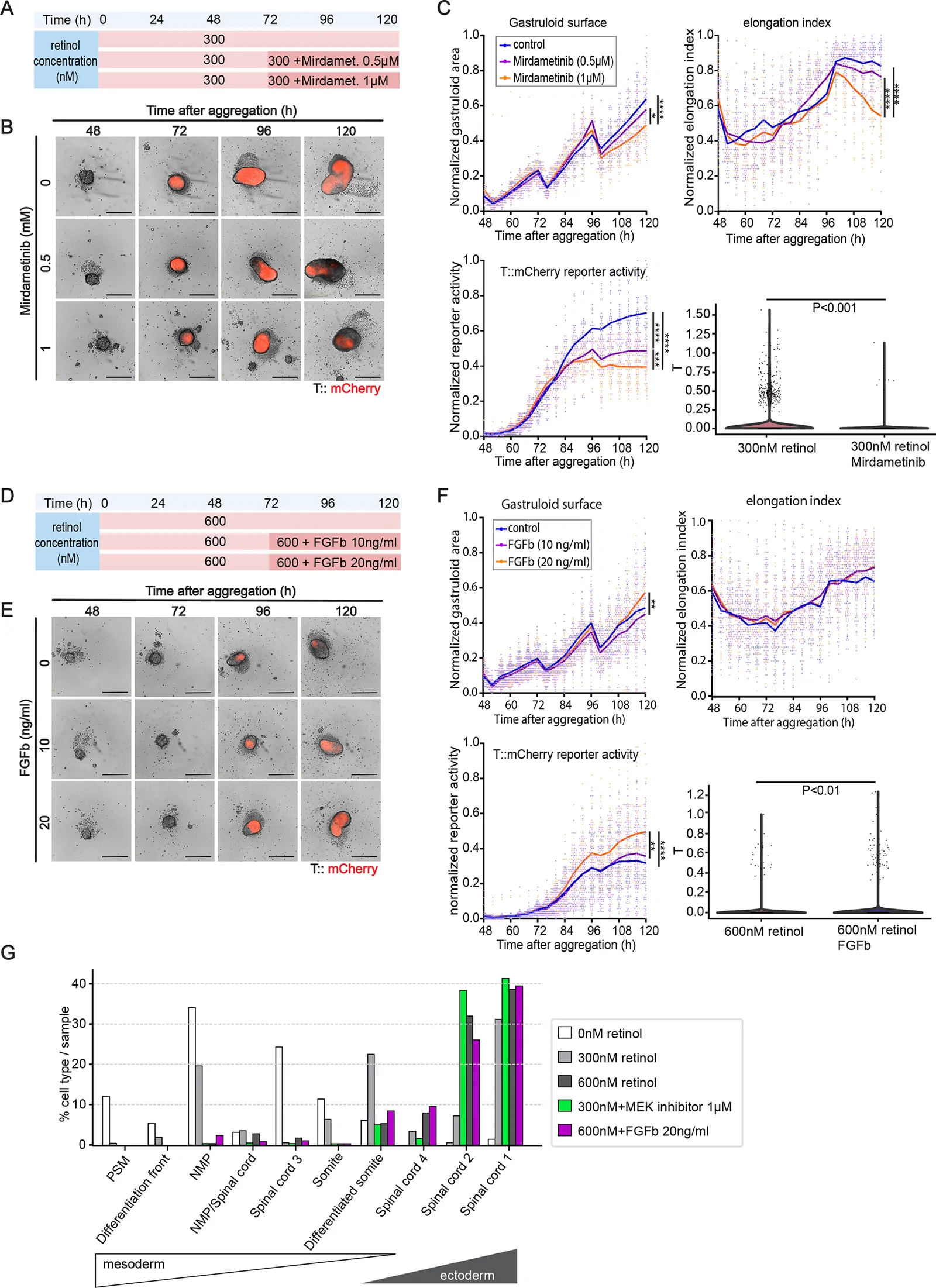
**Effect of FGF signaling on gastruloid elongation, T::mCherry reporter activity and cell heterogeneity.** (A) Experimental design to examine the effect of the MEK inhibitor mirdametinib on gastruloid elongation. (B) Representative images of T::mCherry gastruloids treated as shown in A. Red: T::mCherry. Scale bars: 400 μm. (C) Graphs depicting normalized gastruloid area, normalized elongation index and normalized T::mCherry reporter activity. *n*=35 (control), *n*=38 (0.5 μM mirdametinib) and *n*=45 (1 μM mirdametinib); *N*=2 biological replicates. Each dot represents a gastruloid. Solid lines show the mean. Experimental groups were compared with mixed-effects analysis with Geisser–Greenhouse correction. Statistics are shown only for the last time point. *P<0.05, **P<0.01, ***P<0.001, ****P<0.0001. Bottom right: Violin plot showing the distribution of *T* expression in all cells within gastruloids treated with mirdametinib (1 μM) and its corresponding control (300 nM retinol and DMSO). *P*-value is the Benjamini–Hochberg corrected *P*-value resulting from differential gene expression analysis between samples by the Wilcoxon rank-sum test. *P*<0.001. (D) Experimental design to test the effect of FGFb on gastruloid elongation and T::mCherry reporter activity with 600 nM retinol. (E) Representative images of T::mCherry gastruloids treated as in D. Red: T::mCherry. Scale bars: 400 μm. (F) Graphs showing normalized gastruloid area, normalized elongation index and normalized T::mCherry reporter activity. *n*=58 (control), *n*=61 (20 ng/ml FGFb), *n*=48 (40 ng/ml FGFb); *N*=2 biological replicates. Each dot represents a gastruloid. Solid lines show the mean. Experimental groups were compared with mixed-effects analysis with Geisser–Greenhouse correction. Statistics are shown only for the last time point. **P<0.01, ****P<0.0001. Bottom right: Violin plot showing the distribution of *T* expression in all cells within gastruloids treated with 600 nM retinol with or without FGFb (20 ng/ml). *P*-value is the Benjamini–Hochberg corrected *P*-value resulting from differential gene expression analysis between samples, computed applying the Wilcoxon rank-sum test. *P*<0.01. (G) Bar graph showing the percentage of each cell types in gastruloids treated with various doses of retinol, MEK inhibitor (mirdametinib) or FGFb.

To test our hypothesis, we first cultured gastruloids with 300 nM retinol to promote optimal elongation and treated them during the elongation phase (72-120 h after aggregation) with a MEK inhibitor, mirdametinib (Barrett et al., 2008) (Fig. 6A, Fig. S7A), inhibiting MEK downstream of FGF, or with an FGF receptor inhibitor, infigratinib (Guagnano et al., 2011) (Fig. S8A) using a *T::mCherry* mESC reporter line (Deluz et al., 2016). Addition of either inhibitor resulted in smaller and shorter gastruloids, as well as reduced T::mCherry reporter activity (Fig. 6B,C, Fig. S8B,C, Table S3). In agreement with T::mCherry reporter activity, overall *T* mRNA expression was reduced in gastruloids treated with a MEK inhibitor compared to control (only 300 nM retinol) during the final 48 h of culture (Fig. 6C). These data show that FGF inhibition inhibits T expression, mesoderm differentiation and gastruloid elongation.

Next, we tested whether FGFb treatment could rescue the decrease of growth and mesoderm formation in gastruloids cultured with 600 nM retinol. We examined T::mCherry reporter activity and gastruloid size in gastruloids treated with 600 nM retinol with or without recombinant FGFb between 72 h and 120 h after aggregation (Fig. 6D). Although there were no significant differences in elongation, both gastruloid surface and T:mCherry reporter activity increased upon FGFb treatment (Fig. 6E,F, Table S3). Also, *T* mRNA expression was upregulated with FGFb (20 ng/ml) treatment during the final 48 h of culture compared to control (only 600 nM retinol) (Fig. 6F). These data revealed that FGFb supplementation rescued the effects of high-dose retinol treatment, resulting in increased T expression and enhanced mesoderm differentiation.

We also examined the effect of decreasing retinol concentration during elongation by culturing gastruloids with 600 nM retinol for 72 h and subsequently decreasing the concentration to either 300 nM or 0 nM (Fig. S9A). Even if gastruloids remained bigger in 600 nM, T::mcherry reporter activity increased in gastruloids switched to 0 nM retinol (Fig. S9B,C, Table S3). Consistent with the reporter activity, *T* mRNA expression was upregulated in gastruloids cultured without retinol (0 nM) during the final 48 h of culture compared to the control condition (600 nM retinol) (Fig. S9E). These data demonstrate that reducing retinol concentration mitigates the effects of high retinol treatment, restoring T expression, and promoting mesoderm differentiation.

To further investigate how modulation of signaling by retinol and FGF influences cell type composition, we performed scRNA-seq on *Rarb2-lacZ* gastruloids (Shen et al., 1992) and examined the effect of FGF inhibition, FGFb supplementation, or retinol withdrawal during the final 48 h of culture (see Materials and Methods) (Fig. 6A,D, Fig. S9A) on the distinct cell populations identified in Fig. 3 (Fig. 6G, Fig. S9D). Inhibition of FGF signaling (in 300 nM retinol) resulted in a decrease in the proportion of NMPs and mesoderm-derived populations, including PSM, somite and differentiated somite. In contrast, it increased the percentage of ectodermal cell populations: spinal cord populations (clusters 1 and 2) (Fig. 6G, Fig. S7A). These findings are consistent with previous data showing that MEK inhibition results in shortening of gastruloids without markedly impairing neural differentiation (van den Brink et al., 2014, 2020). In line with these observations, our data indicate that inhibition of FGF/MEK signaling primarily affects axial elongation and generation cells of mesodermal origin, while permitting or even relatively favoring neural lineage expansion.

FGFb supplementation (in 600 nM retinol) increased the proportion of NMPs, mesoderm-derived populations (including somite and differentiated somite), as well as specific spinal cord populations (clusters 1 and 4). In contrast, the proportions of other spinal cord subtypes (clusters 2 and 3) were reduced (Fig. 6G, Fig. S7B). Similar effects were seen when the retinol was removed during elongation (Figs S7C and 9D), suggesting that removal of retinol phenocopies addition of FGFb treatment in 600 nM retinol.

**Fig. 7.**
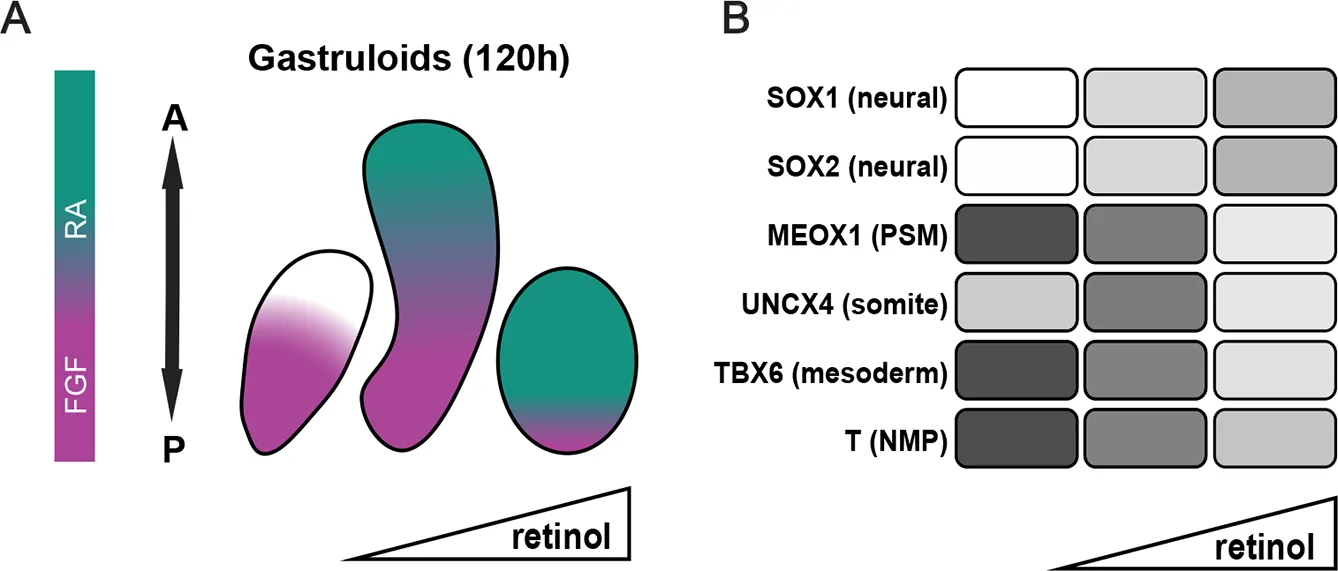
**Model for the action of RA on cell specification.** (A) Schematic depicting FGF and RA signaling polarization in mouse gastruloids 120 h after aggregation and the effect of different concentrations of retinol on the relative amount of FGF and RA signaling. A, anterior; P, posterior. (B) The effect of retinol on key ectoderm (SOX2, SOX1) and mesoderm (T, TBX6, MEOX1 and UNCX4.1) markers.

Together, these data show that FGF inhibition mimics the effects of high retinol levels, highlighting the key role of FGF signaling in gastruloid elongation and mesoderm–ectoderm balance. These data are summarized in a model, showing FGF and RA signaling polarization in mouse gastruloids (Fig. 7A), how RA can decrease FGF signaling and how interaction between these signaling pathways influence mesoderm-ectoderm cell fate decisions (Fig. 7B). Our data show that cellular identity can be shifted with retinol towards a more ectodermal state and provide an in-depth analysis of the associated cell types. Together, these findings suggest that RA antagonizes FGF signaling, suppressing mesodermal programs while promoting neural lineage specification during gastruloid development.

## DISCUSSION

During embryonic development, the generation of signaling gradients ensures proper development (Bernheim and Meilhac, 2020; Duester, 2008; Morgani and Hadjantonakis, 2020). For example, depending on the stage of development, RA promotes neural differentiation and mesoderm specification (Bernheim and Meilhac, 2020; Duester, 2008). However, dynamic modulation of RA and dissecting its spatiotemporal effects *in vivo* is challenging (Duester, 2008). With the increased availability of stem cell models of early embryonic development (Veenvliet and Herrmann, 2021), it is crucial to evaluate whether they have the potential to recapitulate such gradients and their downstream phenotypic and genotypic consequences. In this study, we aimed to investigate the spatiotemporal effect of RA and FGF signaling during mouse gastruloid development. In previous studies involving gastruloids, the concentration of retinoids in the culture medium was not well defined and exogenous RA supplementation was reported as teratogenic (Mantziou et al., 2021). Excessive levels of RA can promote signaling activity in cells that normally remain unresponsive (Duester, 2008; Ghyselinck and Duester, 2019). Inspired by this, we employed a retinoid-free medium and systematically modulated the retinol concentration. Our approach allowed us to dissect the dose-dependent and temporal effects of retinol on axial elongation and cell fate commitment in gastruloids using bulk and scRNA-seq, as well as reporter-based time-lapse imaging.

Manipulating RA signaling by adding retinol influenced FGF signaling in gastruloids (Figs 5 and 6). We provide detailed evidence that RA inhibits FGF, providing a potential mechanism of action of retinol and demonstrating the dynamic interplay between RA and FGF signaling in gastruloids (Figs 5–7). This interplay affects the generation of progenitors in gastruloids 120 h after aggregation (Figs 3, 4 and 7). Reduced RA signaling shifted differentiation from ectodermal toward mesodermal fates, as shown by the presence of paraxial mesoderm and somite-like cell types in the absence of retinol, which are progressively replaced by neural progenitors at higher concentrations (Figs 3 and 4, Figs S5, S7, S9). Retinol concentration thus regulates both cell type composition and morphology in gastruloids, highlighting possible crosstalk between differentiation and growth. In the absence of retinol, gastruloids were small and predominantly mesodermal, indicating limited cellular diversity and expansion (Figs 3 and 4). Intermediate retinol (300 nM) generated the largest gastruloids with a diverse mix of mesodermal and ectodermal cell types (Figs 3 and 4), supporting coordinated growth. In contrast, high retinol (600-1200 nM) drove neural dominance, resulting in smaller and simpler structures (Figs 3 and 4), likely due to reduced cellular diversity.

Altogether, these findings demonstrate that key aspects of cellular identity and diversity can be studied using gastruloids, as proven by our precise manipulation of RA (Fig. 7). This simple yet powerful modification highlights the versatility of the gastruloid model for studying early developmental processes and directing specific lineage outcomes.

### Study limitations

To minimize potential cell line-specific biases, we tested our approach across five different mESC lines. While the effect of retinol on gastruloid elongation and ectodermal and mesodermal identity was similar for all mESC lines tested, we observed that the optimal concentration for maximal elongation varied between 300 and 600 nM. Therefore, while the overarching biological trends are conserved, the specific retinol concentrations must be optimized for each individual cell line to achieve the best results.

## MATERIALS AND METHODS

### Cell culture

E14-IB10 mESCs (Netherlands Cancer Institute), *Rarb2-lacZ* mESCs (Shen et al., 1992), *T::H2B-mCherry* and *Sox2::H2B-Venus* mESCs (Veenvliet et al., 2020), *Sox1::eGFP T::mCherry* mESCs (SBR) (Deluz et al., 2016), *Uncx4.1::NeonGreen* and *Sox2::mScarlet3* mESCs were seeded on 0.1% gelatin (Sigma-Aldrich, G1393) pre-coated plates (Falcon, 353046), cultured with mESC medium consisting of Knock-out DMEM (Gibco, 10829018), 15% fetal bovine serum (Serana, S-FBS-CO-015), 1% NEAA (Gibco, 11140035), 1% GlutaMAX Supplement (Gibco, 35050038), 50 μM 2-mercaptoethanol (Gibco, 21985023) and 1000 U/ml mLIF (Sigma-Aldrich, ESG1107) and incubated in a humidified incubator with 5% CO_2_ at 37°C. mESC medium was changed daily, and the cells were passaged every other day with TrypLE™ Express Enzyme (Gibco, 12604021). The cells were tested for *Mycoplasma* monthly and reported negative.

### Generation of *Aldh1a2^−/−^ Rarb2-lacZ* mESCs

*Rarb2-lacZ* mESCs (Shen et al., 1992) were electroporated with Cas9 protein together with a guide targeting *Aldh1a2* with the sequence 5′-AGATCTTTATTAACAATGAA-3′ as well as a plasmid containing GFP with a Lonza electroporator and Lonza mESC electroporation kit, using the manufacturer's settings. GFP-positive cells were sorted, and the *Aldh1a2* sequences of colonies were analyzed using TIDE software to verify indels that abrogate *Aldh1a2* expression. We analyzed two *Aldh1a2*-negative clones for the absence of *Rarb2-lacZ* X-gal staining and used one for this study. Both control and knockout cells were treated with 1 μM RA for 16 h, stained with X-gal, and showed RA-dependent *lacZ* reporter activity.

### Generation of *Uncx4::NeonGreen* mESCs

We generated a construct to target the *Uncx4.1* (*Uncx*) gene and incorporate NeonGreen behind *Uncx4.1*, separated by T2A, allowing cleavage of *Uncx4.1* and NeonGreen. We amplified homologous arms of the *Uncx4.1* gene flanking the incorporation site using the following primers: left arm: 5′-TCGCAAGGAACTGGAGAAGA-3′ and 5′-GTCCATGTCCACCTCCTCGC-3′; right arm: 5′-TGAGCTGGGGACCGCGGC-3′ and 5′-CTAGAAATCGGTAATGTATA-3′; PCR from E14 gDNA. T2A_Neon-green-polyA (with 15 bp overlap with left arm): 5′-GTGGACATGGACGATATCGAGGGCAGAGGAAGTCTTCTAACATGCGGTGACGTGGAGGAGAATCCCGGCCCTGTGAGCAAGGGCGAGGAG-3′ and 5′-CTAGAGGTCCATAGAGCCCACCGCA-3′; PGK-neomycin-polyA: 5′-TCTATGGACCTCTAGAGTCAACGAAG-3′ and 5′-GTCCCCAGCTCAGGATCCATTCGACTCTAGAACGAA-3′ (with 15 bp overlap with right arm). Fragments were assembled in a vector based on pDTA-TK (Addgene plasmid #22677) with HiFi assembly. E14 mESCs were electroporated with this construct together with Cas9 protein and *Uncx4.1* guide (Synthego) with the targeting sequence 5′-GGTGGACATGGACTGAGCTG-3′ using a Lonza electroporator and Lonza mESC electroporation kit, using the manufacturer's settings. Cells were selected with 50 mg/ml G418 (Sigma-Aldrich, G9570) until resistant clones appeared. Heterozygotic clones were tested for correct integration of the targeting construct and the correct sequence of *Uncx4.1* of the chromosome, where the construct was verified.

### Generation of *Uncx4::NeonGreen*, *Sox2::mScarlet3* mESCs

We generated the *Sox2::mScarlet3*, in the *Uncx4.1::NeonGreen* background cell line. Two copies of mScarlet3, separated by T2A or P2A, were cloned behind the coding sequence of *Sox2*, generating the following construct Sox2::T2A-mScarlet3-P2A-mScarlet3 using the following primers: Sox2 left arm: 5′-CCATGAACGCCTTCATGGTATG-3′ and 5′-CATGTGCGACAGGGGCAGT-3; Sox2 right arm: 5′-TGAGGGCTGGACTGCGAACTG-3′ and 5′-GGTAGGATTGAACAAAAGCTATTATAAATTACCAACG-3′, using E14 mESC gDNA. PCR products of mScarlet3 containing 5′ either T2A or P2A were obtained and assembled with Sox2 arms using primers that contain 30 bp overlap into a vector based on pDTA-TK (Addgene plasmid #22677) containing the blasticidin resistance gene, using HiFi assembly. Electroporation using Sox2-specific guide with the sequence 5′-CTGCCCCTGTCGCACATGTG-3′ was done in E14 mESCs using a Lonza Nucleofector electroporator and mESC nucleofector kit, using the manufacturer's settings. Cells were selected with 4 mg/ml blasticidin (InvivoGen, ant-bl-05) until resistant clones appeared. Heterozygotic clones were tested for correct integration of the targeting construct and the sequence of *Sox2* of the non-integrated chromosome was verified.

### Preparation of retinoid-free N2B27 medium

Retinoid-free N2B27 medium was prepared with the 1:1 mix of Neurobasal Medium (Gibco, 21103049) and DMEM/F-12 no glutamine (Gibco, 21331020), supplemented with 1% NEAA (Gibco, 11140035), 1% GlutaMAX Supplement (Gibco, 35050038), 1 mM sodium pyruvate (Gibco, 11360039), 0.5× B-27™ Supplement minus vitamin A (Gibco, 12587010) and 0.5× N-2 Supplement (Gibco, 17502048). The desired concentration of retinol (Sigma-Aldrich, R7632) was adjusted as described in each experiment. Dimethyl sulfoxide (DMSO) (Sigma-Aldrich, 472301) was employed as a negative control, as retinol was dissolved in it.

### Gastruloid generation

Gastruloids were generated according to the standard protocol with minor modifications (Beccari et al., 2018; Van Den Brink et al., 2014). mESCs with 70-80% confluency was washed with DPBS (Gibco, 14190-094) twice, treated with TrypLE™ Express Enzyme (Gibco, 12605028) and incubated for 3 min at 37°C. The cells were dissociated into single cells by pipetting up and down ten times. The cell suspension was made up to 10 ml with mESC medium and was centrifuged at 130 ***g*** for 3 min. The supernatant was discarded, and cells were washed with 10 ml DPBS and centrifuged at 130 ***g*** for 3 min. This washing step was repeated. After the last wash, the cells were dissociated into single cells in N2B27 medium and counted with a hemocytometer (Neubauer). The cells were resuspended at a concentration of 10 cells/μl in N2B27 containing the desired concentration of retinol. Then, 40 μl of cell suspension was seeded in each well of a 96-well plate (Greiner bio-one, 650185) with a multichannel pipette (Sartorius). At 48 h after aggregation, 150 μl N2B27 medium supplemented with 3 μM CHIR99021 (chiron) (Stem Cell Technologies, 72054) and the desired concentration of retinol (Sigma-Aldrich, R7632) was added to the gastruloids. At 72 h after aggregation, 150 μl supernatant was replaced with 150 μl fresh N2B27 medium with the desired concentration of retinol. At 96 h after aggregation, the previous step was repeated, and at 120 h aggregation gastruloids were collected and used for further assays.

### Treatment of gastruloids with small molecules and proteins

The concentration of retinol (Sigma-Aldrich, R7632) was adjusted in retinoid-free N2B27 medium depending on the experiments. DMSO was applied as a control. To modulate FGF signaling, gastruloids were generated with the *T::mCherry* mESC line (Deluz et al., 2016). To analyze the effect of FGF signaling inhibition, gastruloids were grown in 300 nM retinol. For FGF receptor inhibition between 72 h and 120 h after aggregation, 0.05 μM or 0.1 μM ingifratinib (Guagnano et al., 2011) (Selleckchem, S2183) was used; for MEK inhibition, 0.5 μM or 1 μM mirdametinib (Barrett et al., 2008) (Axon Medchem, 1408) was applied between 72 h and 120 h after aggregation. DMSO (Sigma-Aldrich, 472301) was applied as a control. To activate FGF signaling, gastruloids were grown in 600 nM retinol, and 10 ng/ml or 20 ng/ml FGFb (Thermo Fisher Scientific, PMG0035) was applied between 72 h and 120 h after aggregation. PBS was used as a control.

### Imaging and image analysis for morphology parameters

Representative images of gastruloids were obtained with a stereo microscope (Leica M205 FCA) 120 h after aggregation. The brightness and contrast of each image were adjusted separately as they were not used for the image analysis.

For image analysis, gastruloids were imaged in 96-well plates with IncuCyte S3 (Sartorius) 120 h after aggregation. Image masks were created with IncuCyte S3 Image analysis software with the ‘Spheroid’ module. Some masks were removed from the analysis manually if there was more than one object, and it was not representative of the real image. Next, we performed image analysis in Fiji (Schindelin et al., 2012) with the created masks to measure the gastruloid width, length and length/width ratio using a previously published pipeline (Girgin et al., 2021). Graphs were made in GraphPad Prism 10.2.3. Statistical analysis was performed with GraphPad Prism 10.2.3. First, a one-way ANOVA was applied, and the mean of each experimental group was compared with the mean of every other experimental group. Then, the Tukey test was applied for the correction of multiple comparisons with a 95% confidence interval.

### Time-lapse imaging, image analysis and statistics

The *Sox2::Venus T::mCherry* (Veenvliet et al., 2020) double reporter cell line was highly sensitive to Incucyte S3 conditions and failed to form gastruloids. Therefore, two alternative cell lines, *T::mCherry* (Deluz et al., 2016) and *Sox2::mScarlet3* reporter mESCs (this study), were used for time-lapse imaging. Time-lapse imaging was performed in 96-well plates with Incucyte S3 (Sartorius). Images of gastruloids were captured every 4 h with or without channels (red), depending on the experiments. Image analysis was performed using the commercially available Incucyte S3 Spheroid Image Analysis Module (Sartorius). Three parameters were assessed: gastruloid surface area was measured as the ‘largest brightfield object area (μm^2^)’, elongation index was quantified using the ‘brightfield object average eccentricity’, and reporter activity was determined as the ‘largest brightfield object red/green integrated intensity (RCU×μm^2^)’. The image masks, which are not representative of real gastruloids, were discarded from the analysis manually. At time points involving medium changes or treatment with small molecule inhibitors or proteins, gastruloids were displaced from the center of the wells, impairing image focus. This resulted in transient drops in signal that represent technical artifacts rather than biological effects. In some biological replicates, individual time points were missing due to imaging errors and were therefore excluded from the analysis. The data were obtained from Incucyte and was normalized in GraphPad Prism 10.2.3 such that the minimum value was 0 and the maximum value was 1. After the normalization of data, biological replicates were pooled, graphs were generated, and statistics were performed in GraphPad Prism 10.2.3.

For statistical analysis of time-lapse imaging, the mixed effect model with Geisser–Greenhouse correction was applied, by which the mean of each time point was compared with the mean of the other experimental group. Statistics only for the latest time point are reported on the graphs. However, statistics for each time point can be found in Table S3.

### Immunostaining, imaging, and image analysis

Gastruloids generated from reporter cell lines were collected and washed twice with ice-cold PBS. Samples were fixed overnight at 4°C in 1% paraformaldehyde (PFA) (Thermo Fisher Scientific, 28908). The following day, gastruloids were washed three times with PBS and incubated with Hoechst 33342 (1:5000 in PBS) (Tocris, 5117) overnight at 4°C. After staining, samples were washed three times and kept in PBS until microscopy.

For gastruloids lacking fluorescent reporters, samples were collected in tubes, washed three times with PBS, and fixed overnight at 4°C in 4% PFA in PBS. The following day, samples were washed three times with PBS and stored at 4°C until staining. For permeabilization, gastruloids were incubated overnight in 0.5% Triton X-100 (Sigma-Aldrich, X100). The permeabilization solution was then replaced with blocking solution [0.2% Triton X-100, 0.1% Tween-20 (Sigma-Aldrich, P1379) and 2% bovine serum albumin (Sigma-Aldrich, A3059) in PBS] and incubated overnight at 4°C. Samples were then incubated with primary antibodies (Table S8) together with Hoechst for 4 days at 4°C. Following primary incubation, gastruloids were washed four times for 1 h each with blocking solution. Secondary antibodies and Hoechst were added at the concentrations indicated in Table S8 and incubated overnight at 4°C. The next day, samples were washed four times for 1 h each with blocking solution at room temperature, followed by two 15-min washes with PBS.

Gastruloids were mounted in FastWell chambers (Merck, GBL666117) with 80% glycerol (Sigma-Aldrich, G5516) in PBS for imaging. Imaging was performed using an Andor Dragonfly 200 or Dragonfly 500 confocal microscope (Oxford Instruments). Representative images were processed using ImarisViewer 10.0.1.

Image analysis was performed using Fiji (Schindelin et al., 2012). Signal intensity along the gastruloid midline was measured using the ‘Line’ tool from the posterior to the anterior end. Channels were separated, and intensity profiles were extracted using the ‘Plot Profile’ function. Data were exported to Excel, and gastruloid length was normalized (minimum=0, maximum=1). Intensity values were pooled and normalized (minimum=0, maximum=1) using GraphPad Prism. Graphs were generated in Python using a custom script.

### X-gal staining for RA-dependent β-gal assay and microscopy

X-gal staining was performed according to the protocol described by Foley and O'Farrell (2003) with some modifications. Gastruloids were collected and washed with ice-cold DPBS (Gibco, 14190-094) three times. They were fixed on ice for 5 min in fixation solution consisting of DPBS supplemented with 1% PFA (Thermo Fisher Scientific, 28908), 0.2% glutaraldehyde (Merck, G6257), 2 mM MgCl_2_ (Merck, M2670), 5 mM EGTA (pH 8.0) (Sigma Aldrich, 324626), 0.2% Nonidet-P40 (NP40) (Thermo Fisher Scientific, 85124). The fixation solution was removed, and gastruloids were washed three times with the washing solution consisting of DPBS supplemented with 2 mM MgCl_2_, 0.01% sodium deoxycholate (Merck, 30970), 0.02% IGEPAL CA-630 (Sigma-Aldrich, I8896). After the washing step, the gastruloids were incubated at 37°C for 48 h in a staining solution consisting of 5 mM potassium hexacyanoferrate (II) trihydrate (Sigma-Aldrich, P3289) 5 mM potassium ferricyanide (Acros Organics, 22311), 2 mM MgCl_2_ (Merck, M2670) 0.01% sodium deoxycholate (Merck, 30970), 0.02% NP40 (Thermo Fisher Scientific, 85124) and 0.1% X-gal (Thermo Fisher Scientific, R0404). After color development, the gastruloids were washed with DPBS three times, and they were refixed overnight at 4°C in 4% PFA. The next day, PFA was removed, and gastruloids were washed with PBS three times. They were mounted in 80% glycerol, and images were obtained with a Leica M165 FC stereo microscope.

### X-gal and double immunostaining

Following X-gal staining (see ‘X-gal staining for RA-dependent β-gal assay and microscopy’ section), immunostaining was performed (see ‘Immunostaining, imaging, and image analysis’ section). HRP-conjugated secondary antibodies (Table S8) were used for detection.

After the final wash, gastruloids were rinsed twice in Tris-Mal buffer (pH 7.6) for 5 min each. For color development, samples were incubated in staining solution containing 10 ml Tris-Mal buffer (pH 7.6), 0.1% DAB substrate (Sigma-Aldrich, D5637) and 0.03% H_2_O_2_ (Merck, 1072100250). Color development was monitored and stopped after approximately 2 min by washing the gastruloids with blocking solution (see ‘Immunostaining, imaging, and image analysis’ section), followed by three washes in PBS. Images were acquired using a Leica M65 stereomicroscope.

### Cell dissociation for FACS and scRNA-seq

Gastruloids were collected on ice and washed with PBS three times at room temperature. They were incubated with TrypLE Express (Gibco, 12605028) for 4 min at 37°C in a heating block with shaking at 300 rpm, followed by pipetting up and down ten times. This step was repeated once more to achieve single-cell dissociation. After incubations, the gastruloids were dissociated into single cells by pipetting up and down ten times. The cells were washed with PBS twice, centrifuged at 130 ***g*** for 5 min at 4°C, and the supernatant was carefully discarded without disturbing the pellet. The cells were resuspended in PBS containing 0.04% bovine serum albumin (Sigma-Aldrich, A3059) and 1:5000 DAPI (Invitrogen, D1306) as a viability dye. The cell suspension was filtered through a FACS tube (Falcon, 352235), and FACS analysis was performed.

### FACS and statistical analysis

Gastruloids were generated using different dual-reporter mESC lines to monitor lineage specification, including the *Sox2::Venus; T::mCherry* mESCs (Veenvliet et al., 2020) *Sox1::eGFP;T::mCherry* (Deluz et al., 2016), and the newly generated *Sox2::mScarlet3; Uncx4.1::NeonGreen* mESCs (see ‘Generation of Uncx4::NeonGreen, Sox2::mScarlet3 mESCs’ section). At 120 h post-aggregation, 40-90 gastruloids per condition were collected and dissociated into a single-cell suspension. DAPI (Invitrogen, D1306) staining was performed to select live cells. *Rarb2*-*lacZ* (Shen et al., 1992) mESCs were used as a negative control. Compensation and gating controls were performed on undifferentiated mESCs, including single-reporter and double-reporter lines. FACS was performed with CytoFlex SRT (Beckman Coulter) or BD LSRFortessa (BD Biosciences) and analyzed with FlowJo™ v10.10.0 software (BD Life Sciences). Cells were first gated based on FSC-A/SSC-A to exclude debris, followed by singlet discrimination (FSC-W/FSC-H and SSC-W/SSC-H). DAPI-negative cells were selected to exclude dead cells. Quantification included both the percentage of positive cell populations and the raw median fluorescence intensity (MFI) for each reporter to assess relative expression levels. Statistical analysis and visualization were performed in GraphPad Prism 10.2.3. Differences between experimental groups were determined by a one-way ANOVA with Tukey's post-hoc test (95% confidence interval).

### Bulk RNA-seq

Gastruloids were generated from the *Rarb2-lacZ* mESC (Shen et al., 1992) reporter line with 0, 300, 600 and 1200 nM retinol. E14 *Rarb2-lacZ* mESCs were analyzed 24, 48, 72, 96 and 120 h after aggregation; gastruloids (approximately *n*=40-48 and *N*=3) were collected, washed with ice-cold PBS three times and 500 μl TRIzol (Thermo Fisher Scientific, 15596018) was added. The samples were dissociated using a p1000 pipette and stored at −80°C until RNA isolation. For RNA isolation, the samples were incubated at room temperature until defrosted. RNA extraction and the cDNA library preparation were performed as previously described with specific CELseq2 barcodes and TruSeq small RNA adaptors (Martinez Mir et al., 2024). DNA library size enrichment was performed by GenomeScan. Paired-end (150 bp) sequencing was carried out on the resulting libraries on an Illumina NovaSeq next-seq sequencing platform.

### Bulk RNA-seq data analysis

The first six bases of read 1 contained the unique molecular identifier (UMI), and the next eight bases contained the sample barcode (CELseq2), while read 2 contained the transcript information. Read 2s with a valid barcode were selected, trimmed using TrimGalore (v.0.6.6) with default parameters, and mapped using STAR (v.2.7.7a) with default parameters to the mouse GRCm38 genome (Ensembl 102). Resulting bam files were deduplicated using umi_tools (v1.1.4) with the option ‘dedup --method=unique’. A count table for all samples was generated using featureCounts (v.2.0.1) from subread-2.0.1-source, with options ‘-t exon -g gene_name’. Only gene entries appearing in at least three samples and representing at least five per million of the total counts were kept for downstream analysis. Next, data were normalized using the ‘varianceStabilizingTransformation’ function from the DESeq2 R package. Since samples were processed in three independent batches, the function ‘removeBatchEffect’ from the limma R package was used to remove batch effects. PCA, clustering, and heatmap representations were performed using the ‘PCA’ function from the Python ‘sklearn. decomposition’ library (v), the dendrogram and linkage functions from the Python ‘scipy.cluster.hierarchy’ library (v.1.11.1), and the Python ‘PyComplexHeatmap’ library (v.1.6.7), respectively. For each PC, the probability density function (pdf) of gene coefficients was extracted, and significantly contributing genes were defined as those with a coefficient value found in the top or bottom 5% of the pdf. GO analysis was performed using Metascape (Zhou et al., 2019), applying the ‘Express Analysis’ option with default parameters. Each gene list was submitted as a single input, and the top five enriched pathways were selected based on –log_10_(P-value). Heatmap visualizations were produced using the ‘pyComplexHeatmap’ package (Ding et al., 2023). Differential gene expression analysis between the different time points and retinol conditions was performed using the DESeq2 R package, using ‘∼ library+cond’ as a design, where ‘cond’ refers to the combination of time point and retinol concentration, and ‘library’ to the biological replicate.

### scRNA-seq

At 120 h after aggregation, 80-90 gastruloids from each experimental group were collected and dissociated into single cells (see ‘Cell dissociation for FACS and scRNA-seq’ section). Experimental groups consisted of gastruloids cultured with 0 nM, 300 nM or 600 nM retinol throughout the protocol. Additional conditions included: (1) gastruloids cultured with 300 nM retinol supplemented with 1 μM mirdametinib during the final 48 h of culture; (2) gastruloids cultured with 600 nM retinol supplemented with 20 ng/ml FGFb during the final 48 h; and (3) gastruloids cultured with 600 nM retinol and subsequently transferred to 0 nM retinol medium for the final 48 h of the protocol. As mirdametinib was dissolved in DMSO and FGFb in PBS, the same DMSO/PBS vehicle mixture was added to all other experimental and control groups during the final 48 h of culture. Live single cells were sorted using FACS (Aria 3). DAPI-negative and singlet populations were gated and collected (see ‘FACS and statistical analysis’ section). scRNA-seq was performed with 10x Chromium Genomics GEM-X Universal 3'v4 chemistry with on-chip multiplexing (OCM) of eight samples at the Leiden Genome Technology Center (LGTC), the genomics and transcriptomics facility at LUMC.

### scRNA-seq data preprocessing

scRNA-seq data were processed using Cell Ranger (10x Genomics, v.7.1.0) for alignment, barcode assignment and gene quantification. Reads were aligned to a custom reference genome created by adding the *lacZ* and neomycin genes to the standard mouse reference from 10x Genomics (refdata-gex-mm10-202-A) according to 10x instructions. scRNA-seq data were processed in scanpy (v.1.9.5) (Wolf et al., 2018). For each sample, cells were filtered based on total UMI counts (log10>3.5-3.7), genes detected (>1350-2100) and mitochondrial gene fraction (<10%). Doublets were identified using the Scrublet package (v.0.2.3) (Wolock et al., 2019), and kept for a first analysis following the scanpy workflow. Subsequently, all cells belonging to clusters in which more than 50% of the cells are doublets were removed, together with all cells that Scrublet annotated as doublets.

### Integration of scRNA-seq data with published data and analysis

The data generated in this study were analyzed together with previously published scRNA-seq data from van den Brink et al. (2020). The 10x Chromium data from this study were downloaded from GSE123187 and mapped to our custom reference genome using Cell Ranger as described above. Initially, differentially expressed genes (Wilcoxon-rank sum test, log fold change >2 and adjusted *P*<1e−3) between both datasets were excluded from the analysis and the standard scanpy workflow was applied, with the only addition of regressing out the experimentalists (i.e. this paper versus van den Brink et al., 2020) with the function ‘tool.regress_out’ from scanpy. Close inspection of the PCA revealed that PC2 split cells according to dataset source. Therefore, PC2 was ignored from downstream analysis (e.g. nearest neighbor computation, UMAP, and Leiden clustering). The resulting UMAP was used to visualize our data. Each Leiden cluster was assigned a cell type identity based on the fraction of cells from the Brink et al. study that it contained. The whole transcriptome, including genes differentially expressed between the two datasets, was used for differential gene expression analysis. Wilcoxon-rank sum test was used, with log fold change >1 and adjusted *P*<0.001.

## Supplementary Material



10.1242/develop.205153_sup1 Supplementary information

Table S1. The dynamic effect of retinol in gene expression during gastruloid culture. This table includes the list of genes significantly contributing to PC1 or PC2 with the corresponding sign and cluster assignation.

Table S2. Differentially expressed genes (DEGs) across retinol concentrations and time points (24 h and 120 h) after aggregation. The table lists DEGs identified across retinol conditions and developmental time points.

Table S3. Statistics for Incucyte S3. This table contains the statistics for image analysis of the images obtained from Incucyte S3.

Table S4. Differentially expressed marker genes for reference gastruloid cell clusters. List of marker genes for the different cell type clusters identified in the reference gastruloid scRNA-seq dataset (van den Brink et al., 2020b). For each gene, the table provides the log2 fold change, p-value, adjusted p-value and cluster assignment score.

Table S5. Gene Ontology (GO) enrichment analysis of gastruloid cell types. This table identifies the biological processes significantly enriched among the differentially expressed genes. Results include the p-value, adjusted p-value, and odds ratio for each term, as well as the specific genes contributing to the enrichment.

Table S6. Differentially expressed marker genes from the reference mouse embryo atlas. List of marker genes for the embryonic cell type clusters identified in the mouse embryo atlas (Pijuan-Sala et al., 2019). For each gene, the table provides the Ensembl ID, gene symbol, log2 fold change, p-value, adjusted p-value, and cluster assignment score.

Table S7. Gene Ontology (GO) enrichment analysis of embryo-atlas derived cell populations. Significant biological process GO terms associated with the marker genes identified from the Pijuan-Sala et al. (2019) reference. This table identifies the biological processes significantly enriched among the differentially expressed genes. Results include the p-value, adjusted p-value, and odds ratio for each term, as well as the specific genes contributing to the enrichment.
