# Supplementary material for: Retinoic acid and FGF signaling interact to control elongation and lineage specification in a mouse gastruloid model
Source: Development. 2026 Aug 13;153(15):dev205153. doi: 10.1242/dev.205153 (PMC13535096; doi:10.1242/dev.205153)
Supplement: Supplementary file 1 [file develop-153-205153-s1.pdf]

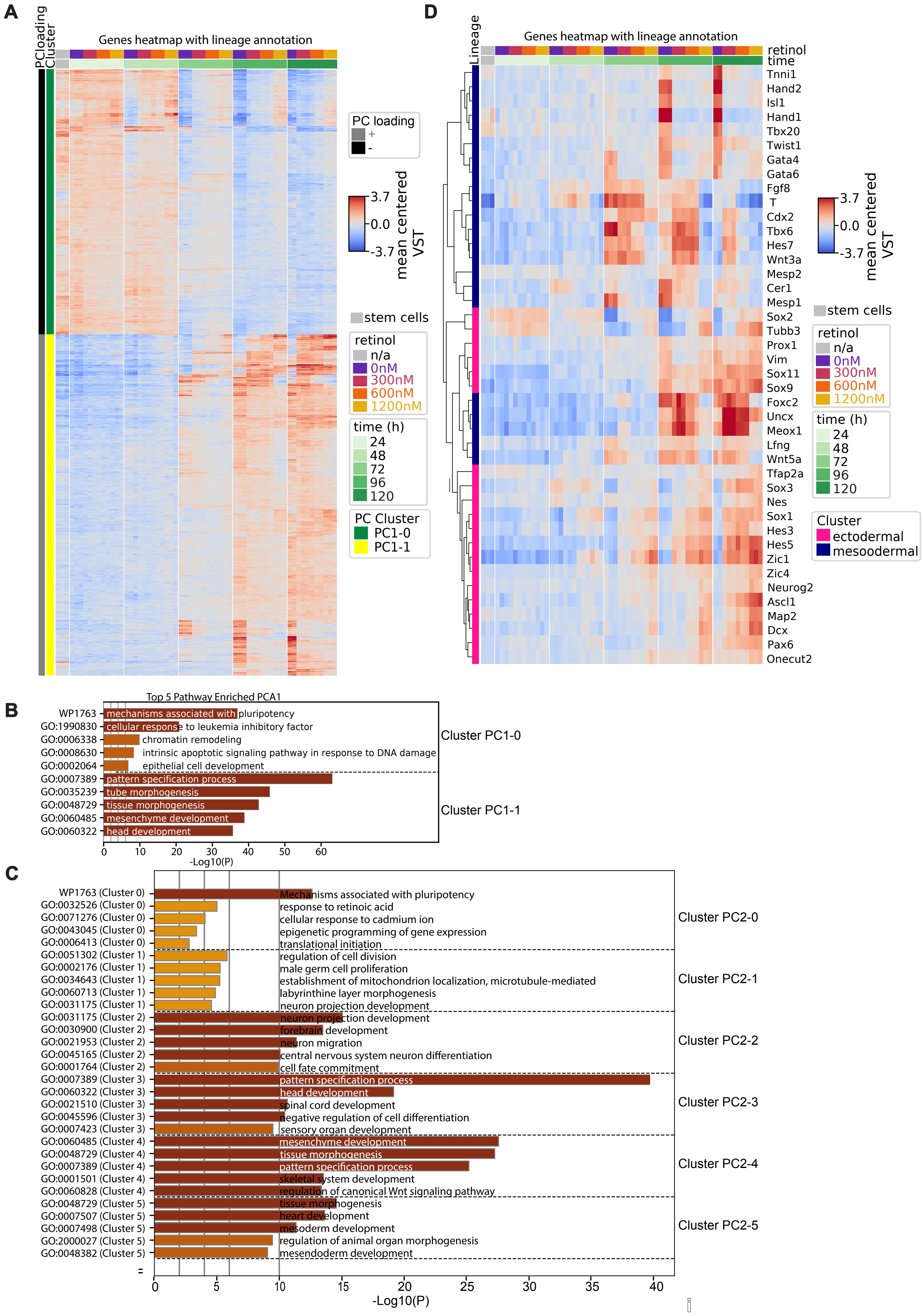


**Fig. S1. Time course bulk RNA sequencing of gastruloids treated with different doses of retinol.**

(A)Heatmap showing time course RNAseq data of genes contributing to PC1, clustered according to gene expression patterns. (B) Metascape analysis of each of the two clusters obtained for genes contributing to PC1 based on their time course RNAseq expression patterns. (C) Metascape analysis of each of the 6 clusters obtained for genes contributing to PC2 based on time-course RNAseq expression patterns. (D) Time course RNA-seq data of selected mesodermal and ectodermal-related genes.

Development • Supplementary information

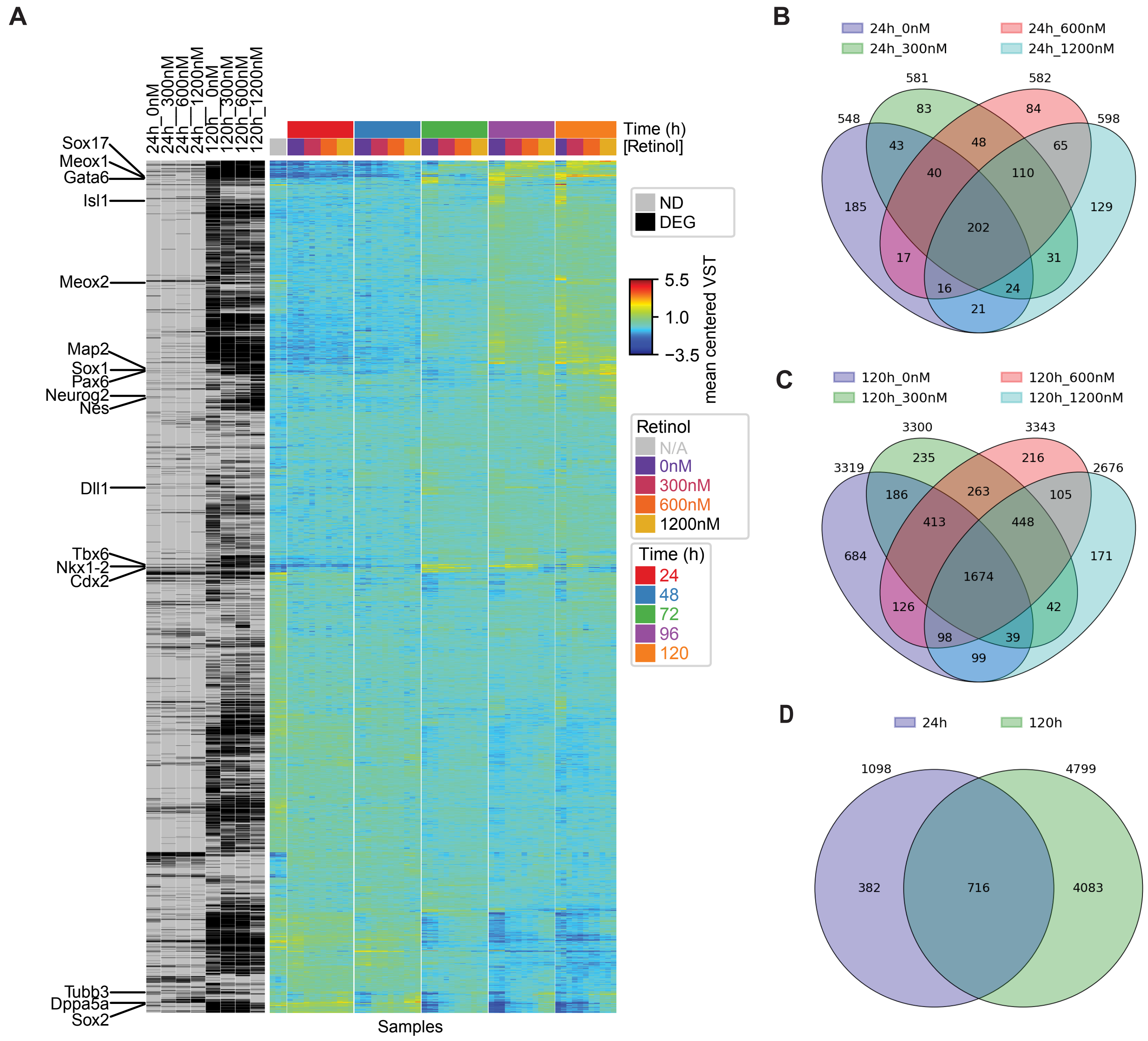


**Fig. S2. Differential gene expression dynamics across retinol conditions and time points.**

(A) Heatmap showing the mean-centered VST across different time points and retinol concentrations for genes differentially expressed between one or more samples or compared to stem cells. Genes differentially expressed in one sample are indicated in black in the left annotation bars; others are indicated in grey. Differential expression annotation is shown for samples obtained at 24h and at day 120h. (ND: non-differentially expressed; DEG: differentially expressed genes) (B) Venn diagram comparing the numbers of differentially expressed genes with different retinol concentrations at 24h after aggregation. (C) Venn diagram comparing the numbers of differentially expressed genes with different retinol concentrations at 120h after aggregation. (D) Venn diagram showing the intersection between pooled differentially expressed genes identified at 24 h and 120 h after aggregation across all retinol conditions.

Development • Supplementary information

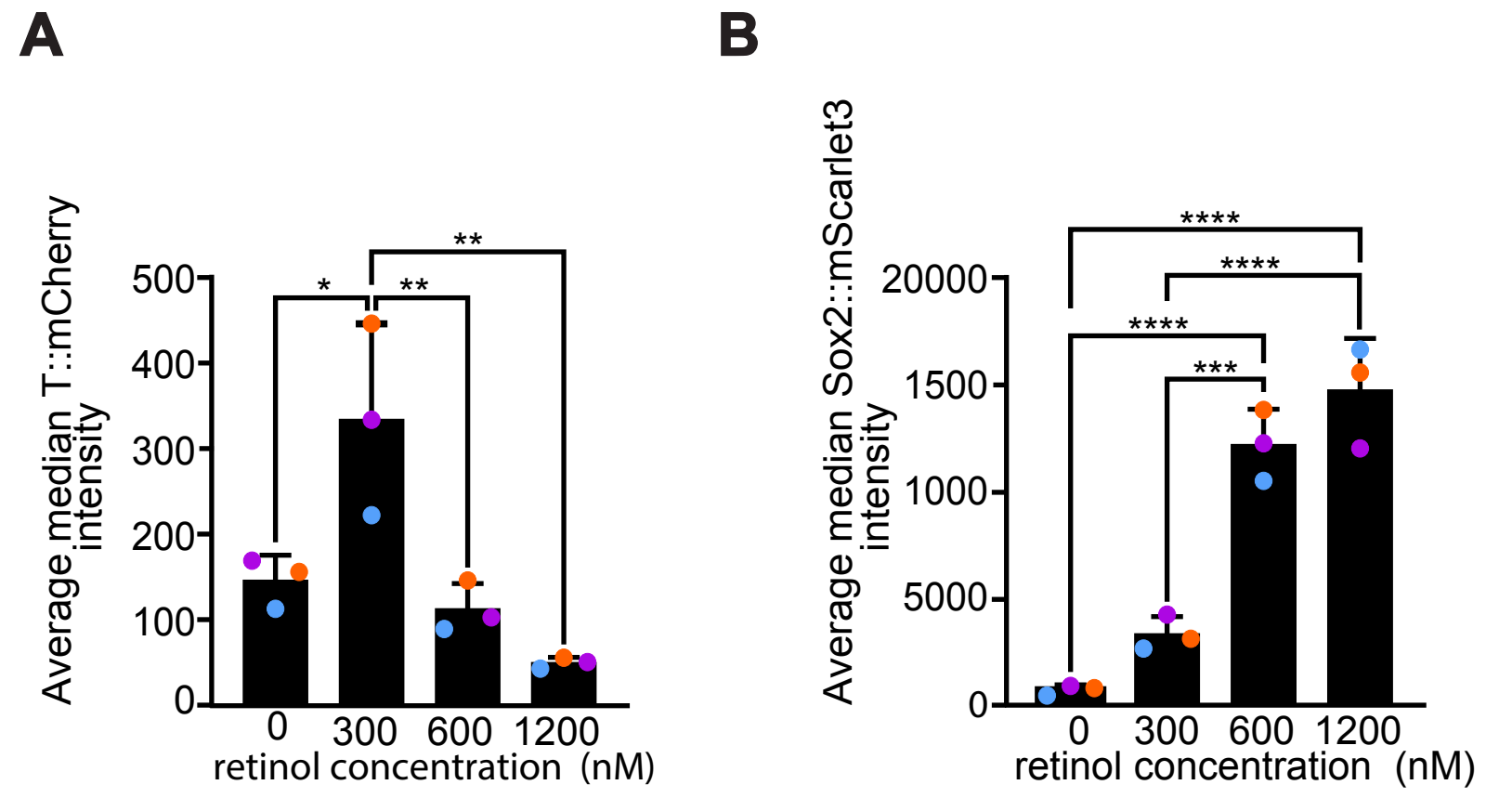


**Fig. S3. Average median intensity of T::mCherry and Sox2::mScarlet3 positive cells.**

(A) Bar graphs showing the average median intensity of T::mCherry (from the T::mCherry; Sox1::GFP cell line) and (B) Sox2::mScarlet3-positive cells in gastruloids treated with different doses of retinol, as measured by FACS 120 h after aggregation. 40–45 gastruloids were used for each experimental group (N = 3). Each color represents a biological replicate. Graphs show mean ± SD. Experimental groups were compared using one-way ANOVA, and P-values were adjusted using Tukey's multiple comparisons test.

*p<0.05, **p<0.01, ***p<0.001, ****p<0.0001 for both graphs.

Development • Supplementary information

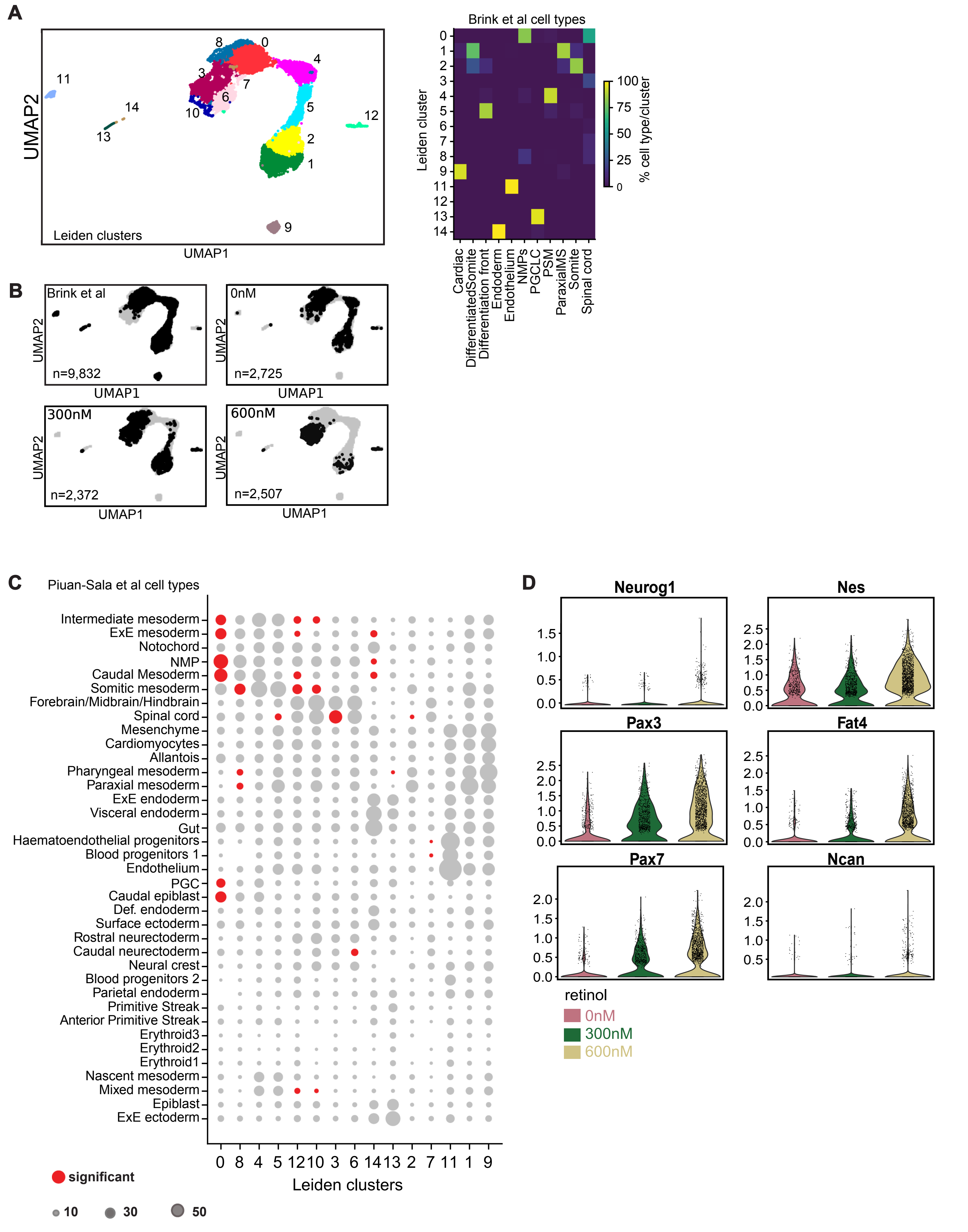

**Fig. S4. Label transfer for cell type identification and the effect of retinol on cell clusters**

(A) (left) UMAP of the integrated scRNA-seq datasets from this study (generated using the 10x Chromium platform) and Brink et al., with cells colored by Leiden cluster identity. (right) Heatmap showing, for each annotated cell type, the fraction of cells assigned to each Leiden cluster. This analysis is restricted to cells from the Brink et al. dataset. (B) Same UMAP, where cells from Brink et al. or from the different retinol culture conditions in this study are highlighted in black, and all other cells are shown in grey. (C) Dot plot showing the number of shared markers between the Leiden clusters in panel A and the annotated cell types from the scRNA-seq mouse embryo atlas by Pijuan-Sala et al. Red indicates significant, whereas grey indicates non-significant results (p-value < 0.001). The dot size is proportional to the number of shared markers. (D) Violin plots showing expression of neuronal markers and the effect of different retinol concentrations.

Development • Supplementary information

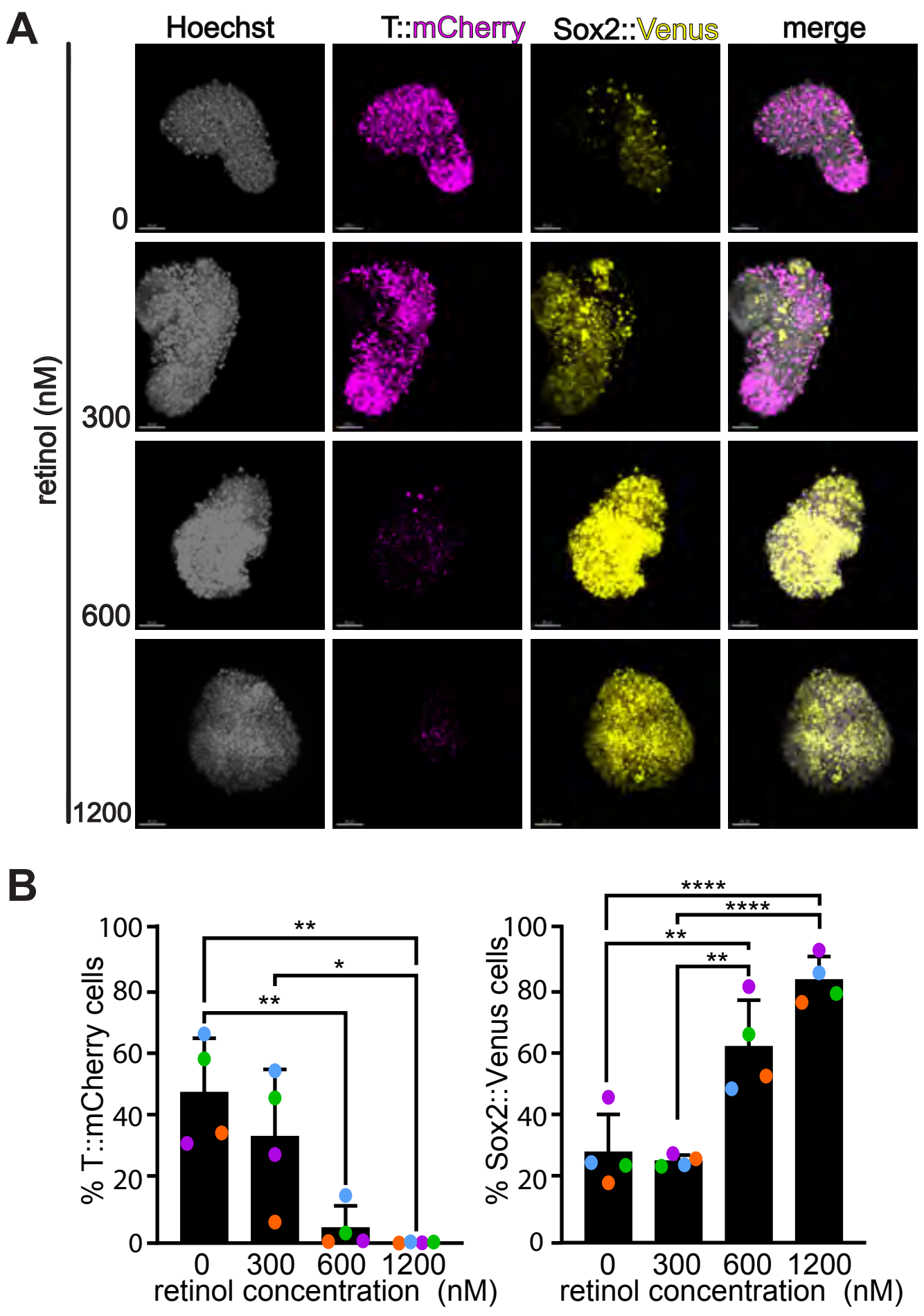


**Fig. S5. The effect of retinol on T::mCherry and Sox2:: enus reporter activity in gastruloids**
**(A)** Representative images of 120 h gastruloids expressing T::mCherry (magenta) and Sox2::Venus (yellow) treated with different concentrations of retinol. Grey indicates Hoechst staining. Scale bars: 50 µm. **(B)** Percentages of T::mCherry- and Sox2::Venus-positive cells in 120 h gastruloids treated as in (A), as determined by FACS. A total of 40–45 gastruloids were analyzed per experimental group (N = 4). Each color represents a biological replicate. Graphs show mean ± SD. Statistical comparisons between groups were performed using one-way ANOVA followed by Tukey's post hoc test. * p< 0,05; ** p< 0,005; **** p< 0,0001for both graphs.

Development • Supplementary information

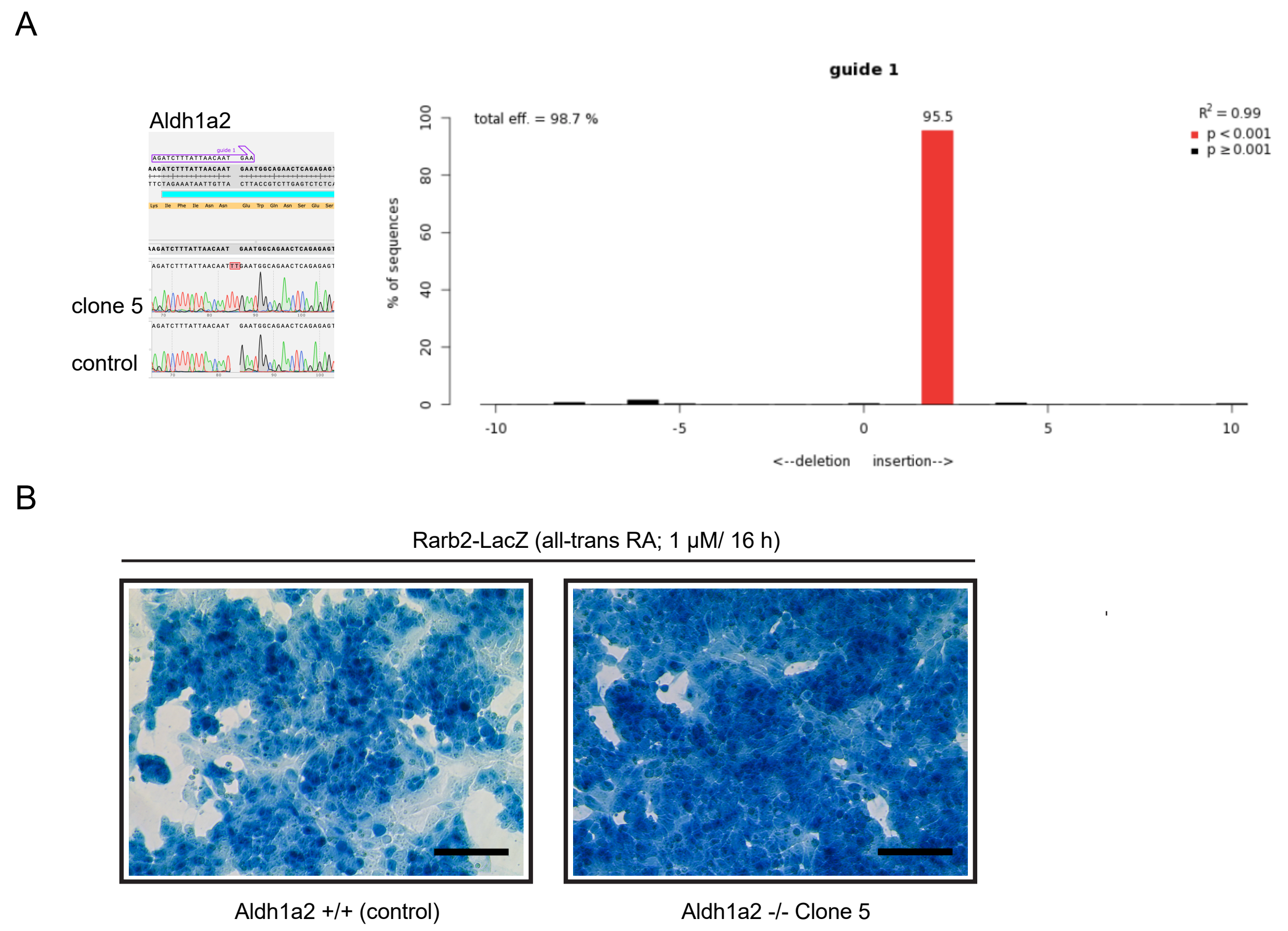


**Fig. S6. Generation of *Aldh1a2$^{-/-}$ Rarb2-lacZ* mESCs**

(A) (left) Sequence comparison of *Aldh1a2* between control *Rarb2-LacZ* mESCs and *Aldh1a2* knockout cells, containing a 2 bp insertion. (right) TIDE analysis showing indel formation at the target locus, confirming efficient editing. (B) Images showing RA-dependent *lacZ* activity in *Aldh1a2$^{+/+}$* (control) and *Aldh1a2$^{-/-}$ Rarb2-lacZ* reporter cells following treatment with 1 µM all-trans retinoic acid (RA) for 16 h. Scale bars: 100 µm.

Development • Supplementary information

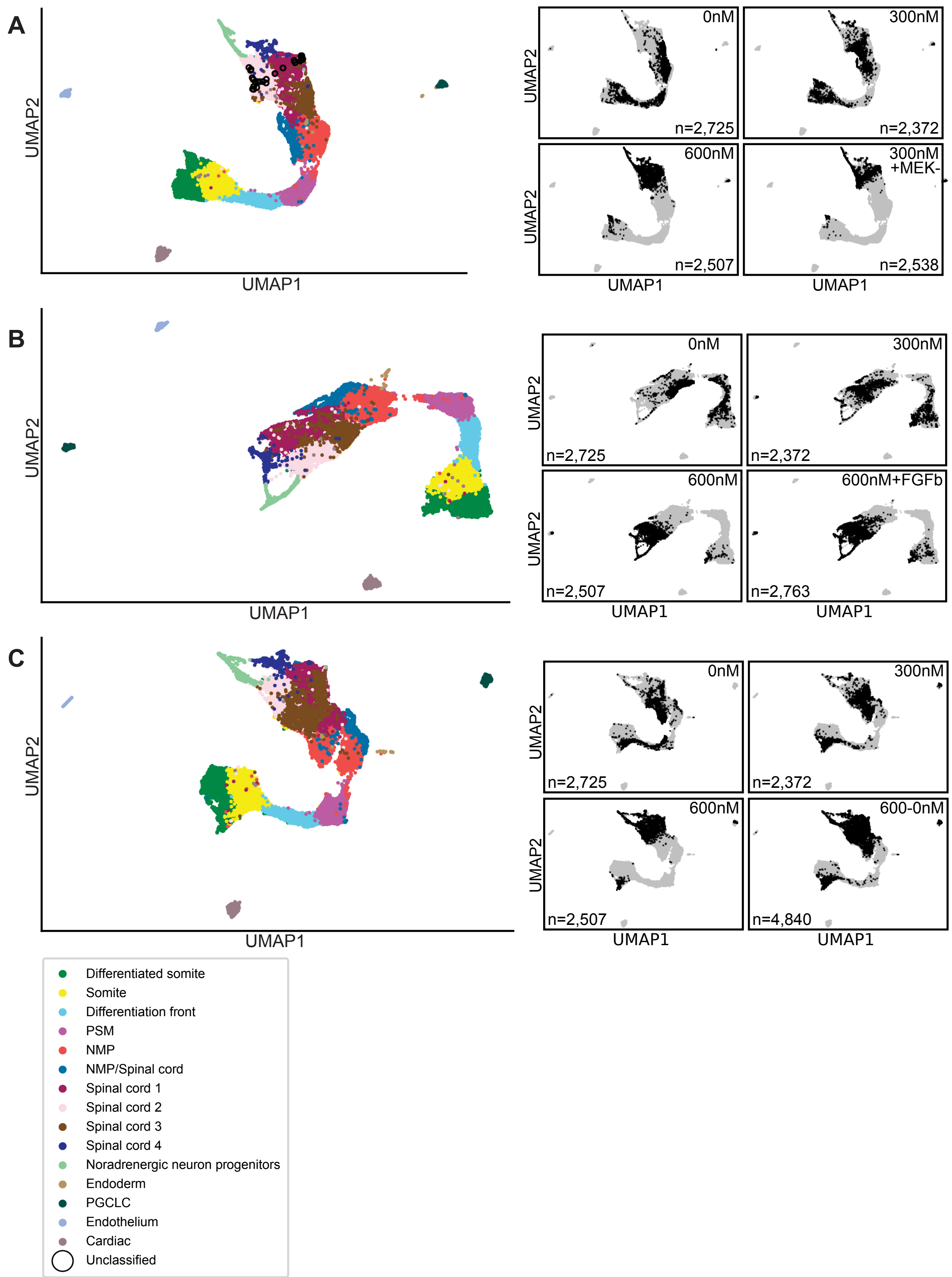


**Fig. S7. Effect of modulation of FGF signaling and retinol concentration on cellular composition.**

(A) (left) UMAP of the integrated scRNA-seq datasets from this study, Brink et al., and the MEK inhibition condition, with cells colored by cell type identity. (right) UMAPs showing the individual datasets and the effect of MEK inhibition (as shown in Fig. 6A) on the integrated dataset. In the corresponding panels, cells from the indicated dataset are highlighted in black, while all other cells are shown in grey. (B) (left) UMAP of the integrated scRNA-seq datasets from this study, Brink et al., and the FGFb treatment condition (as shown in Fig. 6D), with cells colored by cell type identity. (right) UMAPs showing the individual datasets and the effect of FGFb treatment on the integrated dataset. In the corresponding panels, cells from the indicated dataset are highlighted in black, while all other cells are shown in grey. (C) (left) UMAP of the integrated scRNA-seq datasets from this study, Brink et al., and the retinol removal (as in Suppl. Fig9A), with cells colored by cell type identity. (right) UMAPs showing the individual datasets and the effect of retinol removal on the integrated dataset. In the corresponding panels, cells from the indicated dataset are highlighted in black, while all other cells are shown in grey.

Development • Supplementary information

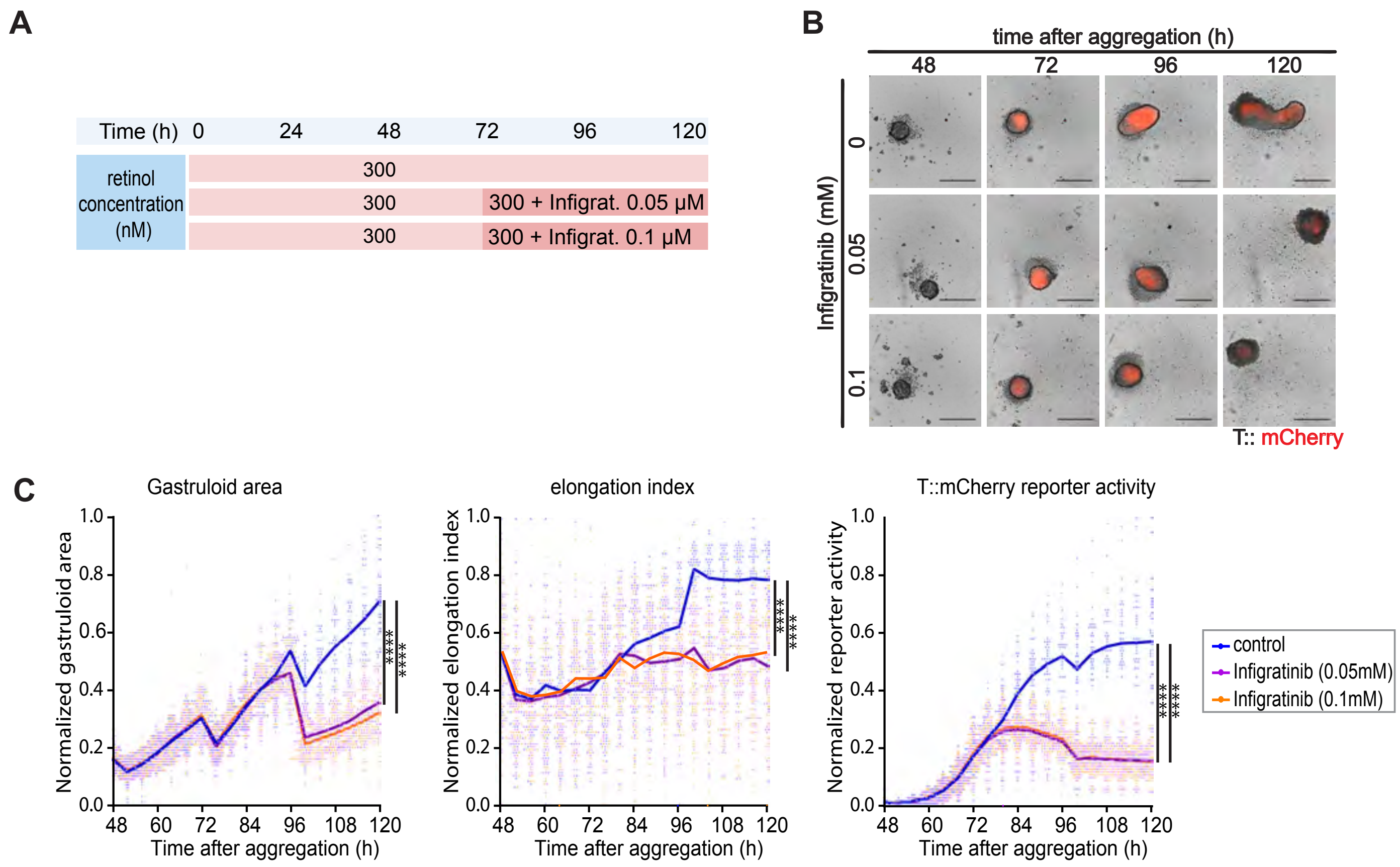


**Fig. S8. Effect of FGF receptor inhibition on gastruloid elongation and T::mCherry reporter activity in gastruloids treated with 300 nM retinol.**

(A) Experimental setup to test dose-dependent effects of FGF receptor inhibitor Infigratinip (0.05uM and 0.1 uM) on gastruloid area, elongation and T::mCherry reporter activity. (B) Representative images of gastruloids treated as in (A). (Red: T::mCherry; scale bars: 400 µm) (C) Graphs showing the normalized gastruloid area, normalized elongation index and normalized mCherry-reporter activity. (Each dot represents a gastruloid. n=35 (control) , n=55 (0.05 µM Infigratinib) and n=58 (0.1 µM Infigratinib) respectively and N=2 biological replicates. Solid lines show the mean. Experimental groups were compared with Mixed-effects analysis with Geisser-Greenhouse correction. Statistics are shown only for the last time point ****p < 0.0001).

Development • Supplementary information

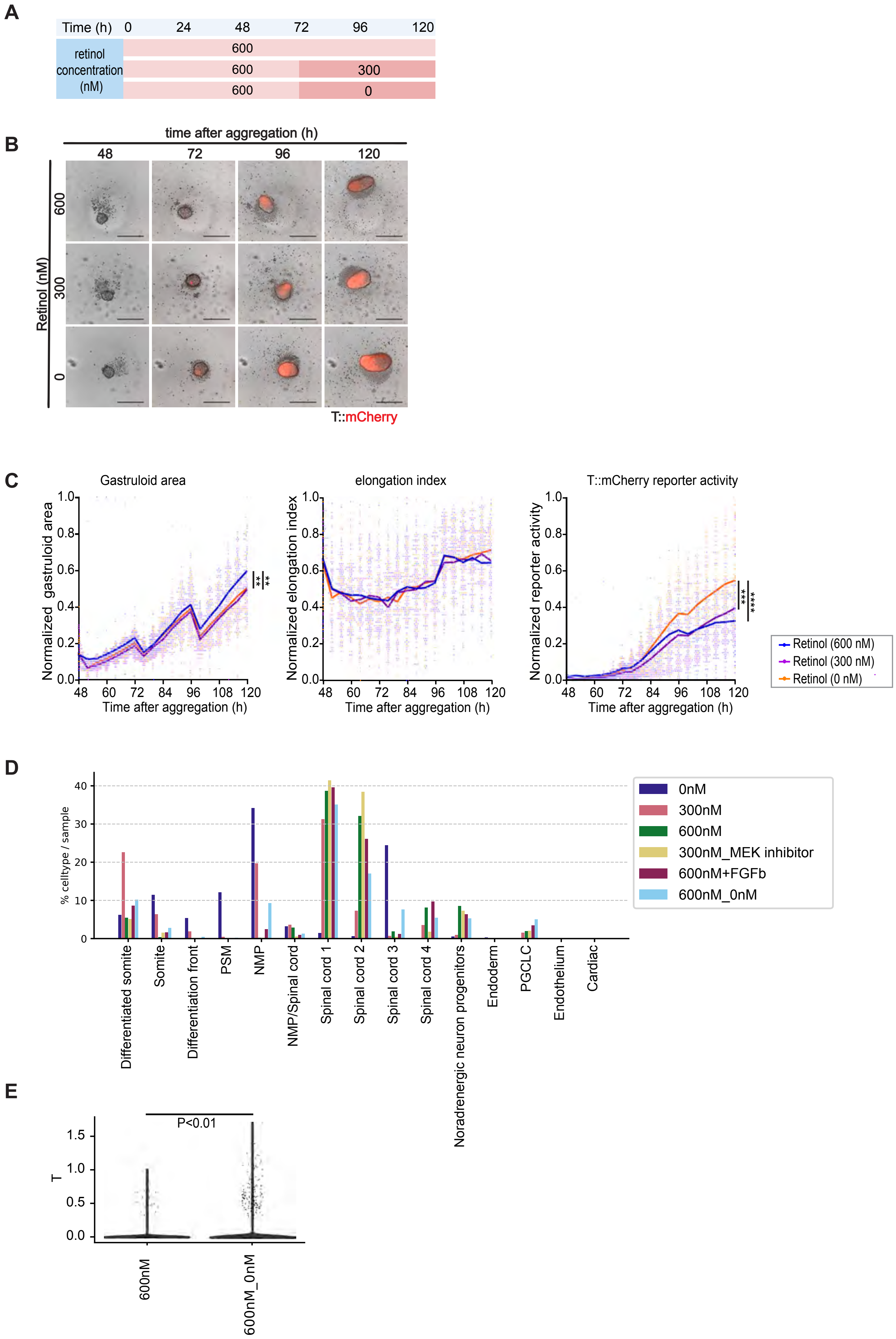


Development • Supplementary information

**Fig. S9. Effect of different doses of retinol on gastruloid elongation and T::mCherry reporter activity.**

(A) Experimental design to analyze the effect of modulating retinol levels during gastruloid generation on their area, elongation and mesoderm formation. Gastruloids were grown in 600 nM until 72 h after aggregation, and subsequently in 0 nM, 300 nM and 600 nM retinol. (B) Representative images of the gastruloids generated from T::mCherry reporter. (Red: T::mCherry; scale bars: 400 µm) (C) Graphs showing the normalized gastruloid area, the normalized elongation index, and the normalized T:mCherry integrated intensity. (Each dot represents a gastruloid. n=52 (600 nM retinol) , n=53 (300 nM retinol) and n=55 (0 nM retinol) respectively and N=2 biological replicates. Experimental groups were compared with Mixed-effects analysis with Geisser-Greenhouse correction. Solid lines show the mean. Statistics are shown only for the last time point, $**p < 0.01$, $***p < 0.001$, $****p < 0.0001$) (D) Bar plot showing the percentage of each cell types in gastruloids treated with various doses of retinol, Mek inhibitor, FGFb or 0 nM retinol. (E) Violin plot showing the distribution of T expression in all cells within gastruloids treated with 0 nM retinol and its corresponding control (600 nM retinol) for the last 48 h of culture ( P-value is the Benjamini-Hochberg corrected P-value resulting from differential gene expression analysis between samples, computed applying the Wilcoxon rank-sum test. ($P < 0.001$))

**Table S1. The dynamic effect of retinol in gene expression during gastruloid culture.** This table includes the list of genes significantly contributing to PC1 or PC2 with the corresponding sign and cluster assignation.

Available for download at
https://journals.biologists.com/dev/article-lookup/doi/10.1242/dev.205153#supplementary-data

**Table S2. Differentially expressed genes (DEGs) across retinol concentrations and time points (24 h and 120 h) after aggregation.** The table lists DEGs identified across retinol conditions and developmental time points.

Available for download at
https://journals.biologists.com/dev/article-lookup/doi/10.1242/dev.205153#supplementary-data

**Table S3. Statistics for Incucyte S3.** This table contains the statistics for image analysis of the images obtained from Incucyte S3.

Available for download at
https://journals.biologists.com/dev/article-lookup/doi/10.1242/dev.205153#supplementary-data

Development • Supplementary information

**Table S4. Differentially expressed marker genes for reference gastruloid cell clusters.** List of marker genes for the different cell type clusters identified in the reference gastruloid scRNA-seq dataset (van den Brink et al., 2020b). For each gene, the table provides the log2 fold change, p-value, adjusted p-value and cluster assignment score.

Available for download at
https://journals.biologists.com/dev/article-lookup/doi/10.1242/dev.205153#supplementary-data

**Table S5. Gene Ontology (GO) enrichment analysis of gastruloid cell types.** This table identifies the biological processes significantly enriched among the differentially expressed genes. Results include the p-value, adjusted p-value, and odds ratio for each term, as well as the specific genes contributing to the enrichment.

Available for download at
https://journals.biologists.com/dev/article-lookup/doi/10.1242/dev.205153#supplementary-data

**Table S6. Differentially expressed marker genes from the reference mouse embryo atlas.** List of marker genes for the embryonic cell type clusters identified in the mouse embryo atlas (Pijuan-Sala et al., 2019). For each gene, the table provides the Ensembl ID, gene symbol, log2 fold change, p-value, adjusted p-value, and cluster assignment score.

Available for download at
https://journals.biologists.com/dev/article-lookup/doi/10.1242/dev.205153#supplementary-data

**Table S7. Gene Ontology (GO) enrichment analysis of embryo-atlas derived cell populations. Significant biological process GO terms associated with the marker genes identified from the Pijuan-Sala et al. (2019) reference.** This table identifies the biological processes significantly enriched among the differentially expressed genes. Results include the p-value, adjusted p-value, and odds ratio for each term, as well as the specific genes contributing to the enrichment.

Available for download at
https://journals.biologists.com/dev/article-lookup/doi/10.1242/dev.205153#supplementary-data

Development • Supplementary information

**Table S8. Antibody list.** This table contains the information of primary and secondary antibodies used in this study

| Antibody | Dilution | Source | Catalog number |
|---|---|---|---|
| Anti-mNeonGreen antibody | 1:200 | Abcam | ab321887 |
| Meox1 Mouse Monoclonal Antibody | 1:400 | OriGene | TA804830 |
| Human/Mouse Brachyury Antibody | 1:100 | R&D systems | AF2085 |
| Human TBX6 Antibody | 1:200 | R&D Systems | AF4744-SP |
| Phospho-p44/42 MAPK (Erk1/2) (Thr202/Tyr204) Antibody | 1:200 | Cell signaling | 9101S |
| Donkey anti-Rabbit IgG (H+L) Highly Cross-Adsorbed Secondary Antibody, Alexa Fluor™ 488 | 1:500 | Life Technologies | A21206 |
| Donkey anti-Mouse IgG (H+L) Highly Cross-Adsorbed Secondary Antibody, Alexa Fluor™ 647 | 1:500 | Life Technologies | A31571 |
| Donkey anti-Goat IgG (H+L) Cross-Adsorbed Secondary Antibody, Alexa Fluor™ 568 | 1:500 | Life Technologies | A11057 |
| Goat Anti-Rabbit IgG H&L (HRP) | 1:500 | Abcam | ab6721 |
| Donkey anti-Goat IgG (HRP) | 1:500 | Santa Cruz Biotechnology Inc | 2056 |

Development • Supplementary information
