# Supplementary material for: Retinoic acid and FGF signaling interact to control elongation and lineage specification in a mouse gastruloid model
Source: Development. 2026 Aug 13;153(15):dev205153. doi: 10.1242/dev.205153 (PMC13535096; doi:10.1242/dev.205153)
Supplement: Supplementary file 2 [file develop-153-205153_review_history.pdf]

Development

Retinoic acid and FGF signaling interact to control elongation and lineage specification in a mouse gastruloid model

Mehmet Yildiz, Noëlle Dommann, Tugce Kardelen Hasdemir, Anna Alemany, Pascale Dijkers, Esther De Vries and Niels Geijsen
DOI: 10.1242/dev.205153

Editor: Matthias Lutolf

Review timeline

Original submission:	7 August 2025
Editorial decision:	14 November 2025
First revision received:	12 May 2026
Accepted:	30 June 2026

Original submission

First decision letter

MS ID#: dev.205153

MS TITLE: Retinoic acid and FGF signaling interact to control elongation and lineage specification in a mouse gastruloid model.

AUTHORS: Mehmet Yildiz; Noëlle Dommann; Tugce Kardelen Hasdemir; Anna Alemany; Pascale Dijkers; Esther De Vries; Niels Geijsen

Dear Dr Geijsen,

I have now received all the referees' reports on the above manuscript, and have reached a decision. The referees' comments are appended below.

As you will see, the referees express considerable interest in your work, but have some significant criticisms and recommend a substantial revision of your manuscript before we can consider publication. If you are able to revise the manuscript along the lines suggested, which may involve further experiments, I will be happy receive a revised version of the manuscript. Your revised paper will be re-reviewed by one or more of the original referees, and acceptance of your manuscript will depend on your addressing satisfactorily the reviewers' major concerns. Please also note that Development will normally permit only one round of major revision. If it would be helpful, you are welcome to contact us to discuss your revision in greater detail. Please send us a point-by-point response indicating your plans for addressing the referees' comments, and we will look over this and provide further guidance.

Please attend to all of the reviewers' comments and ensure that you upload both a 'clean' version of your Word file, along with a highlighted version clearly showing where you have made changes in the revised manuscript. Please avoid using 'Tracked changes' in Word files as these are lost in PDF conversion. I should be grateful if you would also provide a point-by-point response detailing how you

have dealt with the points raised by the reviewers in the 'Response to Reviewers' box. If you do not agree with any of their criticisms or suggestions please explain clearly why this is so.

Reviewer 1

Yildiz and colleagues present their research on Retinoic Acid (RA) and FGF signalling in murine gastruloids. The work represents an interesting research question with some important implications, although it does make some claims that I find to be unsubstantiated. It would require substantial revision to provide the supporting data necessary to uphold their statements or these would need to be toned down in the text, alongside several improvements for the strength of the data, including quantifications. I have outlined some suggestions below.

Major Comments

- The abstract makes several claims that I do not see reflected in the results. For instance, "Here we examined [RA]s role on axial elongation…" when they actually looked at the role of Retinol; "RA was successfully metabolized prompting…" I have seen no metabolism data in the manuscript; "…the appearance of…" makes it sound like the endoderm and PGC populations aren't present in the absence of Retinol, which isn't true; "…through interaction with FGF signaling" there is no direct interaction data; "Our novel model…" the cellular (gastruloid) model itself is not new, and while modulating the conditions are interesting, that does not mean that a new model has been made - if the authors mean a conceptual model, then this is also not new, as FGF/RA interplay has been well-described in embryos before.
- Throughout the text, the authors refer to RA or FGF 'gradients' but I see no evidence of this. There may be some anterior or posterior localisation of RA-response and SPRY4, but this is certainly not the same thing as a gradient (for which one would need to show a continuum of levels across space) or even necessarily a signalling polarisation (for which one would prefer to have the downstream cascade eg pERK or more than one gene product). This needs to be revised in the text, or further experiments added.
- I find the section from 151-168 poorly reasoned and difficult to understand. They say "we plotted the average expression levels of all differentially upregulated genes for each cluster across all timepoints and retinol concentration conditions" but how are these genes selected and analysed - are the differentially expressed between clusters or timepoints? Are they averaged within clusters or across them? Have they grouped together all timepoints and all retinol concentrations? I couldn't find this information in the methods. Depending on the answers to these questions, it seems they are likely to be conflating the differences in cell type proportions with gene expression identity. I also don't buy the argument on Line 156-7 about efficiency and think it is more likely that although the cells are labelled as 'tailbud' they may have an altered gene expression identity, and this therefore has more to do with the processing (ie integration of datasets) or clustering than the biological meaning.
- The authors make the claim that within certain clusters, some cells express Fgf8 while others express Aldh1a2 and this leads them to suggest that there are subpopulations. This would be interesting if true, but needs more substantiation. Given the known drop-out rate of single cell transcriptomics, they would need to show that the exclusivity occurs more frequently than would be expected by chance with the given expression levels. It would also help to show that the two populations sub-cluster using an unbiased approach for instance in UMAP space across multiple genes.
- I don't know what a 'trunk progenitor' is if not an NMP. The authors should clarify their cell type assignments, and ideally refer back to the embryo by label transfer to clarify these identities.
- They speculate that these cells "may no longer fully metabolise RA" but this is entirely unfounded given they are looking at transcriptomic data. There is also no evidence for their spatial localisation (lines 203-206). Both claims need to be removed.
- Additional statistical and quantitative support would be beneficial throughout the text. For example, when using representative images (Line 213) they could add n numbers of observed vs examined (as done on ISH staining of gastruloids in Beccari et al 2018). They should also perform statistical tests in order to say that b-galactosidas increased in a dose-dependent manner (Line 215).

It would also be good to include an AP axis quantification if they want to make claims about the polarisation of signals, as has been done in several other gastruloid papers.

- Other papers have looked at the role of FGF on gastruloid development, so these papers need to be cited and the observations here made in relation to existing knowledge.
- I find the section on phenocopying/rescuing to be overstated. It is hard from lack of elongation alone to be able to conclude much about interactions between signals, let alone about cell type composition or mechanism etc. This section needs to be toned down and ideally additional experiments added. For instance, I don't think the authors can claim that mesoderm formation is affected purely by looking a brachyury levels (which is transient and doesn't mark mature mesoderm). I also think the correlation between two effects doesn't go far enough to allow causation to be inferred. I think this section is possibly the weakest part of the manuscript at the moment.
- The authors don't seem to see endoderm in their gastruloids. This should be discussed in the text as the presence of 3 germ layers is thought to be the defining feature of a gastruloid.

Minor Comments

- I think the authors misrepresent the contextual developmental biology in their introduction. The claim that "Despite all the accumulated knowledge on FGF and RA signaling, the physiological effects of cross-talk between the two pathways in key processes such as gastrulation remain unclear". There is a well-known relationship between FGF and RA and the authors cite many of the papers on these topics. I don't think they need to (or should) claim that little is known just to provide grounding for their research study.
- The authors should be careful to explain what is new in what they have done, and what has been shown before. For example, "Here we set up an in vitro model… using gastruloids…" makes it sound like they have devised a new protocol/system rather than made use of an existing model.
- The authors never explain why they use Retinol rather than Retinal.
- Lines 83-86 need to refer to data in the figures to substantiate claims.
- Line 90 should be 'time course' not 'time lapse'
- Line 138 would be good to clarify to the reader what the Rarb1 line is and what it can be used for.
- I find the phrasing in Line 189-190 confusing. Do they mean these genes aren't expressed in the clusters that are retinol-biased?
- The authors claim (Line 231) that there are opposing FGF/RA gradients, but I didn't see any co-staining in the same structure, or with a universal marker like T/bra. This should be revised.
- The claim of decreased FGF target gene expression (Line 233) should be toned down given they have only looked at 1 gene

Figure Comments

- The schematic in 1A implies that the gastruloids are so big as to curl with the edge of the U-bottom plates. This is not accurate and should be revised.
- 1B it would have been good, here and throughout, to have a standard gastruloid (ie N2B27+VitA) as a positive control
- Typo in 1C 'length'
- 3B show individual data points, including differences between experimental runs
- It wasn't clear to me at first glance that Clusters 0/1 on Fig 2B were related to PC1 and the other to PC2. Could be clarified on the figure itself.
- 4D would be helpful to show ISH on a white background given that it is a dark stain
- 5A/5D it isn't clear if retinol is maintained while the FGF treatments are added. Clarify in text and figures as this will affect which control is appropriate.
- The 'dips' in morphometric measurements, eg 5C or 5F, seem to correlate with media changes. Does this mean it is measuring cell debris more than the edges of the gastruloids? If so, the method should be revised as it is not accurately conveying gastruloid morphology.
- 6C I see no evidence of a gradient from the data shown.

Reviewer 2

SUMMARY OF THE ADVANCE MADE IN THIS PAPER AND ITS POTENTIAL SIGNIFICANCE TO THE FIELD In this study by Dr. Geijsen and colleagues, the authors assesses the effect of retinol (retinoid signalling) on the morphology and the cell types found in mouse gastruloids, which are used as an in vitro surrogate to study the formation of the embryonic A-P axis. By adding various concentrations of retinol, the levels of endogenous retinoic acid activity are altered/controlled and the resulting gastruloids are examined using bulk RNA-seq, scRNA-seq and reporter lines. The interactions between FGF- and retinoid activity are also examined. This study shows that gastruloids can provide valuable insights into aspects of mouse early development. The presented data largely supports the conclusions of the study, however there is room for improvements.

SUGGESTIONS TO AUTHORS

Line 80-86: This paragraph seems to suggest that excessive retinol gives rise to suboptimal gastruloid morphology, using elongation as the primary criterion. The authors should define the morphological features of 'optimal gastruloids' to begin with. This would especially help the readers unfamiliar with the model system. Including characterization of the major cell types in gastruloids (e.g. immunofluorescence images) and clarification of which are lacking at the extreme ends of the retinol concentration range would furthermore better link morphology to cell type development.

Fig 1C: Typo: Length

Line 87-114: The principal component analysis is a bit obtuse and its value unclear. Please improve the overall clarity of this section.

Line 102-114: Listing possible associated cell types for each cluster does not seem to be the most efficient way to interpret the variance along PC2, particularly since co-expression information for these genes is not available. In figure 2A, samples from different retinol concentrations are already separated along PC2 at 0 hours and the difference increases with time. This pattern and the associated genes are not well described in this paragraph. Are the genes driving this variance at 0 hours similar to those at later time points? Could the authors provide example gene names?

Line 120-121: Why wasn't the dual reporter mentioned in Line 131 chosen? One gastruloid - same information, less biological noise between varying morphologies and an almost direct comparison within the same sample. Could the authors kindly explain and elaborate.

Line 120-121: Can the same line be used for both the time-lapse and the FACS experiment? It would make the comparison between the results easier, especially since it was mentioned that each cell line behaves slightly different.

Line 122, Figure 2D and 2E: In the graphs there are noticeable troughs at certain time points (for T::mCherry, at 48h, 72h, 96h). Are these reflecting technical artifacts, or represent a biological effect?

According to Figure 2C, T expression seems to reach a peak at 72 hours and decreases, at the transcriptional level. In Figure 2D, reporter activity, which should correlate with the transcriptional activity of T, overall increases over time. The authors should address and explain this discrepancy. Furthermore, the T::mCherry signal seems to have high variability (localization of replicate values). Adding confidence intervals may be useful for visualizing the difference in signal intensity relative to the variability.

Figure 2D: There seems to be massive cell death even at 96h and 120h? Could the authors please explain why that may be and what alternatively the 'halo' around each gastruloid is? The Sox2 line doesn't look as bad btw. Interestingly the 300nmol retinol condition has increased T activity compared to the 0nmol. No statement on whether the intensity difference between 300nmol and

0nmol at 120h is significant on the graph or not but it looks visibly significant (maybe they just swapped a column by accident; it can also be a matter of normalizing signal by gastruloid area as it seems visibly correct at 120 on the representative images just not in the graph).

Line 132, Figure 3B: Figure 2D, which measured the T::mCherry signal shows that the reporter activity is present at 120hrs even at the highest concentration, although at a lower level. The percentage of mCherry-positive cells, measured by FACS, appears equal to or very close to 0. The authors should explain the discrepancy or provide additional data.

Line 133-134: The authors claim that the FACS results are in line with their time lapse data but Figure 3B for T count is contradicting with what can be seen in the Graph for Figure 2D. The jump of higher T intensity from 0 to 300 is opposite of the count seen in FACS sorting. If they were to repeat the time lapse experiment with the double reporter would they get the same result? They could also do FACS again with one set of SOX2 only reporter gastruloids and a set of T only reporter.

Line 138: Why did the authors not use a wild-type line but another reporter?

Figure 3E: What are the trunk progenitors that seem to only be present at 300nM? In supplemental figure 3C they have a similar expression level as 600nM, but the cells are not present in the 600nM cell population in Figure 3E.

Figure 3G: In addition to Hes7 which is an oscillating gene, the authors should consider using e.g. TBX6 or MSGN1 as Paraxial Mesoderm Markers.

Line 151-153, Figure 3F-I: Retinol 600nM and 1200nM seem to have some cell types in common. Have the authors examined within the same cell type cluster gene expression differences across retinol concentrations? It may also be informative to visualize the time-course expression trends of genes involved in RA synthesis across different retinol concentrations and see in parallel with the ones for FGF8.

Figure 4B &; C: Which gastruloids were used to produce this data? All the different conditions at 120h?

Figure 5: Gastruloid Surface, Elongation Index is never defined. Colors bleed into each other on the graphs making it hard to read.

Figure 5B &; E: Scale bar info is missing.

Figure 6B, D: I don't see how they can make these claims from the data they've shown and discussed. The authors should provide more references and other data sets e.g. from mouse embryo data to back up their proposed model. How good does the single cell data of their in vitro model cluster with the in vivo data?

Considering how in Figure 6, the authors are making comparisons with mouse embryos, it would have been nice to see some staining of representative genes of members of the different cell populations described in Figure 3 and 6D, either by antibody staining or in-situ hybridization.

Line 452: Formatting: 0,1 decimal comma. In English usually a decimal dot is used

Supplemental Figure 4 &; 5: Scale bar info is missing. In general, scale bars are in many cases very difficult to see, likely due to the small image sizes.

First revision

Author response to reviewers' comments

RESPONSE TO REVIEWERS

We would like to thank the reviewers for their constructive feedback and insightful comments, which have significantly helped improve the quality and mechanistic depth of our manuscript.

Please find below our detailed point-by-point responses to the comments and questions raised by the reviewers. Reviewer comments are highlighted in blue and bold, followed by our detailed responses. References to figures and tables correspond to the revised version of the manuscript unless stated otherwise. New figures and equations specific to this response document are labeled with an "R" (e.g., Figure R1).

Reviewer 1

1) The abstract makes several claims that I do not see reflected in the results. For instance, "Here we examined [RA]s role on axial elongation…" when they actually looked at the role of Retinol; "RA was successfully metabolized prompting…" I have seen no metabolism data in the manuscript; "…the appearance of…" makes it sound like the endoderm and PGC populations aren't present in the absence of Retinol, which isn't true; "…through interaction with FGF signaling" there is no direct interaction data; "Our novel model…" the cellular (gastruloid) model itself is not new, and while modulating the conditions are interesting, that does not mean that a new model has been made - if the authors mean a conceptual model, then this is also not new, as FGF/RA interplay has been well- described in embryos before.
We thank the reviewer for these insightful comments. We agree that several claims in the abstract require further clarification and more precise terminology. We have revised the resubmitted version of the manuscript as follows to address each of the points raised by the reviewer:

- **"they actually look at the role of Retinol"**

We thank the reviewer for catching this oversight regarding the source of Retinoic Acid (RA). The manuscript's abstract now specifies that we examined the role of the RA precursor, retinol, on axial elongation: *"Here, we examined the role of a RA precursor, retinol, on axial elongation…"*.

- **"I have seen no metabolism data in the manuscript"**

To address the concern regarding metabolism data, we have removed any claims regarding to RA metabolism in the abstract. Nevertheless, we still want to clarify what we originally meant with this statement since it impacts how we interpret our experimental outcomes. The LacZ reporter cell line used in our study was taking from S. Shen et al (PMID: 1338286). In this line, beta- galactosidase (β-gal) expression is under control of a mRARbeta2 promoter, which is only active in the presence of RA in the cells. Since our medium is RA-free (as shown in Fig. 5C), the expression of β-gal in the cell line indicates that retinol is successfully metabolized to RA. In our resubmission, we included the following sketch of the cell line (Fig. R1.1) to Fig. 5C to explain how this cell line allows us to report retinoic acid metabolism:

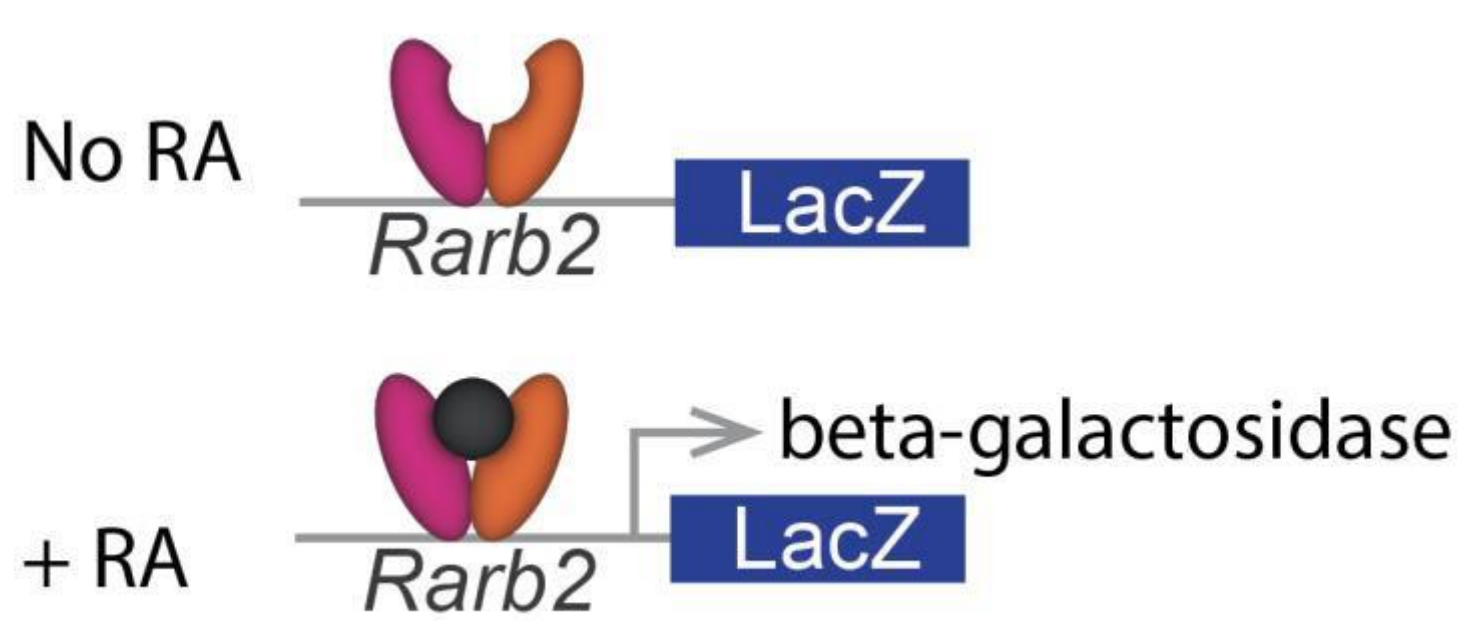

Figure R1.1. Schematic representation of the Rarb2.LacZ reporter mESC cell line. Beta-galactosidase (β-gal) is encoded by the LacZ gene, which is under the mRARbeta2 (Rarb2) promoter. Presence of Retinoic Acid (RA) in the cells results in the expression of β-gal.

To further validate that the presence of β-gal in this cell line is driven by the conversion of Retinol to RA, we generated an Aldh1a2 -/- Rarb2.LacZ knockout cell line. While Aldh1a2 +/+ gastruloids showed robust LacZ expression 120h after aggregation when treated with 300nM retinol (57/63), expression was absent in the Aldh1a2 -/- gastruloids (0/59). These results, shown in Fig. R1.2 and included in Fig. 5C in the resubmitted manuscript, demonstrated that the observed LacZ expression is driven by the metabolic conversion of retinol into RA.

- "endoderm and PGC populations aren't present in the absence of Retinol"

We thank the reviewer for catching this oversight. As the reviewer will see while addressing future points, the single-cell RNA sequencing data has been replaced by a new dataset since we realized that we could not replicate the drastic effects that retinol concentration was having on the PGCLC cluster (we have never seen gastruloids grown at 300 nM with more than 10% PGCLC). Accordingly, in the new abstract, we removed any reference to PGCLC and focus on the effect that retinol concentration has on the ectoderm and the mesoderm populations, and replaced "promoting the appearance" by "showed an enrichment". Finally, we assume the reviewer made a typo here because we did not (and still do not) mention the endoderm in the previous abstract.

- "there is no interaction data" and "novel model"

As the reviewer points out, we do not have data showing a direct "interaction" between RA and FGF. We have therefore moderated the concluding claims. We removed the reference to a "direct interaction" with FGF and the description of the gastruloid system as a "novel model." The final sentences of the abstract now read: "Thus, retinol can modulate cellular composition in gastruloids, allowing us to investigate fate commitment of ectoderm and mesoderm progenitors *in vitro*."

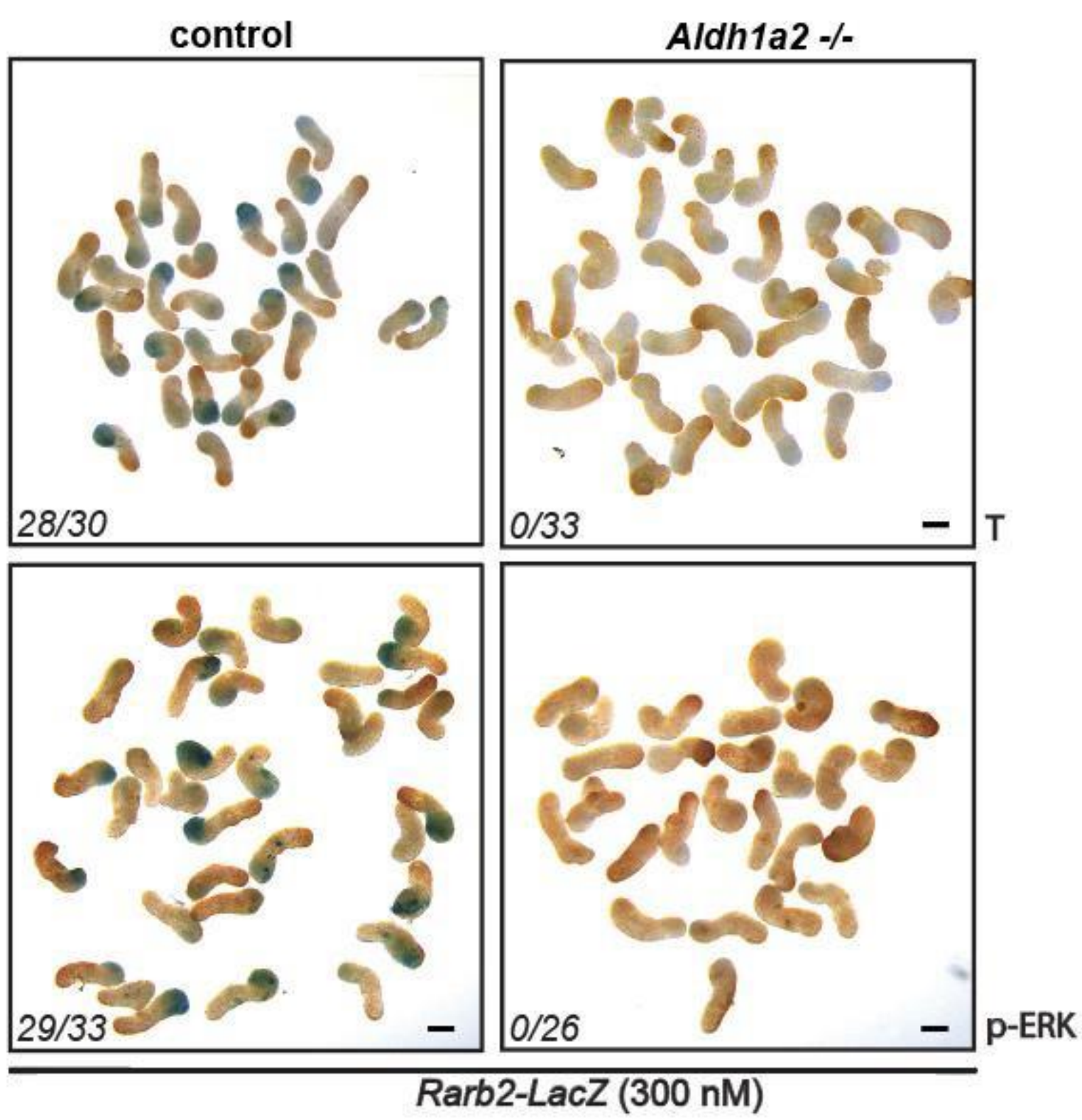

Figure R1.2. Brightfield images of Aldh1a2 +/+ and Aldh1a2 -/- 5 gastruloids cultured in 300 nM retinol 120 h after aggregation. LacZ and T (Brachyury) co-staining: Wild type (Aldh1a2+/+,top left) and knockout (Aldh1a2−/−, top right) gastruloids. RA-driven LacZ expression is indicated by blue (X-gal), and T expression by brown (HRP**)**. LacZ and pERK costaining: Wild type (Aldh1a2+/+, bottom left) and knockout (Aldh1a2−/−, bottom right) gastruloids. LacZ expression (blue, X-gal) is shown alongside the pERK gradient (brown, HRP). Scale bars: 200 µm.

2) Throughout the text, the authors refer to RA or FGF 'gradients' but I see no evidence of this. There may be some anterior or posterior localization of RA-response and SPRY4, but this is certainly not the same thing as a gradient (for which one would need to show a continuum of levels across space) or even necessarily a signalling polarization (for which one would prefer to have the downstream cascade, eg pERK or more than one gene product). This needs to be revised in the text, or further experiments added.

We agree with the reviewer that our data was not explicitly showing gradients and replace these statements overall as "signalling polarization" as suggested. On top of that, to strengthen our claims about signalling polarization, we performed staining for T (as a posterior marker), pERK (as an FGF signalling readout, upon referee suggestion), and RA-driven LacZ expression in gastruloids cultured with 300nM retinol (Fig. R1.2). Our new results showed anterior localization of LacZ staining, while T and pERK stainings were both observed at the posterior side. These distributions indicate clear polarization of pERK signaling and RA activity within the gastruloids. These results have been included in the resubmitted manuscript in Fig. 5C.

To further explore the localization pattern of pERK and T expression in gastruloids as a function of retinol, we co-stained pERK and T in 120h old gastruloids cultured at 0 nM, 300 nM, 600 nM and 1200 nM. As the reviewer can see in Fig. R1.3, these data showed that both posterior marker T and posterior pERK expressions were downregulated with retinol. At higher dose (1200 nM retinol) both pERK and T expression at posterior were downregulated dramatically, resulting in no elongation. These results have been included in the resubmitted manuscript in Fig. 5E.

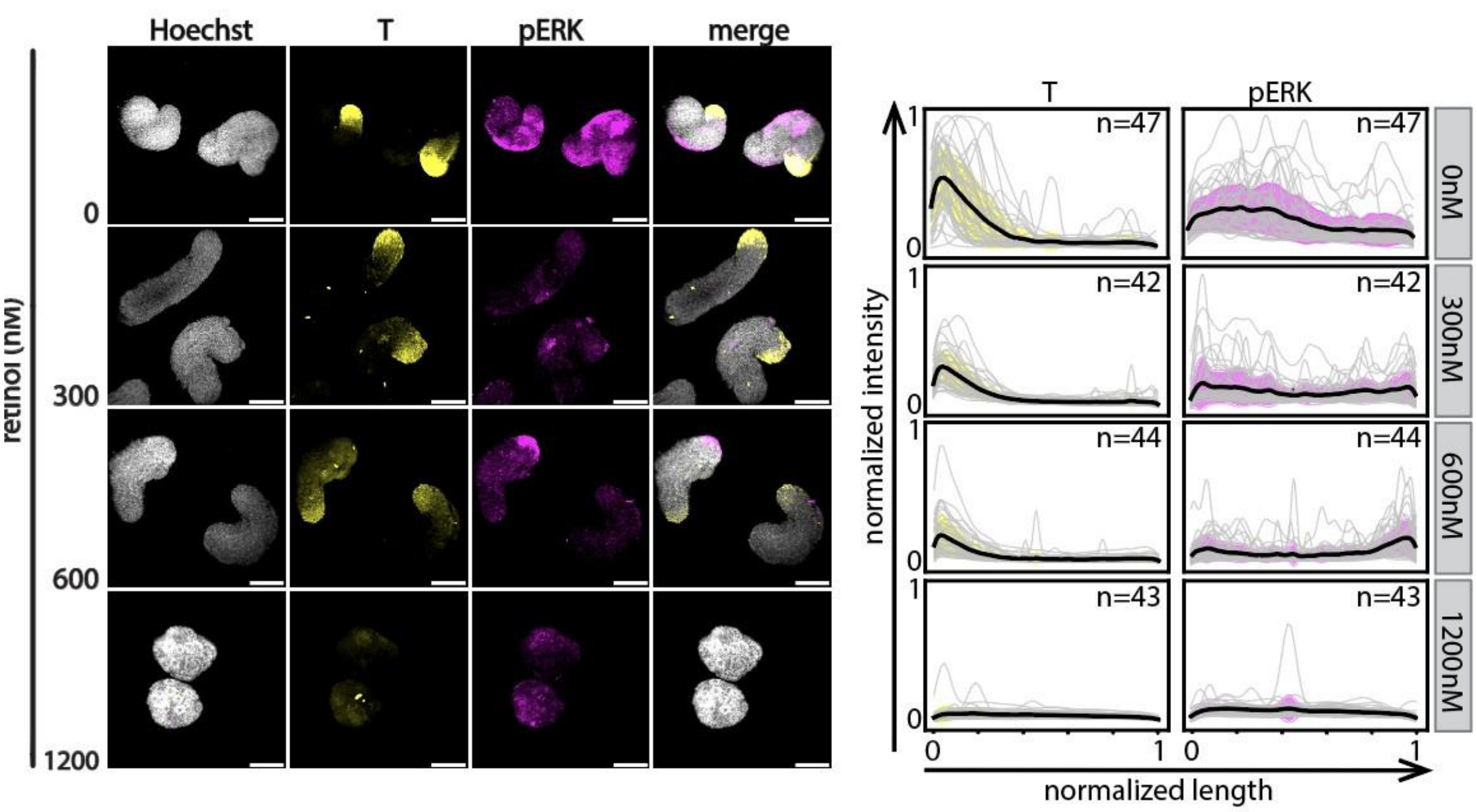

Figure R1.3. FGF signaling localization in gastruloids.
(Left) Co-staining of T and pERK in gastruloids 120 h after aggregation treated with 0 nM, 300 nM, 600 nM and 1200 nM retinol. (Right) Relative intensity of T staining from the posterior end to the anterior end in gastruloids. The black line shows mean intensity across all replicates (indicated in

each panel), and the yellow area indicates the standard error of the mean. Relative intensity of pERK staining from the posterior end to the anterior end in gastruloids. The black line shows mean intensity across all replicates (indicated in each panel), and the purple area indicates the standard error of the mean. Scale bar is 200 µm.

3) I find the section from 151-168 poorly reasoned and difficult to understand. They say "we plotted the average expression levels of all differentially upregulated genes for each cluster across all timepoints and retinol concentration conditions", but how are these genes selected and analysed - are the differentially expressed between clusters or timepoints? Are they averaged within clusters or across them? Have they grouped together all timepoints and all retinol concentrations? I couldn't find this information in the methods. Depending on the answers to these questions, it seems they are likely to be conflating the differences in cell type proportions with gene expression identity. I also don't buy the argument on Line 156-7 about efficiency and think it is more likely that although the cells are labelled as 'tailbud' they may have an altered gene expression identity, and this therefore has more to do with the processing (ie integration of datasets) or clustering than the biological meaning.

Gene selection was based on the marker genes for each cell type identified at day 5 by differential gene expression analysis performed in our scRNA-seq data (Suppl. Table 3). Therefore, identification of markers did not take into consideration whether a cell type was detected in one or more retinol conditions. Nevertheless, we agree with the reviewer that the distribution of some cell types in the UMAP could be attributed to processing rather than biological effects. To prevent this, we generated a new scRNAseq dataset using 10X on-chip-demultiplexing. This strategy allowed us to process all samples simultaneously under the same conditions thereby reducing the risk of batch effects. Since this batch effect-free dataset has replaced the old one and has been analyzed together with previously published scRNAseq data, we are convinced that the effects observed in the resubmitted version of the manuscript are biological and not a result of data processing. We added new single-cell RNA sequencing data in our current manuscript at Fig. 3A.

In our first submitted manuscript, bulk RNA-seq data was subsequently used to visualize expression dynamics of the mean VST computed among all markers in each cell type as a function of time and retinol concentration (previously Fig. 3F-I and Suppl. Fig. 3). To address the reviewer's comment, we decided to replace the line plots of the average expression of marker genes per cell type by a new figure (Fig. R1.4 and Suppl. Fig. 1D) in which we showed selected mesodermal and ectodermal genes in bulk RNA sequencing regardless of looking at the cell clusters.

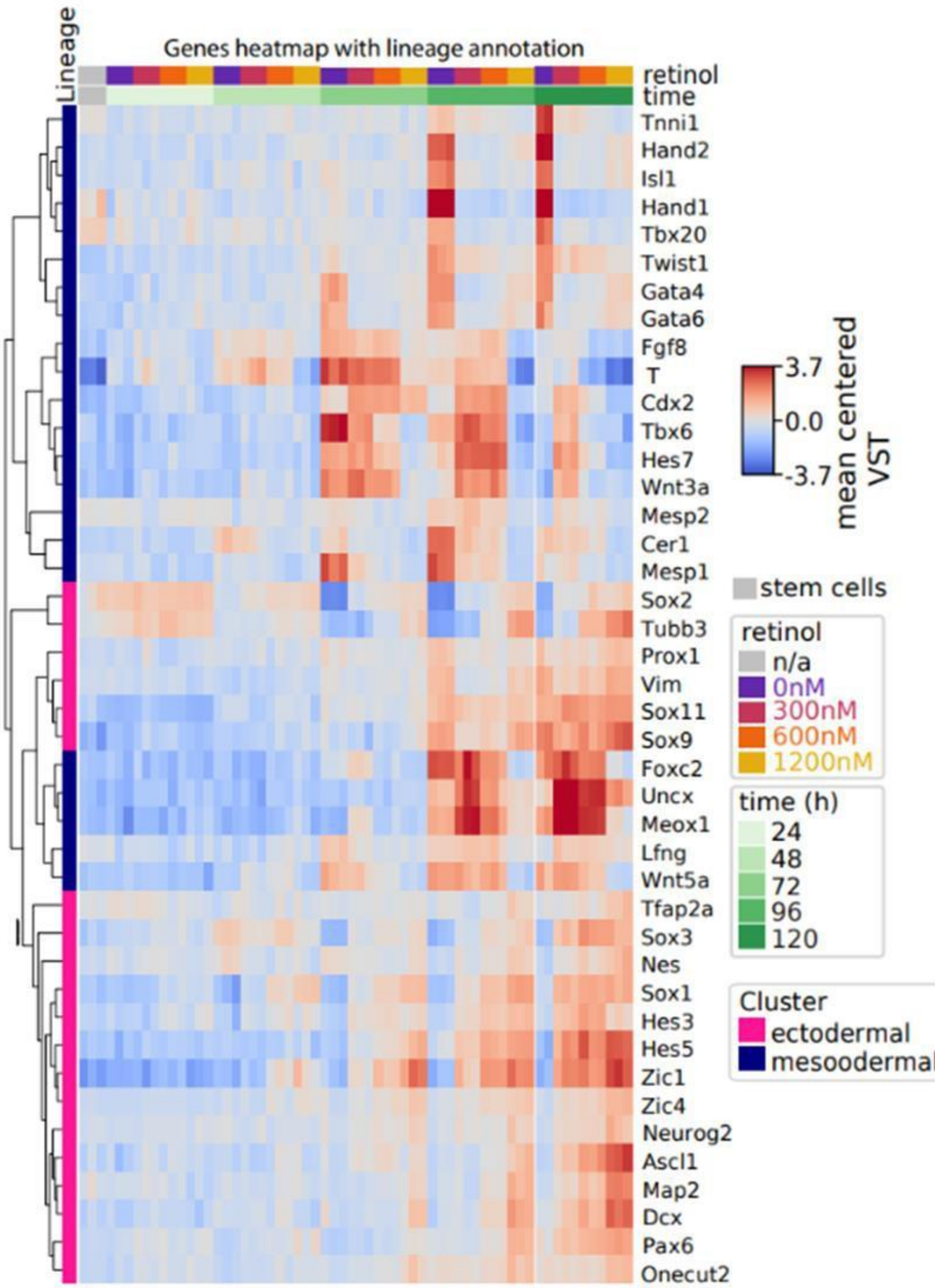

Fig.R1.4. Heatmap showing the time course mean centered variance stability transformed (VST) RNAseq data of selected mesodermal and ectodermal genes.

4) The authors make the claim that within certain clusters, some cells express Fgf8 while others express Aldh1a2 and this leads them to suggest that there are subpopulations. This would be interesting if true, but needs more substantiation. Given the known drop-out rate of single-cell transcriptomics, they would need to show that the exclusivity occurs more frequently than would be expected by chance with the given expression levels. It would also help to show that the two populations sub-cluster using an unbiased approach for instance, in UMAP space across multiple genes.

We thank the reviewer for this observation and agree that the potential for stochastic dropout in single-cell transcriptomics requires a more rigorous and unbiased approach to define cell identities. Accordingly, we removed the corresponding claim and accompanying figure. Instead, we used our

dataset to perform differential gene expression analysis across retinol concentrations, which provided additional biological insights:

- Aldh1a2 was significantly overexpressed in cell types generated under 0 nM retinol conditions compared with those generated in the presence of retinol (Fig. R1.9 and Fig. 5B).
- Our data now clearly demonstrate that retinol promotes the maturation of neural progenitors (Fig.R2.7 and Suppl. Fig. 4D).

5) I don't know what a 'trunk progenitor' is if not an NMP. The authors should clarify their cell type assignments, and ideally refer back to the embryo by label transfer to clarify these identities.

We thank the reviewer for this comment. To clarify and improve the cell type annotations of our single-cell RNA sequencing (scRNA-seq) dataset, we integrated our data with the reference dataset from Brink et al. (PMID: 32076263) (Fig. R1.5A and Suppl. Fig.4A). We complemented this integration with gene ontology analysis and quantification of the shared markers between our clusters and the cell types annotated in the Pijuan-Sala et al. mouse embryo atlas (PMID: 30787436); (Fig. R1.5C and Suppl. Fig. 4C).

As a result, we have updated our nomenclature to align with standard embryonic labels. The "trunk progenitor" population has been re-assigned to the NMP or PSM lineages as appropriate. Our finalized annotations now include:

Differentiated Somites, Somites, Differentiation front, PSM, NMP, NMP/Spinal cord, Spinal cord 1–4, Noradrenergic neuron progenitors, Endoderm, PGCLC, Endothelium, and Cardiac cell types.

These results are detailed in Fig. R1.5 and have been included in the resubmitted manuscript in Fig. 3, Suppl. Fig. 4A-C, and the Methods section.

NOTE: We have removed unpublished data that had been provided for the referees in confidence using data from van den Brink SC, Alemany A, van Batenburg V, Moris N, Blotenburg M, Vivié J, Baillie-Johnson P, Nichols J, Sonnen KF, Martinez Arias A, van Oudenaarden A. Single-cell and spatial transcriptomics reveal somitogenesis in gastruloids. Nature. 2020 Jun;582(7812):405-409. doi: 10.1038/s41586-020-2024-3. Epub 2020 Feb 19. Erratum in: Nature. 2020 Mar;579(7799):E11. doi: 10.1038/s41586-020-2113-3. PMID: 32076263.) and Pijuan-Sala, B., Griffiths, J.A., Guibentif, C. et al. A single-cell molecular map of mouse gastrulation and early organogenesis. Nature 566, 490–495 (2019). https://doi.org/10.1038/s41586-019-0933-9

Fig. R1.5. Label transfer for cell type identification.
A) (left) Resulting UMAP obtained from integrating the scRNAseq dataset generated in our study together with the 10X Chromium dataset from Brink et al, showing cells colored by Leiden cluster identity. (right) Heatmap showing, for each annotated cell type, the fraction of cells in each Leiden cluster. This is restricted to cells from the Brink et al dataset. B) Same UMAP where cells coming from Brink et al or from the different Retinol culture conditions obtained in this study are colored in black, and the rest in gray. C) Dotplot showing common number of markers between the Leiden clusters shown in panel A and cell types annotated in the scRNAseq mouse embryo atlas from Pijuan Sala et al. Red indicates significant overlap (P<0.001), while gray indicates not significance. The size of the dot is proportional to the number of common markers.

6) They speculate that these cells "may no longer fully metabolise RA" but this is entirely unfounded given they are looking at transcriptomic data. There is also no evidence for their spatial localisation (lines 203-206). Both claims need to be removed.

We thank the reviewer for his insight, and we have removed these claims.

7) Additional statistical and quantitative support would be beneficial throughout the text. For example, when using representative images (Line 213) they could add n numbers of observed vs

examined (as done on ISH staining of gastruloids in Beccari et al 2018). They should also perform statistical tests in order to say that b-galactosidas increased in a dose- dependent manner (Line 215). It would also be good to include an AP axis quantification if they want to make claims about the polarisation of signals, as has been done in several other gastruloid papers.
We thank the reviewer for the suggestion to strengthen the quantitative support of our findings. As requested, we have updated the manuscript to include n numbers (observed vs. examined) for all representative Beta-galactosidase (X-gal) staining images (Fig. R1.2 and R1.3, corresponding to Fig. 5C,D in the manuscript). Regarding the dose-dependent increase of Beta-galactosidase mentioned in the original Line 215, we would like to emphasize that enzymatic X-gal staining is primarily a qualitative assay, which precluded robust statistical testing. We have therefore removed the claim that this signal increases in a dose-dependent manner.

Additionally, we have performed image analysis and AP axis quantification for T, pERK, Tbx6, Meox1, and Uncx4.1 stainings to substantiate our claims regarding signal polarization (Fig. R2.1 and Fig. 4C, 5E). We quantified their intensity via FIJI line option from posterior to anterior, which can be seen throughout the paper in Fig.4C and Fig. 5E.

For the analysis of Sox2::Venus, Sox1::GFP, T::mCherry, and Uncx4.1:ngreen (Fig. R2.1 and Fig. 4A,B), we utilized FACS analysis as our primary quantitative tool. We believe FACS provides a more precise and unbiased quantification of cell type percentages than image-based analysis of representative sections. Therefore, the images provided for these markers serve as spatial illustrations to complement FACS quantification.

8) Other papers have looked at the role of FGF on gastruloid development, so these papers need to be cited and the observations here made in relation to existing knowledge.
We thank the reviewer for this comment. In the revised manuscript, we have further contextualized our findings by expanding the Discussion (Lines 180–192) to include a more explicit integration of our results with established models of FGF-driven axial elongation and mesoderm patterning. Explicitely, we now include the following text in section "**RA inhibits FGF signaling, which is necessary for early mesoderm formation and elongation in gastruloids"**:

"These findings are consistent with previous data showing that MEK inhibition results in shortening of gastruloids without markedly impairing neural differentiation (van den Brink et al., 2020b; Van Den Brink et al., 2014). "

This updated synthesis clarifies how our observations regarding the FGF/RA metabolic interplay complement and extend existing knowledge of gastruloid symmetry breaking. By positioning our data alongside these foundational studies, we more clearly define our contribution to the understanding of signaling coordination in these systems.

9) I find the section on phenocopying/rescuing to be overstated. It is hard from lack of elongation alone to be able to conclude much about interactions between signals, let alone about cell type composition or mechanism etc. This section needs to be toned down and ideally additional experiments added. For instance, I don't think the authors can claim that mesoderm formation is affected purely by looking a brachyury levels (which is transient and doesn't mark mature mesoderm). I also think the correlation between two effects doesn't go far enough to allow causation to be inferred. I think this section is possibly the weakest part of the manuscript at the moment.
We thank the reviewer for this critical evaluation. We agree that our initial claims regarding the interaction between these signals required direct functional evidence and a more complete characterization of cell type composition.

To address these concerns and move beyond correlative observations, we performed a series of additional scRNA-seq experiments to pharmacologically perturb the signaling environment during the rescue window (72-120 h after aggregation). This functional approach allowed us to move beyond

transient markers such as T (Brachyury) and assess the global transcriptomic state of the resulting populations. In what follows, we briefly summarize our findings for the different perturbation experiments:

FGF Inhibition: Using the MEK inhibitor Mirdametinib (1uM) in the 300nM Retinol condition to perturb FGF signaling during the rescue window (72-120 h after aggregation), we confirmed that FGF inhibition promotes the expansion of neural progenitor fates while actively limiting mesoderm maturation (Fig. R1.6 and Fig. 6C,G, Suppl. Fig. 7A).

FGFb Supplementation: To further test this interaction, we added FGFb (20 ng/ml) for the last two days to gastruloids cultured with high retinol (600 nM). This sustained the NMP population and promoted mesoderm commitment (Fig. R1.7 and Fig. 6F,G, Suppl.Fig. 7B).

Retinol Washout: In this experiment, we grew gastruloids with 600 nM retinol and for the last 2 days (during elongation), we lowered the retinol concentration from 600 nM to 0 nM. This resulted in a shift from more ecoderm-derived towards more mesoderm-derived cells (Fig.R1.8 and Suppl. Fig. 7C, Suppl. Fig. 9D,E).

Together, these new data clarify the mechanistic link between FGF signaling and RA-mediated effects on cell fate. We have incorporated these results into the revised manuscript (Fig. 6C,F,G, Suppl. Fig. 7A,B,C and Suppl. Fig. 9D,E), toned down the language in the corresponding sections, and focused our conclusions on the definitive lineage shifts observed in our expanded scRNA-seq analysis.

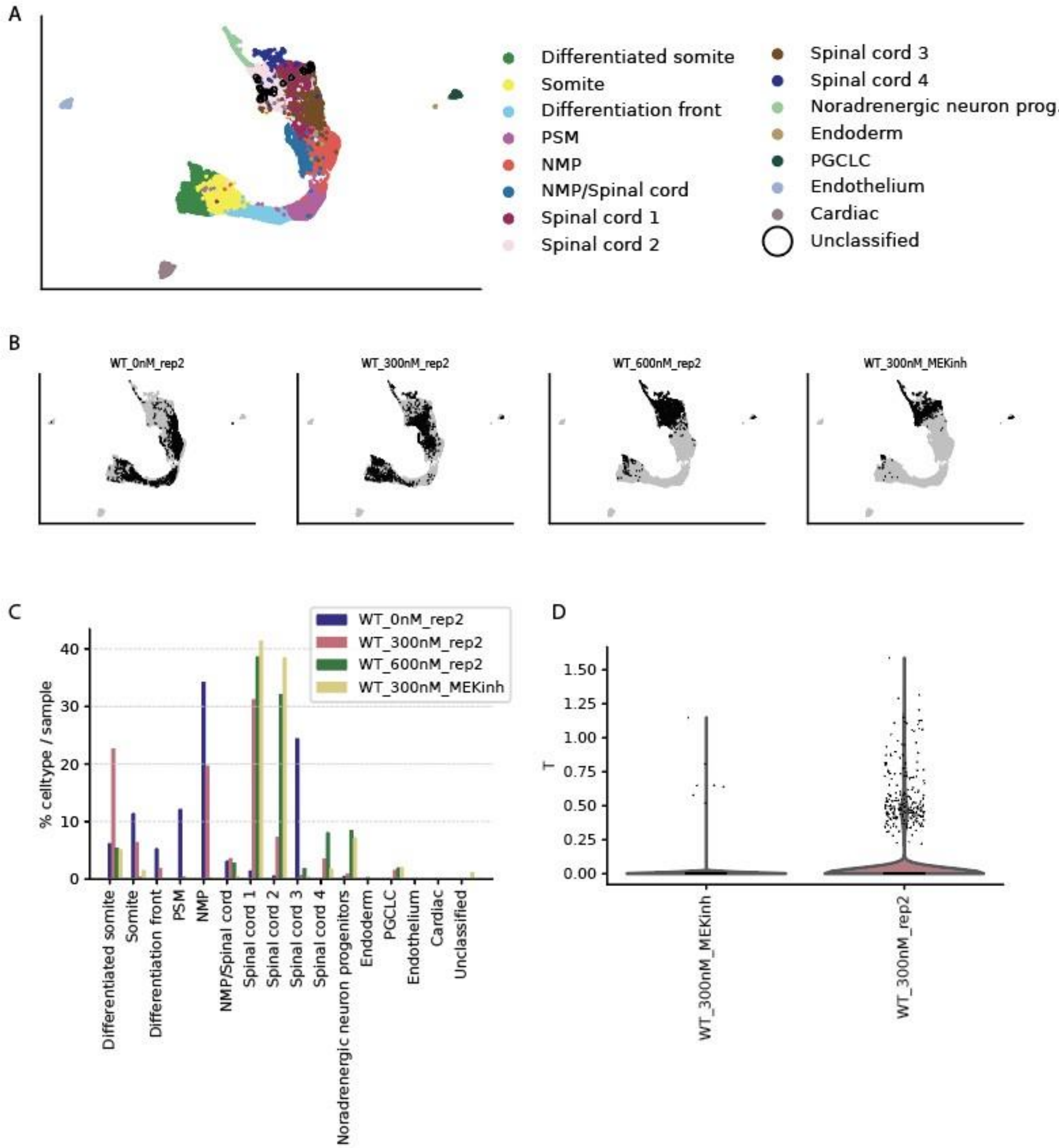

Figure R1.6. scRNA-seq of MEK inhibition experiment. A) UMAP resulting from the integration of the scRNAseq dataset generated from retinol titration experiments, MEK inhibition experiments and the dataset from Brink et al, showing cells colored by cell type identity. B) Same UMAP as in A, where cells from each indicated dataset are colored in black, while the rest of the cells are in gray. C) Barplot showing the percentage of cells in each cell type. D) Violin plot showing the distribution of T expression in all cells grouped by sample. Only the sample with MEK inhibitor and its corresponding control (300 nM retinol) are shown. Differential expression analysis between the two samples was performed using the Wilcoxon rank-sum test and subsequent Benjamini-Hochberg correction, resulting in $P<0.0001$ for T.

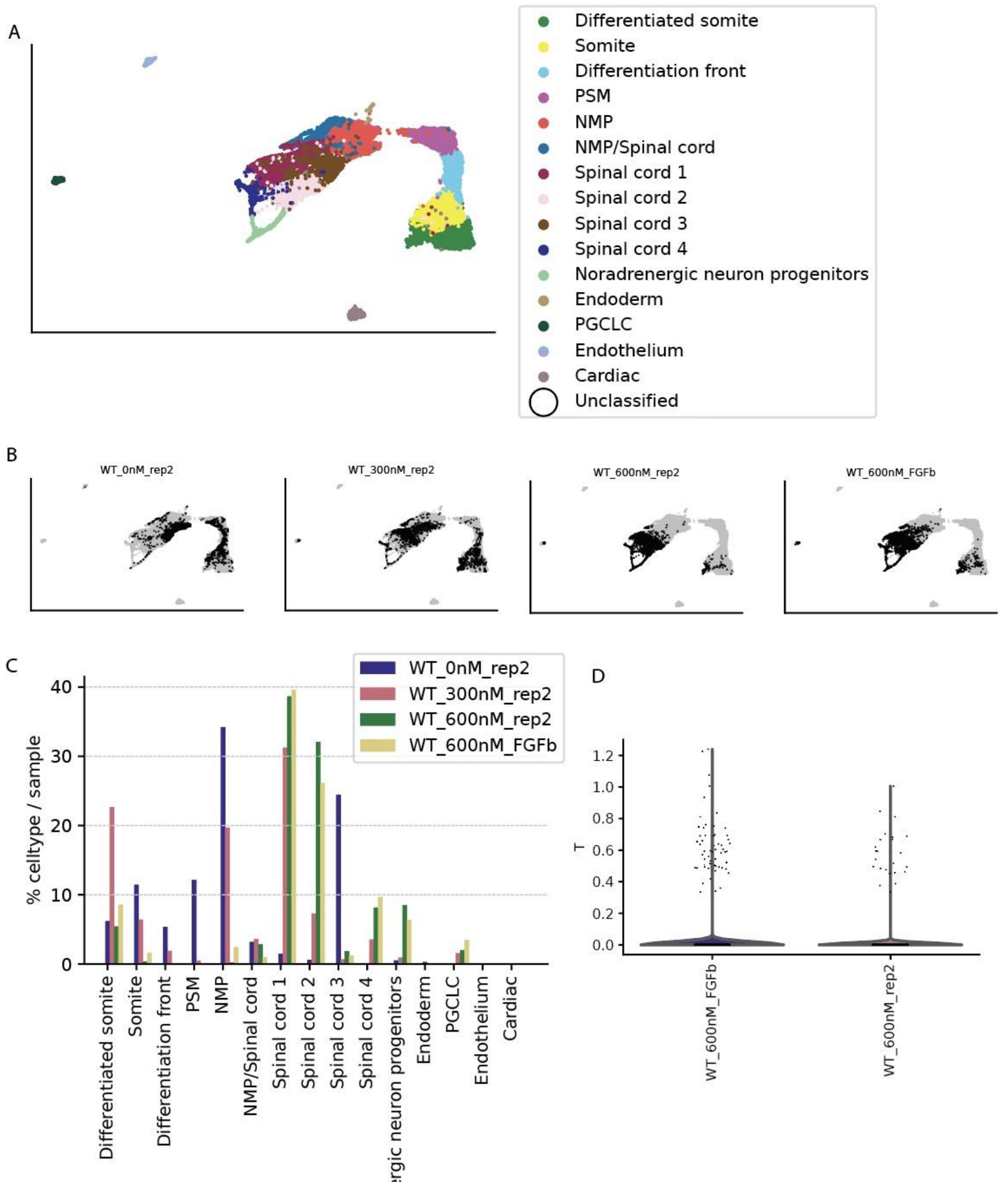

Figure R1.7. scRNA-seq of FGFb treatment experiment. A) UMAP resulting from the integration of the scRNAseq dataset generated from retinol titration experiments, FGFb treatment experiments and the dataset from Brink et al, showing cells colored by cell type identity. B) Same UMAP as in A, where cells from each indicated dataset are colored in black, while the rest are in gray. C) Barplot showing the percentage of cells in each cell type. D) Violin plot showing the distribution of T expression in all cells groupped by sample. Only the sample with FGFb treatment and its corresponding control (600

nM retinol) are shown. Differential expression analysis between the two samples was performed using the Wilcoxon rank-sum test and subsequent Benjamini- Hochberg correction, resulting in P<0.003 for T.

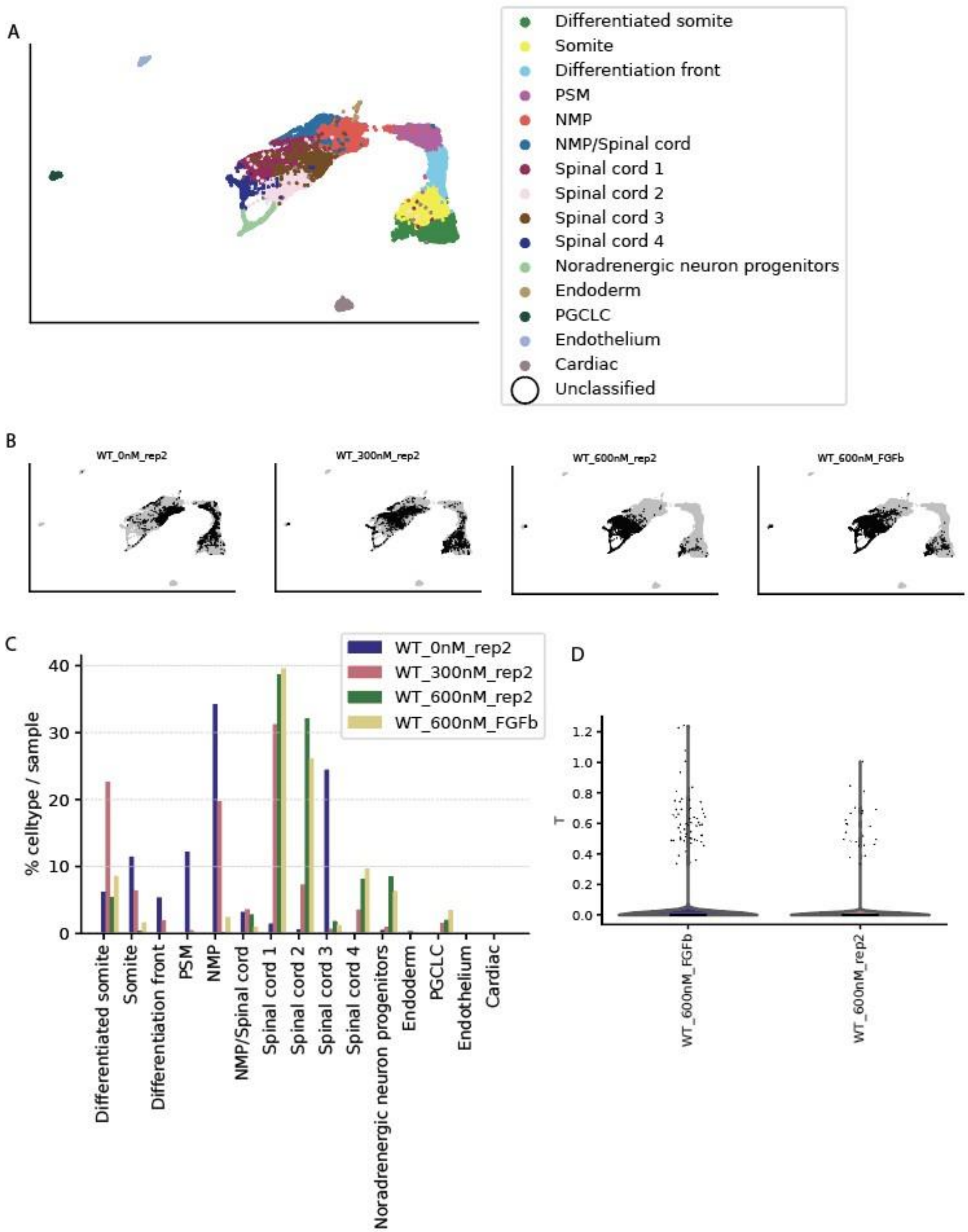

Figure R1.8. scRNA-seq of retinol washout experiment. A) UMAP resulting from the integration of the scRNAseq dataset generated from the retinol titration experiment. The experiment and the dataset from Brink et al, showing cells colored by cell type identity. B) Same UMAP as in A, where cells from each indicated dataset are colored in black, while the rest are in gray. C) Barplot showing

the percentage of the cells in each cell type for each sample. D) Violin plot showing the distribution of T expression in all cells groupped by sample. Only the sample with 0nM retinol and its corresponding control (600 nM retinol) are shown. Differential expression analysis between the two samples was performed using the Wilcoxon rank-sum test and subsequent Benjamini- Hochberg correction, resulting in P<0.0001 for T.

10) The authors don't seem to see endoderm in their gastruloids. This should be discussed in the text as the presence of 3 germ layers is thought to be the defining feature of a gastruloid.
We thank the reviewer for catching this oversight. Upon closer inspection of our scRNA-seq data and the expression of markers such as Sox17 and Foxa2, we found that endoderm cells were initially co-clustering with the PGCLC population in our UMAP projection.

In the resubmitted version of the manuscript, we integrated our dataset with previously published data (Brink et al., PMID: 32076263). This analysis allowed us to cluster the endoderm cells separately (Fig. R1.5A., cluster 14). We have updated the manuscript and figures to explicitly show the presence of all three germ layers, confirming the gastruloid identity of our aggregates. However, like in the Brink et al. dataset, our gastruloid system contains only very limited cells expressing endoderm markers.

11) I think the authors misrepresent the contextual developmental biology in their introduction. The claim that "Despite all the accumulated knowledge on FGF and RA signaling, the physiological effects of cross-talk between the two pathways in key processes such as gastrulation remain unclear". There is a well-known relationship between FGF and RA, and the authors cite many of the papers on these topics. I don't think they need to (or should) claim that little is known just to provide grounding for their research study.
We thank the reviewer for this comment. We agree that there is a well-known relationship between FGF and RA. However, many existing studies rely on Aldh1a2 (Raldh2) knockout mice, where RA concentrations cannot be fully controlled due to potential maternal contributions. While the teratogenic effects of RA in gastruloids have been described (PMID: 34425190), the physiological effects of RA signaling during mouse gastruloid development have not been systematically studied.

In our study, we used a retinoid-free medium to culture gastruloids with precise doses of retinol, allowing us to examine morphology, cellular heterogeneity, and gene expression in a controlled environment. In the resubmitted manuscript, we have modified the text to better contextualize our study and have toned down our initial claims accordingly to reflect that our work extends, rather than initiates, the study of the FGF/RA relationship. The new text reads as follows:

Revised Text:
"Many existing studies rely on knockout mice deficient in enzymes that generate RA, in which RA concentrations cannot be fully controlled due to potential maternal contributions. While the teratogenic effects of RA in gastruloids have been described (Duester, 2008; Ghyselinck and Duester, 2019; Mantziou et al., 2021), the physiological effects of RA signaling during mouse gastruloid development have not been systematically studied.

This gap highlights the need for an *in vitro* systematic study where RA or FGF signaling can be modulated at physiologically relevant concentrations. "

12) The authors should be careful to explain what is new in what they have done, and what has been shown before. For example, "Here we set up an in vitro model… using gastruloids…" makes it sound like they have devised a new protocol/system rather than made use of an existing model.
We thank the reviewer for this comment, and we agree that we need to clarify what is new in our work. The novelty of our study lies in the use of retinoid-free N2B27 as a baseline. By systematically titrating retinol levels, we provide a new understanding of its specific dose- dependent effects on gastruloid development. We have revised the manuscript text to more clearly emphasize this distinction.

Revised text:
"Here, we used an *in vitro* mouse gastruloid model to study the spatial and temporal physiological effects of RA signaling during early embryonic development (Beccari et al., 2018; van den Brink et al., 2020b; Van Den Brink et al., 2014). Gastruloids are 3D stem cell models of post- implantation stage embryos that recapitulate critical embryonic events such as symmetry breaking, axial elongation, and the emergence of several precursor cell types (Beccari et al., 2018; van den Brink et al., 2020a; Van Den Brink et al., 2014; Veenvliet et al., 2020). Since ectopic RA exposure can induce teratogenic effects (Mantziou et al., 2021), and retinaldehyde bypasses the initial regulatory steps involving retinol dehydrogenase enzymes (Duester, 2008; Sandell et al., 2007), we utilized a retinoid-free medium supplemented with specific concentrations of retinol.
This approach ensures that RA signalling is driven by the cell's own metabolic capacity: only cells expressing the required uptake and metabolic genes will convert retinol into functional RA (Duester, 2008; Kawaguchi et al., 2007; Mic et al., 2002; Mic et al., 2000; Sandell et al., 2007; Sirbu and Duester, 2006). Additionally, as commercially available media typically contain retinol, this methodology maintains comparability with standard gastruloid culture conditions while allowing us to study the role of endogenous RA signal activation *in vitro* (Beccari et al., 2018; van den Brink et al., 2020a).

13) The authors never explain why they use Retinol rather than Retinal.
We thank the reviewer for this point. We chose to use retinol as it is the primary physiological precursor in the retinoic acid (RA) signaling pathway. Unlike retinal (retinaldehyde), which bypasses the initial regulatory steps of the pathway, retinol requires active transport into the cell via STRA6 and subsequently metabolized to RA through RDH10 and RALDH2 (ALDH1A2). (Fig. 5A).

By supplementing with retinol, we ensure that RA signaling is only activated in cells that possess the necessary metabolic machinery, thereby more accurately recapitulating endogenous signaling patterns. Furthermore, retinol is the standard retinoid precursor found in commercially available media used for gastruloid protocols, allowing our results to be more directly comparable with existing studies. We have updated the manuscript to explicitly clarify this rationale:

Revised Text:
"Since ectopic RA exposure can induce teratogenic effects (Mantziou et al., 2021), and retinaldehyde bypasses the initial regulatory steps involving retinol dehydrogenase enzymes (Duester, 2008; Sandell et al., 2007), we utilized a retinoid-free medium supplemented with specific concentrations of retinol.

This approach ensures that RA signalling is driven by the cell's own metabolic capacity: only cells expressing the required uptake and metabolic genes will convert retinol into functional RA (Duester, 2008; Kawaguchi et al., 2007; Mic et al., 2002; Mic et al., 2000; Sandell et al., 2007; Sirbu and Duester, 2006). Additionally, as commercially available media typically contain retinol, this methodology maintains comparability with standard gastruloid culture conditions while allowing us to study the role of endogenous RA signal activation *in vitro* (Beccari et al., 2018; van den Brink et al., 2020a).''

14) Lines 83-86 need to refer to data in the figures to substantiate claims.
We thank the reviewer for identifying this oversight in our manuscript, we referred to the figures in the revised manuscript.

15) Line 90 should be 'time course' not 'time lapse'
We agree with the reviewer and rephrase this accordingly throughout the whole manuscript.

16) Line 138 would be good to clarify to the reader what the Rarb1 line is and what it can be used for.
The Rarb2.LacZ reporter cell line that we refer to in line 138, taken from S. Shen et al (PMID: 1338286), contains 3.4 kb of the beta-galactosidase (LacZ) coding sequence and 11 kb of pace 1Neo

gene under 1.8bp of the mRARbeta2 promoter (Fig. R1.1). In the original study, it was demonstrated that ES cells with stable integration of the mRARB2.LacZ construct express the lacZ gene in a RA-inducible manner. In our resubmission, we included the sketch of the cell line (Fig. R1.1) to Fig. 5C to explain how this cell line allows us to report retinoic acid metabolism.

17) I find the phrasing in Line 189-190 confusing. Do they mean these genes aren't expressed in the clusters that are retinol-biased?

Indeed, as the referee understands, those genes are not detected in our scRNAseq data in cells present at high doses of retinol. To clarify this, we replaced the dotplot by the one displayed in Fig. R1.9 where gene expression profile of Retinol metabolism and FGF signaling-related genes is visually inspected for the different celltypes and stratified for each retinol concentration. There, the reviewer can appreciate how Aldh1a2 is overexpressed in cell types generated at 0nM Retinol compared to those observed in the presence of Retinol. In addition, we observed that the FGF pathway–associated genes, including Fgf8, Fgf17, and Spry4, as well as retinoic acid (RA)- degrading enzyme Cyp26a1, were predominantly expressed in posterior cell populations such as neuromesodermal progenitors (NMPs), presomitic mesoderm (PSM), and NMP/spinal cord–like cells (Fig. 5B). In contrast, Fgf18 expression was detected in cell populations of the differentiation front, somites and differentiated somites, where it was co-expressed with RA signaling–associated genes, including Stra6, Rdh10, and Aldh1a2. In the resubmitted version of the manuscript we have rewritten lines 180-200 accordingly.

Revised Text:

"Single-cell RNA sequencing revealed that FGF pathway–associated genes, including Fgf8, Fgf17, and Spry4, as well as retinoic acid (RA)-degrading enzyme Cyp26a1, were predominantly expressed in posterior cell populations such as neuromesodermal progenitors (NMPs), presomitic mesoderm (PSM), and NMP/spinal cord–like cells (Fig. 5B). Notably, these populations were enriched under low retinol conditions (Fig. 5B).

In contrast, Fgf18 expression was detected in cell populations of the differentiation front, somites and differentiated somites, where it was co-expressed with RA signaling–associated genes, including Stra6, Rdh10, and Aldh1a2 (Fig. 5B).

Interestingly, in these cell clusters, the expression level of Aldh1a2 was highest at 0 nM retinol compared to higher retinol concentrations, which could be due to a negative feedback loop at higher retinol concentrations (Fig. 5B) (Dobbs-McAuliffe et al., 2004; Feng et al., 2010; Niederreither et al., 1997)."

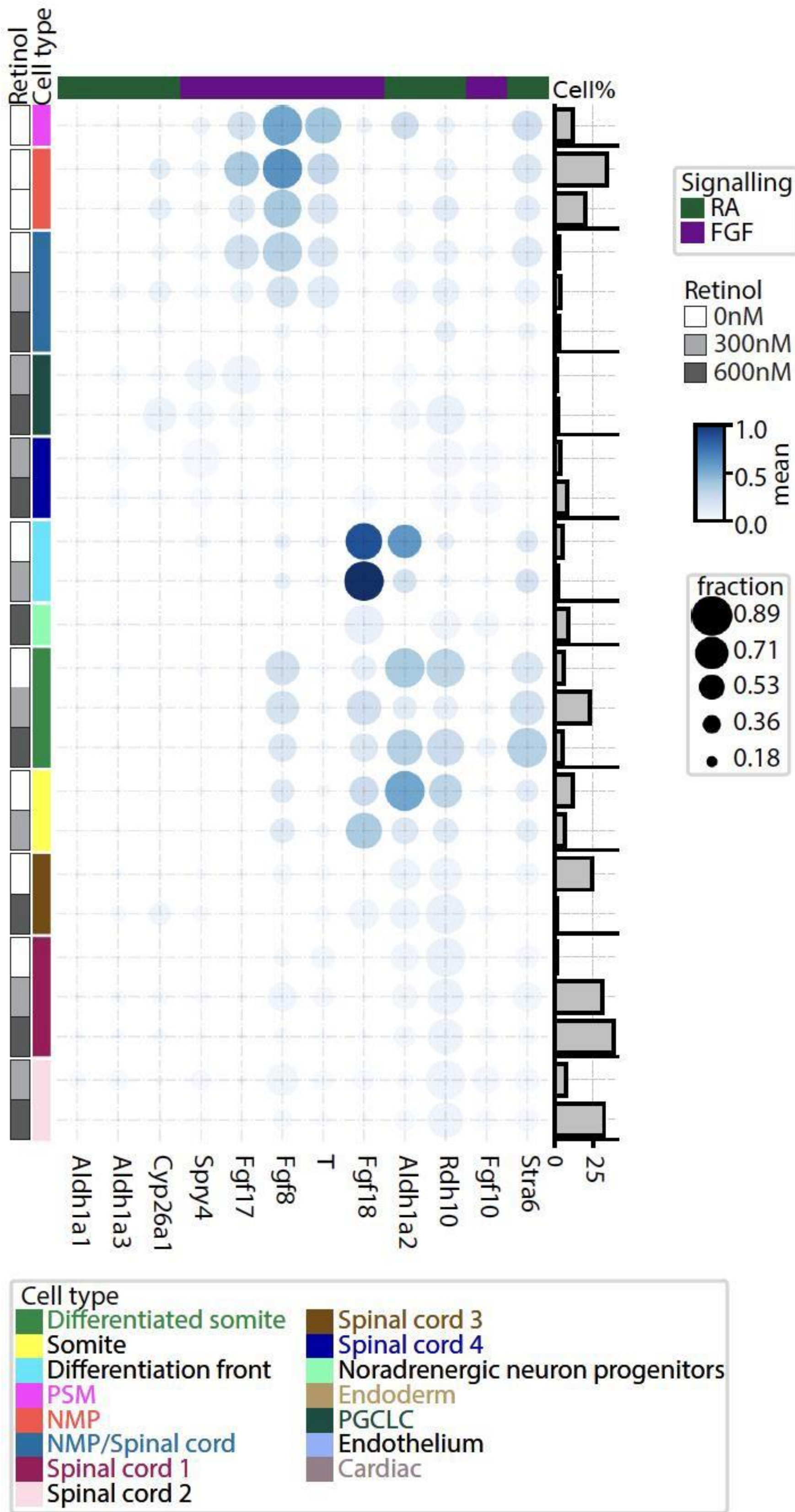
Retinol
Cell type
Cell%
Signalling
RA
FGF
Retinol
0nM
300nM
600nM
1.0
0.5
0.0
mean
fraction
0.89
0.71
0.53
0.36
0.18
Aldh1a1
Aldh1a3
Cyp26a1
Spry4
Fgf17
Fgf8
T
Fgf18
Aldh1a2
Rdh10
Fgf10
Stra6
0
25
Cell type
Differentiated somite
Somite
Differentiation front
PSM
NMP
NMP/Spinal cord
Spinal cord 1
Spinal cord 2
Spinal cord 3
Spinal cord 4
Noradrenergic neuron progenitors
Endoderm
PGCLC
Endothelium
Cardiac

Figure R1.9. Dot plot showing the expression level of retinoic acid signalling-related genes (Stra6, Rdh10, Aldh1a2, Aldh1a1, Aldh1a3, Cyp26a1),FGF signalling-related genes (Fgf17, Fgf8, Fgf10, Fgf18, Spry4),tailbud gene T in each cell type within gastruloids treated with 0 nM, 300 nM and 1200 nM retinol. Bar plot in the right side indicates the fraction of cells per condition observed in the corresponding cell type.

18) The authors claim (Line 231) that there are opposing FGF/RA gradients, but I didn't see any co-staining in the same structure, or with a universal marker like T/bra. This should be revised.
As the reviewer suggested, we have now performed co-stainings of RA-driven LacZ with either T (Brachyury) or pERK (Fig. R1.2). These data reveal a clear spatial polarization for both markers, with T/pERK signaling and RA activity occupying distinct domains along the longitudinal axis. These results provide direct evidence for the opposing signaling polarizations described in the text and have now been included in Fig. 5C of the resubmitted manuscript. In addition, we toned down our claims about gradient presence and instead discuss signalling polarization.

19) The claim of decreased FGF target gene expression (Line 233) should be toned down given they have only looked at 1 gene.
We agree with the reviewer that claims based on a single FGF target gene should be better substantiated. Because we lacked co-staining of Spry4 with a universal marker such as T (Brachyury), we have removed the Spry4 data from the manuscript.

Instead, as the reviewer suggested, we performed pERK staining in combination with the posterior marker T (Fig.R1.3 and Fig. 5E) and performed image analysis. Our data shows that retinol treatment downregulated both T and pERK expression at posterior with retinol treatment.

20) The schematic in 1A implies that the gastruloids are so big as to curl with the edge of the U-bottom plates. This is not accurate and should be revised.
We thank the reviewer for this very good catch. We replaced the schematic in Fig. 1A with the one found in Fig. R1.10.

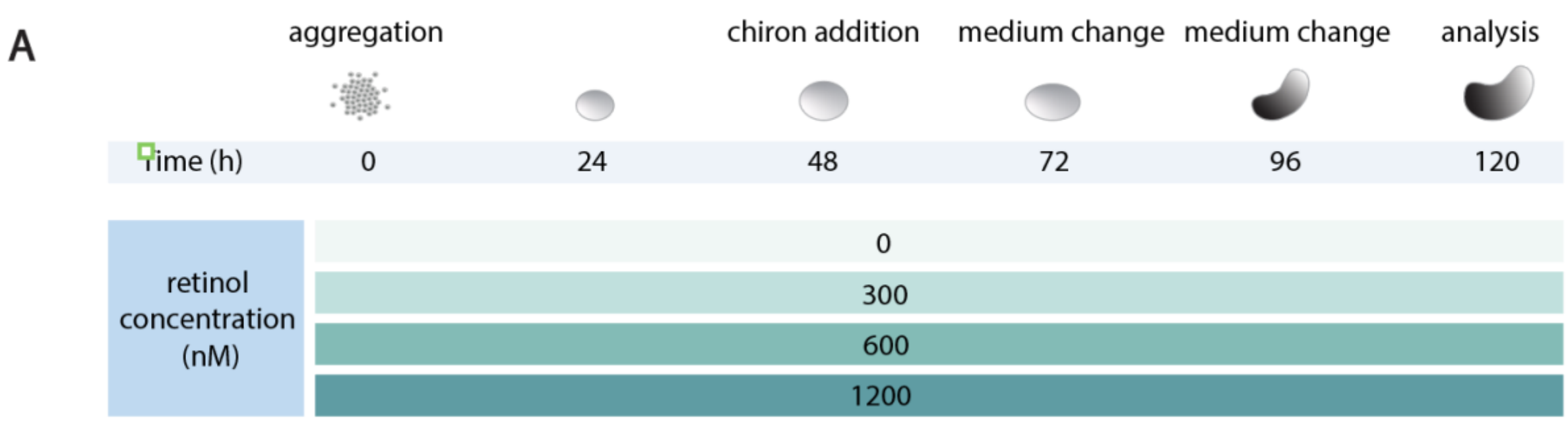

Figure R1.10. Experimental setup for gastruloid generation with different concentrations of retinol.

21) 1B it would have been good, here and throughout, to have a standard gastruloid (ie N2B27+VitA) as a positive control
This is an interesting and challenging point. We opted out from using N2B27+VitA medium to define standard gastruloids because commercially available N2B27, with proprietary composition, has batch-to-batch effects. There has been a major tour-de-force in the gastruloid community to address this point. In a recent publication (PMID: 41287934), Balayo and colleagues showed that gastruloids cultured with either home-made or commercially available N2B27 media formulations displayed different morphologies, number of cells, and even cell fate specification (with endothelial and spinal cord lineages being upregulated in home-made N2B27).

To address this point, we compared our gastruloids to the ones from Brink et al (PMID: 32076263). If we assess gastruloid similarity based on morphology, gastruloids grown at 300nM retinol have the greatest resemblance to gastruloids from Brink et al. When assessing gastruloid similarity according to the fraction of cell types detected by the scRNA-seq data, we found some interesting nuances that we would like to show the reviewer. In Brink et al., two batches of 25 pooled gastruloids were used to perform the 10X Chromium-based scRNAseq: B1 is made of old LfngT2AVenus cells, and B2 is made from freshly thawed LfngT2AVenus cells. For each batch, two replicates were processed, denoted by A or B, where PBS supplemented with different concentrations of FCS was used during cell dissociation. This information is available in the corresponding GEO repository (GSE123187). As the reviewer can see in Fig. R1.11, each of these pools generated different ratios of cell types, with the effects being larger for the somitic and the cardiac cell types. This indicates that both the passage of the cell line being used to generate gastruloids and the single cell dissociation preparation step required before library preparation can impact cell type representation in scRNAseq gastruloid datasets. Regardless of this, from Fig. R1.11 we concluded that the 300 nM retinol condition generated similar rations of somitic and NMP cell types but more spinal cord than Brink et al gastruloids.

For now, we decided not to include these results in our study. However, in the discussion, we included a few lines where we correlate gastruloid morphology with gene expression profiles and cell type configuration based on the observations we gather from our experiments.

Revised text:

"Retinol concentration thus regulates both cell type composition and morphology in gastruloids, highlighting possible crosstalk between differentiation and growth. At low levels (0 nM), gastruloids are small and predominantly mesodermal, indicating limited cellular diversity and expansion (Fig. 3,4). Intermediate levels (300 nM) generate the largest gastruloids with a diverse mix of mesodermal and ectodermal cell types (Fig. 3,4), supporting coordinated growth. In contrast, high retinol levels (600–1200 nM) drive neural dominance, resulting in smaller and simpler structures (Fig. 3,4), likely due to reduced cellular diversity. "

NOTE: We have removed unpublished data that had been provided for the referees in confidence using data from van den Brink SC, Alemany A, van Batenburg V, Moris N, Blotenburg M, Vivié J, Baillie-Johnson P, Nichols J, Sonnen KF, Martinez Arias A, van Oudenaarden A. Single-cell and spatial transcriptomics reveal somitogenesis in gastruloids. Nature. 2020 Jun;582(7812):405-409. doi: 10.1038/s41586-020-2024-3. Epub 2020 Feb 19. Erratum in: Nature. 2020 Mar;579(7799):E11. doi: 10.1038/s41586-020-2113-3. PMID: 32076263.)

Figure R1.11. Ratio of cell types detected for different 10X Chromium experiments. Datasets indicated with a cross come originally from PMID: 41287934, while circles refer to samples produced in this study.

22) Typo in 1C à 'length'

We thank the reviewer for catching the typo, which has now been corrected.

23) 3B show individual data points, including differences between experimental runs

We thank the reviewer for this suggestion. We have modified all of our FACS plots accordingly, which is shown in Fig. R1.12. In the revised graphs, individual data points are now plotted, and each independent experimental run is represented by a different color to clearly illustrate the consistency and differences between replicates. They can be seen in Fig. 4A,B,Suppl. Fig. 3A,B and Suppl. Fig 5B)

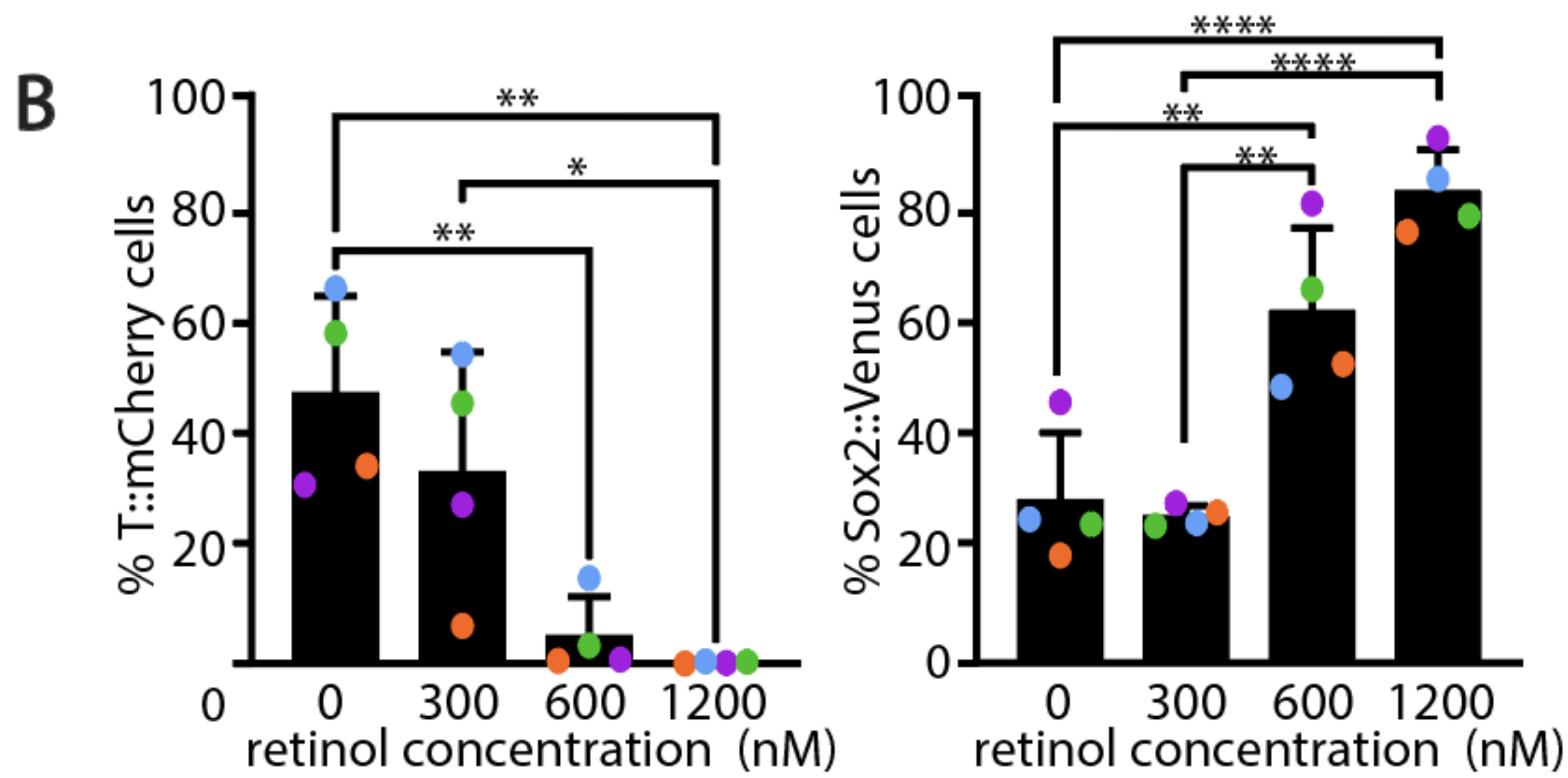

Figure R1.12. Example of a revised FACS plot. Percentages of T::mCherry- and Sox2::Venus- positive cells in 120 h gastruloids cultured at different concentrations of retinol, as determined by FACS. A total of 40–45 gastruloids were analyzed per experimental group (N = 4). Each color represents a biological replicate. Graphs show mean ± SD. Statistical comparisons between groups were performed using one-way ANOVA followed by Tukey's post hoc test. * p< 0,05; ** p< 0,005; **** p< 0,0001for both graphs.

24) It wasn't clear to me at first glance that Clusters 0/1 on Fig 2B were related to PC1 and the other to PC2. Could be clarified on the figure itself.
All genes shown in the heatmap in Fig. 2B have significant loadings to PC2, and consequently all clusters are related to PC2. To avoid further confusions here and in the accompanying Sup. Fig. 1A, we relabeled clusters as PC1-1, PC1-2, etc. In addition, we revised our text to clarify this.

25) 4D would be helpful to show ISH on a white background, given that it is a dark stain We thank the reviewer for this suggestion. We have carefully considered using a white background; however, we found that it reduced the contrast between the dark ISH signal and the dense gastruloid tissue at bigger magnifications. We chose the black background because it provides a clearer definition of both the staining patterns and the overall morphology of the structures.

To demonstrate this, we have provided a comparison of the two background options for the reviewer's reference below (Fig. R1.13). We believe the black background remains the most effective way to represent these data in the manuscript.

However, for the new stainings we performed microsopy at lower magnification with a white background (Fig. R1.2; Fig. 5C).

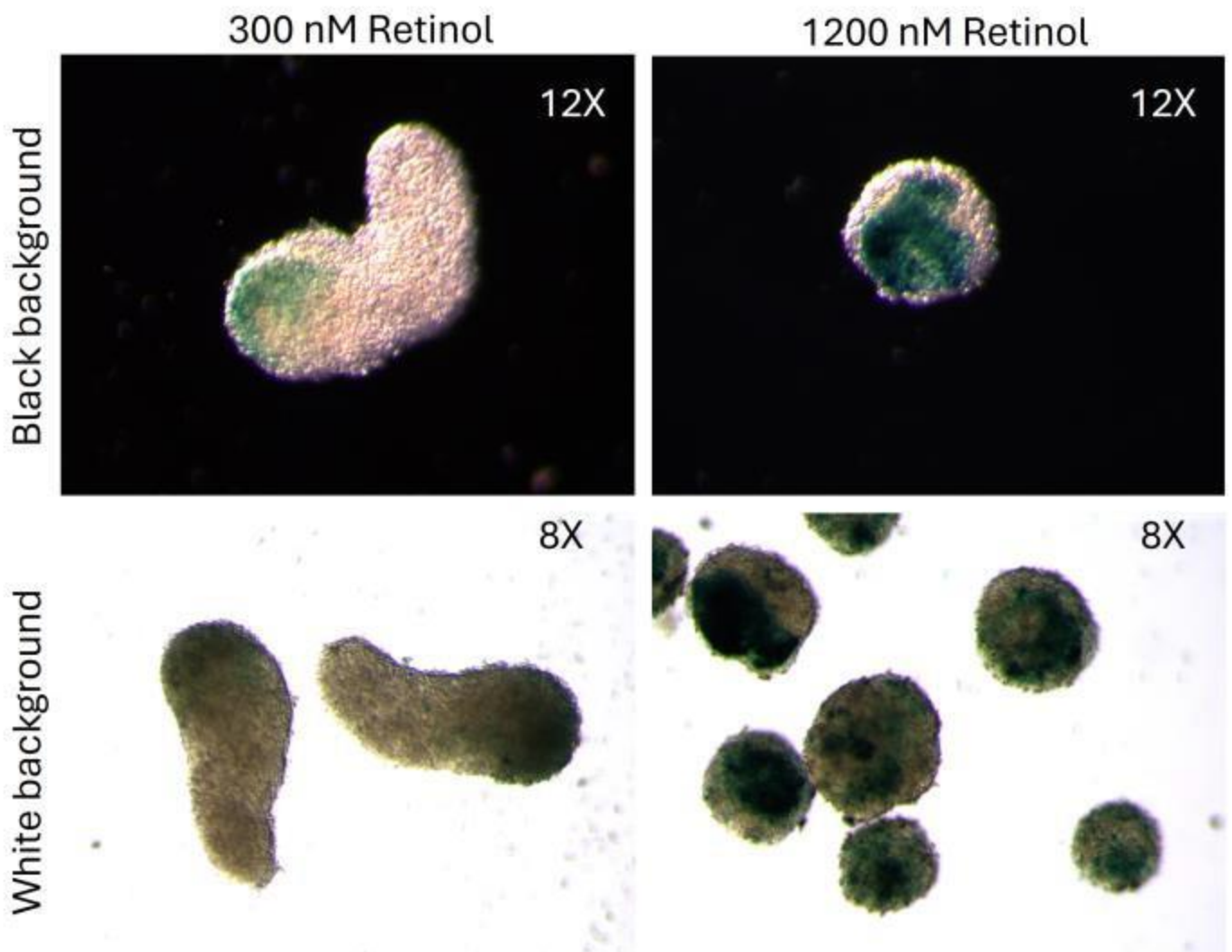

Figure R1.13. Gastruloids grown with 300 nM and 1200 nM retinol observed with black and white background under a steriomicroscope.

26) 5A/5D it isn't clear if retinol is maintained while the FGF treatments are added. Clarify in text and figures as this will affect which control is appropriate.
We apologize that this was not clear in the manuscript. Retinol presence is maintained throughout the gastruloid culture. In the resubmitted manuscript, we replaced the panels in Fig. 6A,D and Suppl. Fig. 8A by Fig. R1.14 and improved the text accordingly.

A

Time (h)	0–72	72–120
retinol concentration (nM)	300	300
	300	300 +Mirdamet. 0.5 µM
	300	300 +Mirdamet. 1 µM

D

Time (h)	0–72	72–120
retinol concentration (nM)	600	600
	600	600 + FGFb 10ng/ml
	600	600 + FGFb 20ng/ml

Figure R1.14. Experimental design to examine the effect of MEK inhibitor (A) and FgFb (B) on gastruloid elongation and cell fate.

27) The 'dips' in morphometric measurements, eg 5C or 5F, seem to correlate with media changes. Does this mean it is measuring cell debris more than the edges of the gastruloids? If so, the method should be revised as it is not accurately conveying gastruloid morphology. We thank the reviewer for this observation. The "dips" in Figures 5C and 5F are transient technical artifacts of the Incucyte S3 system. These occur when plates are temporarily removed for media changes, causing minor shifts in positioning or autofocus.
To maintain data integrity and full transparency, we have chosen to display the continuous raw data rather than smoothing the curves. Importantly, they do not alter the overall growth trends or our interpretations. We have added a description of this effect to the Methods section, which can be seen below.

Revised method:
"At time points involving medium changes or treatment with small molecule inhibitors or proteins, gastruloids were displaced from the center of the wells, impairing image focus. This resulted in transient drops in signal that represent technical artifacts rather than biological effects. "

28) 6C I see no evidence of a gradient from the data shown.
We decided to tone down our claims about gradients. Accordingly, in the revised text, we refer to

“localisation” and “signaling polarization” instead of “gradients”.

Reviewer 2

1) Line 80-86: This paragraph seems to suggest that excessive retinol gives rise to suboptimal gastruloid morphology, using elongation as the primary criterion. The authors should define the morphological features of 'optimal gastruloids' to begin with. This would especially help the readers unfamiliar with the model system. Including characterization of the major cell types in gastruloids (e.g. immunofluorescence images) and clarification of which are lacking at the extreme ends of the retinol concentration range would furthermore better link morphology to cell type development.

The definition of the “optimal gastruloid” is still an ongoing debate in the field. In a recent publication (PMID: 41287934), Balayo and colleagues showed that gastruloids cultured with either home-made or commercially available N2B27 media formulations displayed different morphologies, number of cells, and even cell fate specification (with endothelial and spinal cord lineages being upregulated in home-made N2B27). Altogether, this makes it very challenging (and even intimidating) to define what the morphological features of an optimal gastruloid are, which we believe can also be confounded by the cell types present in the system. To address this point, we included a few lines in the discussion of the manuscript in which we correlate gastruloid morphology with gene expression profiles and cell type configuration based on the observations we gather from our experiments.

Additionally, as the reviewer suggests, we performed new co-stainings at different retinol concentrations that allow to simultaneously validate the results from scRNA-seq analysis and to locate the different cell types along the gastruloid anterior-posterior axis (Fig. R2.1). These results have been included as a new figure in the resubmitted manuscript (Fig. 4A,B; Fig. 5E).

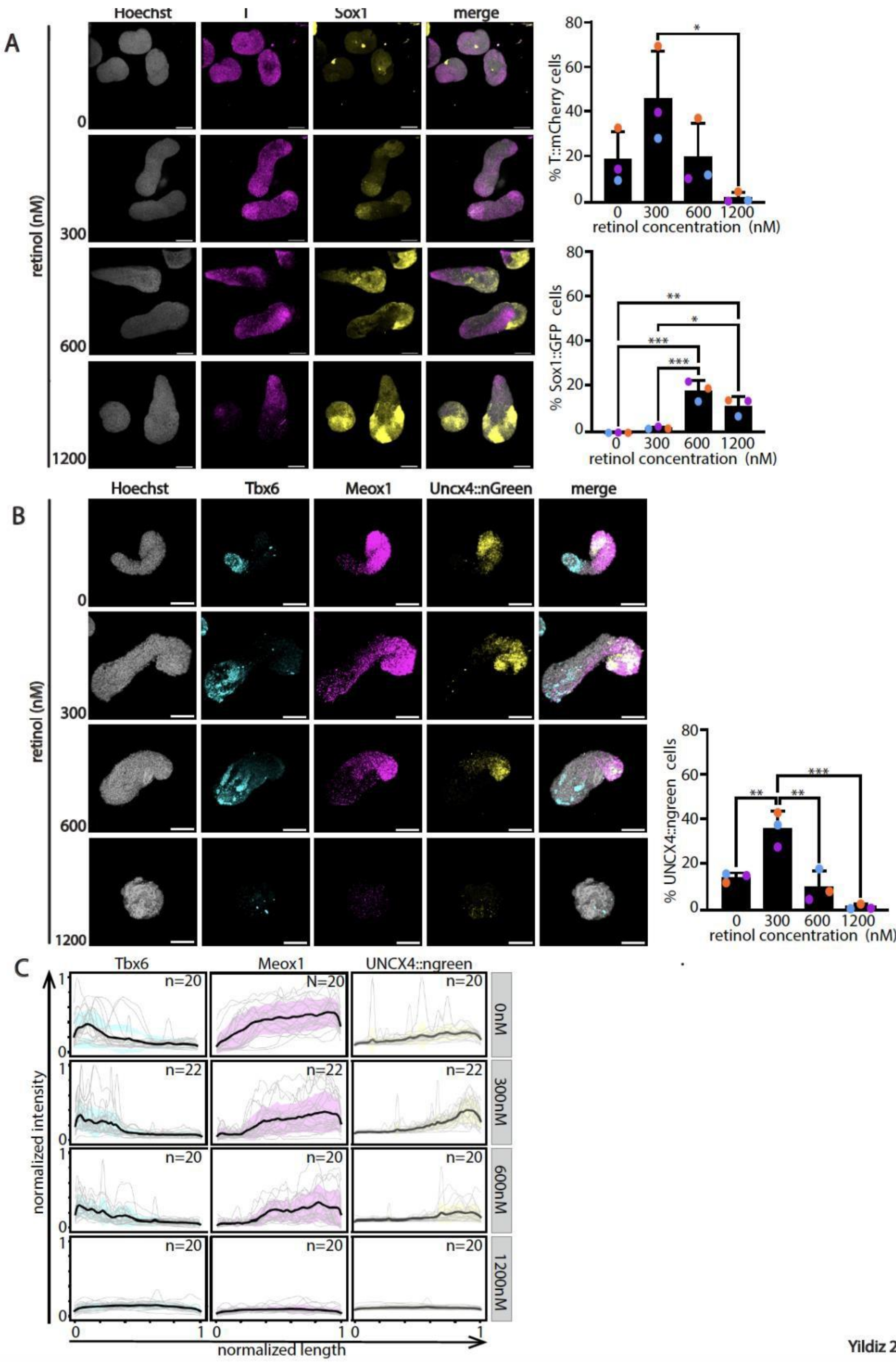

Yildiz 2026_Figure 4

Figure R2.1 (A) (left) Representative images of 120 h gastruloids expressing T::mCherry (magenta) and Sox1::GFP (yellow), treated with different concentrations of retinol. Grey: Hoechst. Scale bars: 200 µm. (right) Percentages of T::mCherry- and Sox1::GFP-positive cells in 120 h gastruloids, as measured by FACS. Between 40–45 gastruloids were used per experimental group (N = 3). Each colored dot represents a biological replicate. Graphs show mean ± SD. Experimental groups were compared using one-way ANOVA, with P-values adjusted by Tukey's test (P *< 0.05; **P < 0.01; ***P < 0.001). (B) (left) Representative images of Tbx6 (cyan), Meox1 (magenta), and Uncx4.1::NeonGreen (yellow) in gastruloids treated as in (A). Grey: Hoechst. Scale bars: 200 µm. (right) Percentage of Uncx4.1::NeonGreen-positive cells in 120 h gastruloids measured by FACS. Between 40–45 gastruloids were used per experimental group (N = 3). Each colored dot represents a biological replicate. Graphs show mean ± SD. Experimental groups were compared using one-way ANOVA, with P-values adjusted by Tukey's test (**P < 0.01; ***P < 0.001). (C) Relative intensity profiles of Tbx6 (cyan), Meox1 (magenta), and Uncx4.1::NeonGreen (yellow) from posterior to anterior in gastruloids shown in (C). Each gray line represents one gastruloid; shaded areas indicate standard deviation, and black lines indicate the mean. (n = 20 (0 nM retinol), n = 22 (300 nM retinol), n = 20 (600 nM retinol), n = 20 (1200 nM retinol); N = 2 independent biological experiments)

2) Fig 1C: Typo: Length

We thank the reviewer for catching this typo, which we corrected in the resubmitted manuscript.

3) Line 87-114: The principal component analysis is a bit obtuse and its value unclear. Please improve the overall clarity of this section.

We thank the reviewer for pointing out that the original phrasing was unclear. We have revised the corresponding sentences to improve clarity, replacing the previous phrasing with more standard PCA terminology (e.g., "loadings"), which we believe makes the description more transparent. This PCA was used as an unsupervised approach to identify the main sources of variation in the dataset and enabled us to distinguish temporal effects from retinol-dependent effects and to subsequently identify genes associated with each. The value of the PCA has also been explained in the resubmitted manuscript.

4) Line 102-114: Listing possible associated cell types for each cluster does not seem to be the most efficient way to interpret the variance along PC2, particularly since co-expression information for these genes is not available. In figure 2A, samples from different retinol concentrations are already separated along PC2 at 0 hours and the difference increases with time. This pattern and the associated genes are not well described in this paragraph. Are the genes driving this variance at 0 hours similar to those at later time points? Could the authors provide example gene names?

To explain the different profiles of dynamic gene expression seen in Fig. 2B (referred to as clusters), we used Metascape analysis (shown in Supplementary Figure 1C). There, our top hits related to neuron projection, germ cell proliferation, heart development, etc. For these reasons, we listed possible associated cell types. However, we agree with the reviewer that we should not stress cell types so much and rather talk about biological processes. In the revised manuscript, we contextualize the power of Metascape analysis in the text.

We thank the reviewer for pointing out at the fact that distinct retinol samples already segregate in the PCA space at the first time point (we think the reviewer means 24h, since at 0 h we have only stem cells in stem cell media, which it is the common origin for all the different retinol conditions). To address their question, we performed differential gene expression analysis using DESeq2 for all samples against stem cells. As the reviewer can appreciate in Fig. R2.2, 65% of the genes that are driving differential expression at 24h are still differentially expressed at 120h. However, these only represent about 15% of the genes that are differentially expressed at at the later timepoint (Fig. R2.2D). When interrogating in detail which genes are differentially expressed in all conditions at 24h and all conditions in 120h, we get a set of 72 genes which include Cdx2, Nkx1- 2 and Neurod1 among others. We included this figure in the Suppl. Fig 2 and Suppl. Table 2.

© 2026. Published by The Company of Biologists under the terms of the Creative Commons Attribution License (https://creativecommons.org/licenses/by/4.0/).

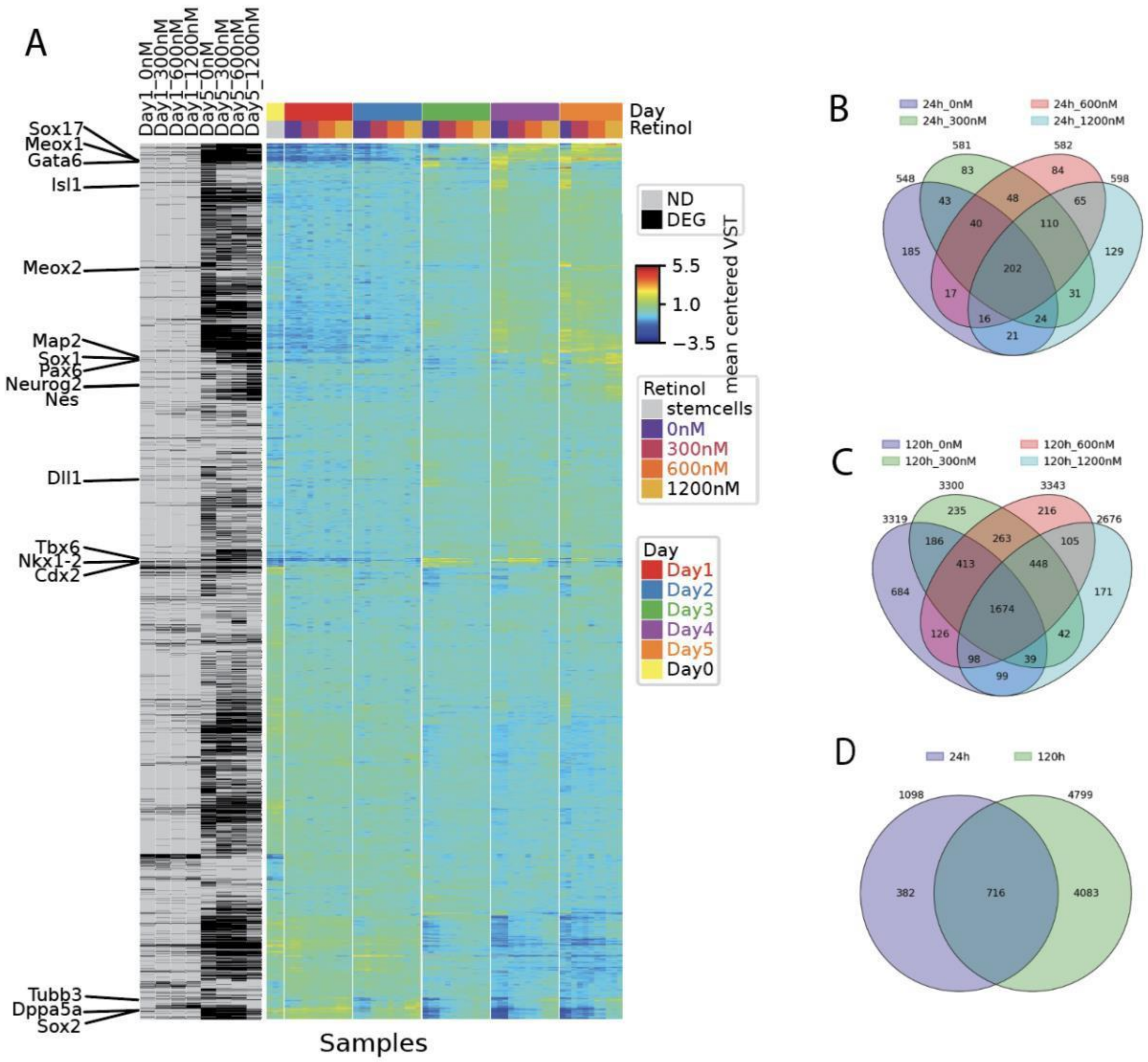

Figure R2.2. Differential gene expression dynamics across retinol conditions and time points.
(A) Heatmap showing the mean-centered VST across different time points and retinol concentrations for genes differentially expressed between one or more samples or compared to stem cells. Genes differentially expressed in one sample are indicated in black in the left annotation bars; others are indicated in grey. Differential expression annotation is shown for samples obtained at 24h and at day 120h. (ND: non-differentially expressed; DEG: differentially expressed genes)
(B) Venn diagram comparing the numbers of differentially expressed genes with different retinol concentrations at 24h after aggregation. (C) Venn diagram comparing the numbers of differentially expressed genes with different retinol concentrations at 120h after aggregation. (D) Venn diagram showing the intersection between pooled differentially expressed genes identified at 24 h and 120 h after aggregation across all retinol conditions.

5) Line 120-121: Why wasn't the dual reporter mentioned in Line 131 chosen? One gastruloid - same information, less biological noise between varying morphologies and an almost direct comparison within the same sample. Could the authors kindly explain and elaborate?
We agree with the reviewer that, preferably, the same cell line should be used for both experiments. However, the Sox2::Venus, T::mCherry double reporter cell line that we used for FACS analysis was too sensitive to the conditions (e.g. time-lapse imaging) in the Incucyte S3, and consequently gastruloid formation was disturbed. For this reason, we could not use this line for any experiment carried out in the Incucyte S3 system.

To address this comment, we performed FACS analysis with gastruloids generated from

© 2026. Published by The Company of Biologists under the terms of the Creative Commons Attribution License (https://creativecommons.org/licenses/by/4.0/).

Sox2::mScarlet3 and T::mCherry reporter cell lines to support time-lapse imaging data (Fig. R2.3, now included in Suppl. Fig. 3 in the manuscript). We explained in the Methods section.

Revised method
"The Sox2:Venus T::mCherry (Veenvliet et al., 2020) double reporter cell line was highly sensitive to Incucyte S3 conditions and failed to form gastruloids. Therefore, two alternative cell lines, T:mCherry (Deluz et al., 2016) and Sox2:mScarlet3 reporter mESCs (from this study), were used for time-lapse imaging. "

6) Line 120-121: Can the same line be used for both the time-lapse and the FACS experiment? It would make the comparison between the results easier, especially since it was mentioned that each cell line behaves slightly different.
As explained in point 5, the Sox2::Venus, T::mCherry double reporter cell line does not survive culturing in Incucyte S3 imaging system. To support our time-lapse imaging data, we performed FACS analysis with the same 2 reporter lines (Sox2::mScarlet3 and T::mCherry), which were used for time-lapse imaging. In this analysis, we measured the average median intensity of T:mCherry and Sox2::mScarlet3 positive cells. We included these graphs in Fig.R2.3 and in Suppl. Fig.3.A,B.

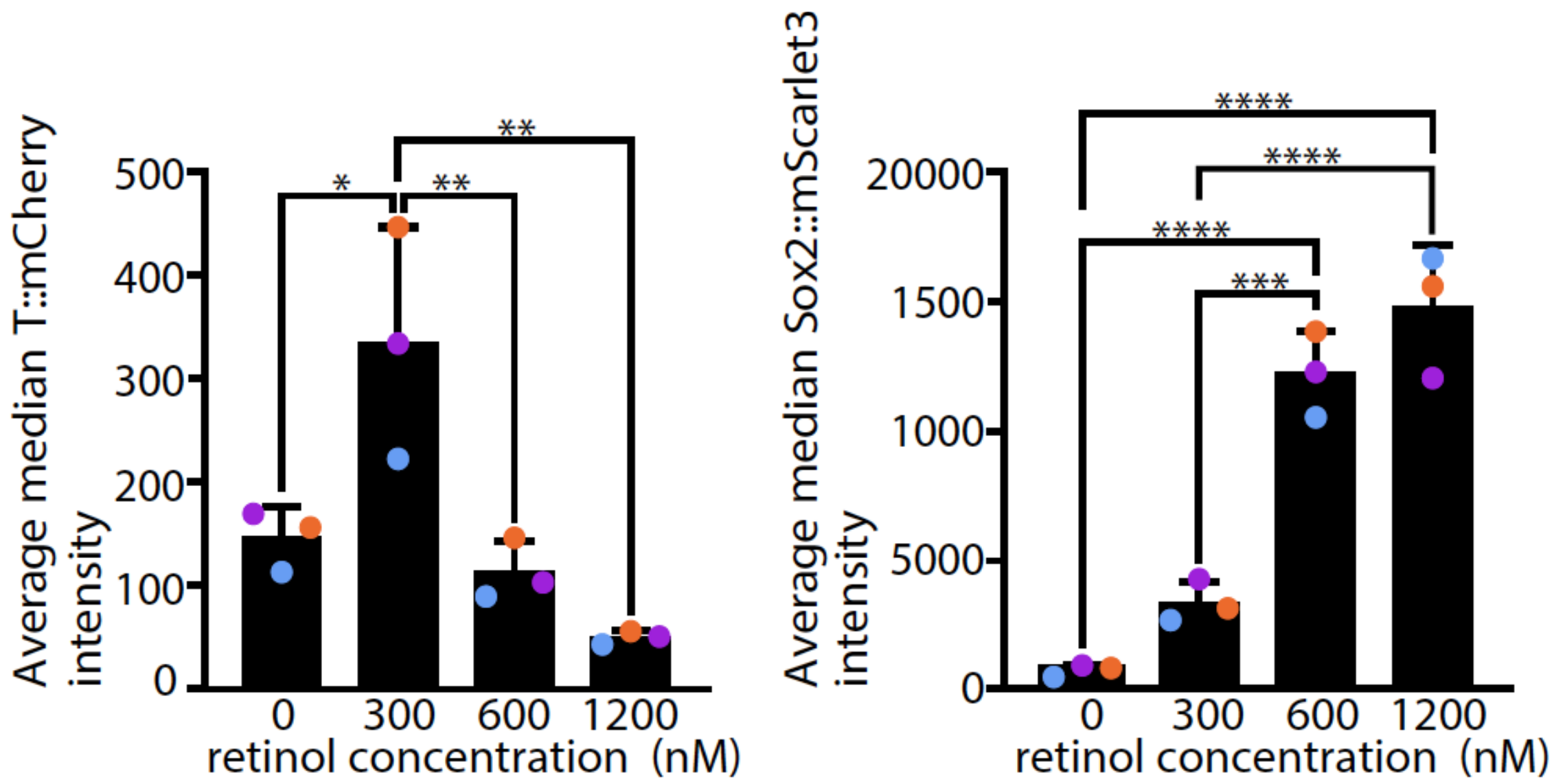

Figure R2.3. The average median intensity of T::mCherry (from T::mCherry Sox1::GFP cell line) (at left) and Sox2::mScarlet3 positive cells (at right) in gastruloids treated with different doses of retinol, 120h after aggregation, as revealed by FACS. 40-45 gastruloids were used for each experimental group (N=3). Each color shows a biological replicate. Graphs show mean and SD. Experimental groups were compared with one-way ANOVA and P-values were corrected with the Tukey test, *p<0.05, **p<0.01, ***p<0.001, ****p<0.0001 for both graphs.

7) Line 122, Figure 2D and 2E: In the graphs there are noticeable troughs at certain time points (for T::mCherry, at 48h, 72h, 96h). Are these reflecting technical artifacts, or represent a biological effect?
We thank the reviewer for this observation. These dips are a consequence of the Incucyte S3 system: whenever there is a culture system, gastruloids need to be taken out of the microscope, resulting in dips in graphs. We prefer to display the data with these dips, since in our opinion, it does not go against the interpretation of our data.
We have added a description of this effect to the Methods section, which can be seen below.

Revised method:
"At time points involving medium changes or treatment with small molecule inhibitors or proteins, gastruloids were displaced from the center of the wells, impairing image focus. This resulted in transient drops in signal that represent technical artifacts rather than biological effects. "

© 2026. Published by The Company of Biologists under the terms of the Creative Commons Attribution License (https://creativecommons.org/licenses/by/4.0/).

8) According to Figure 2C, T expression seems to reach a peak at 72 hours and decreases at the transcriptional level. In Figure 2D, reporter activity, which should correlate with the transcriptional activity of T, overall increases over time. The authors should address and explain this discrepancy. Furthermore, the T::mCherry signal seems to have high variability (localization of replicate values). Adding confidence intervals may be useful for visualizing the difference in signal intensity relative to the variability.
We thank the reviewer for this observation. The discrepancy between Fig. 2C and Fig. 2D (which is stronger for the 600nM Retinol condition) might also be a consequence of how different data modalities are normalized: in Fig. 2C, we show the variance stability transformed data per each time point in the bulk RNA sequencing data. In contrast, in Fig. 2D, we analyzed the total fluorescence intensity (i.e, protein levels) of the T::mCherry-positive area and normalized the data to the minimum intensity as 0 and the maximum intensity as 1. Another explanation can be given by the fact that two different cell lines were used in each experiment: in bulk RNAseq data, the cell line used originates from an IB10 background, while for the imaging data it originates from a CGR8. Nevertheless, the overall trend in both experiments is that more retinol negatively correlates with T expression at 120h. To support further the findings described in this figure, we now also provided FACS analysis (Fig. R2.3 and Suppl. Fig. 3A,B).

9) Figure 2D: There seems to be massive cell death even at 96h and 120h? Could the authors please explain why that may be and what alternatively the 'halo' around each gastruloid is? The Sox2 line doesn't look as bad btw. Interestingly, the 300nmol retinol condition has increased T activity compared to the 0nmol. No statement on whether the intensity difference between 300nmol and 0nmol at 120h is significant on the graph or not but it looks visibly significant (maybe they just swapped a column by accident; it can also be a matter of normalizing signal by gastruloid area as it seems visibly correct at 120 on the representative images just not in the graph).

We agree with the reviewer and think that the “halo” around gastruloids observed at 96h and 120h in Fig. 2D consists indeed of dead cells. This is because of the suboptimal conditions with the time-lapse imaging in the S3 IncuCyte system. The T::mCherry reporter cell line we used in Fig. 2D is more sensitive to these conditions than the Sox2::mScarlet3 reporter line we used.

We thank the reviewer for noting the differences in signal intensity between the 0 nM and 300 nM retinol conditions in T:mCherry reporter cell line. In response to this suggestion, we performed additional FACS analysis using the same cell line (T::mCherry;Sox1::GFP mESCs) treated with different doses retinol (Fig. R2.3, Suppl. Fig. 2). Consistent with the reviewer’s observation, the average median intensity of T:mCherry was highest at 300 nM retinol in this specific cell line and decreased with retinol treatment. In the revised manuscript, we have included this graph as a Suppl. Fig 3 alongside the time-lapse imaging data and incorporated this valuable observation into the main text.

10) Line 132, Figure 3B: Figure 2D, which measured the T::mCherry signal shows that the reporter activity is present at 120hrs even at the highest concentration, although at a lower level. The percentage of mCherry-positive cells, measured by FACS, appears equal to or very close to 0. The authors should explain the discrepancy or provide additional data.
We thank the reviewer for this accurate observation. We believe that the observed differences are attributable to the use of different cell lines: in Fig. 2D, we used a T:mCherry Sox1::GFP reporter cell line, whereas the data in Fig. 3B were obtained using the double reporter Sox2::Venus, T::mCherry cell line. Unfortunately, the double reporter line does not survive under the culturing conditions required for the Incucyte S3 system. Nevertheless, both cell lines recapitulate the same phenotype, namely that higher retinol concentrations inhibit T expression at later time points.

In response to the reviewer’s suggestion, we performed additional FACS analysis using the same cell lines employed for time-lapse imaging, treated with different concentrations of retinol (Fig. R2.3 and Suppl. Fig. 3). Consistent with the reviewer’s observation, the average median intensity of T::mCherry at 1200 nM retinol was detectable but remained the lowest.

11) Line 133-134: The authors claim that the FACS results are in line with their time-lapse data but Figure 3B for T count is contradicting with what can be seen in the Graph for Figure 2D. The jump of higher T intensity from 0 to 300 is opposite of the count seen in FACS sorting. If

© 2026. Published by The Company of Biologists under the terms of the Creative Commons Attribution License (https://creativecommons.org/licenses/by/4.0/).

they were to repeat the time lapse experiment with the double reporter would they get the same result? They could also do FACS again with one set of SOX2 only reporter gastruloids and a set of T only reporter.

We thank the reviewer for noting the differences in signal intensity between the 0 nM and 300 nM retinol conditions in the T::mCherry reporter cell line. As explained in the previous point, different reporter lines were used for time-lapse imaging and FACS analysis.

In response to this suggestion, we performed additional FACS analysis using the same cell line treated with a range of retinol concentrations (Suppl. Fig. 3A). Consistent with the reviewer's observation, the average median intensity of T::mCherry was highest at 300 nM retinol in this specific cell line, in agreement with the time-lapse imaging data. In the revised manuscript, we have included this graph as Supplementary Fig. 3A alongside the time-lapse imaging results and incorporated this observation into the main text.

Additionally, we performed FACS analysis using the Sox2::mScarlet3 reporter cell line (Suppl.Fig.3B), measuring the average median intensity of Sox2:mScarlet3. In line with the time-lapse imaging results, we observed a dose-dependent increase in Sox2:mScarlet3 reporter activity by FACS.

12) Line 138: Why did the authors not use a wild-type line but another reporter?
By using the Rarb2-LacZ cell line, we expected to be able to annotate directly cells responding to retinoic acid by quantifying transcription levels of the LacZ reporter gene, or alternatively, the Neomycin (Fig. R2.1). Unfortunately, neither of these two transcripts were detected in our dataset. We included this observation in the resubmitted manuscript.

NOTE: Figure provided for reviewer has been removed. It showed Figure 1 from Shen S, van den Brink CE, Kruijer W, van der Saag PT. Embryonic stem cells stably transfected with mRAR beta 2-lacZ exhibit specific expression in chimeric embryos. 1992.Int J Dev Biol. Dec;36(4):465-76. PMID: 1338286. We have removed unpublished data that had been provided for the referees in confidence.

Figure R2.4. Schematic representation of the construct containing 1.8 kb of mRARb2 promoter (open box). 3.4 kb of lacZ coding sequence (filled box) and 1.1 kb of pace 1Neo (hatched box). (Figure extracted from PMID: 1338286)

Revised text:
"To investigate how retinol affects cellular identity, we performed single-cell RNA sequencing (scRNA-seq) on gastruloids 120 h after aggregation. Gastruloids were generated from the Rarb2.LacZ reporter line (Shen et al., 1992) and treated with different retinol concentrations; however, LacZ reporter expression was not detected in the sequencing data."

13) Figure 3E: What are the trunk progenitors that seem to only be present at 300nM? In supplemental figure 3C they have a similar expression level as 600nM, but the cells are not present in the 600nM cell population in Figure 3E.
We realized that in our submitted manuscript the single cell RNA dataset, which consisted of independent 10X runs for each retinol condition, could suffer from batch effects. We therefore decided to produce a new scRNA-seq dataset using the 10X on-chip-multiplexing approach. This strategy allowed us to process all samples simultaneously under the same conditions, thereby reducing the risk of batch effects. We replaced the data presented in manuscript by this batch effect-free dataset which has also been analyzed together with previously published scRNAseq data (Fig. 3A). Cell type calling was performed by transferring the labels from the published dataset to ours, when possible, or by exploring gene ontologies and intersection analysis of differentially expressed genes detected in the mouse embryo atlas (Pijuan-Sala et al. PMID: 30787436) (Fig.R2.5, Suppl. Fig4) With the new analysis, trunk progenitors are now labeled as pre-somitic mesoderm (PSM) and are not exclusive from a single retinol concentration (Fig. R2.5). We are convinced that the effects observed in the resubmitted version of the manuscript are biological and not a result of data processing.

© 2026. Published by The Company of Biologists under the terms of the Creative Commons Attribution License (https://creativecommons.org/licenses/by/4.0/).

NOTE: We have removed unpublished data that had been provided for the referees in confidence using data from van den Brink SC, Alemany A, van Batenburg V, Moris N, Blotenburg M, Vivié J, Baillie-Johnson P, Nichols J, Sonnen KF, Martinez Arias A, van Oudenaarden A. Single-cell and spatial transcriptomics reveal somitogenesis in gastruloids. Nature. 2020 Jun;582(7812):405-409. doi: 10.1038/s41586-020-2024-3. Epub 2020 Feb 19. Erratum in: Nature. 2020 Mar;579(7799):E11. doi: 10.1038/s41586-020-2113-3. PMID: 32076263.) and Pijuan-Sala, B., Griffiths, J.A., Guibentif, C. et al. A single-cell molecular map of mouse gastrulation and early organogenesis. Nature 566, 490–495 (2019). https://doi.org/10.1038/s41586-019-0933-9

Fig. R2.5. Label transfer for cell type identification. A) (left) UMAP obtained from integrating the scRNAseq dataset generated in our study together with the 10X Chromium dataset from Brink et al, showing cells colored by Leiden cluster identity. (right) Heatmap showing, for each annotated cell type, the fraction of cells in each Leiden cluster. This is restricted to cells from the Brink et al dataset. B) Same UMAP where cells coming from Brink et al or from the different Retinol culture conditions obtained in this study are colored in black, and the rest in gray. C) Dotplot showing common number of markers between the Leiden clusters shown in panel A and cell types annotated in the scRNAseq mouse embryo atlas from Pijuan Sala et al. Red indicates significant, while gray indicates not significance (P<0.001). The size of the dot is proportional to the number of common markers.

14) Figure 3G: In addition to Hes7 which is an oscillating gene, the authors should consider using e.g. TBX6 or MSGN1 as Paraxial Mesoderm Markers.
We thank the reviewer for catching this oversight.
In the resubmitted version of the manuscript, we replaced the line plots with the dot plot shown in Fig. R2.6. Therefore, Hes7 is not shown anymore as a marker. Dot plot can be found in Fig. 3B.

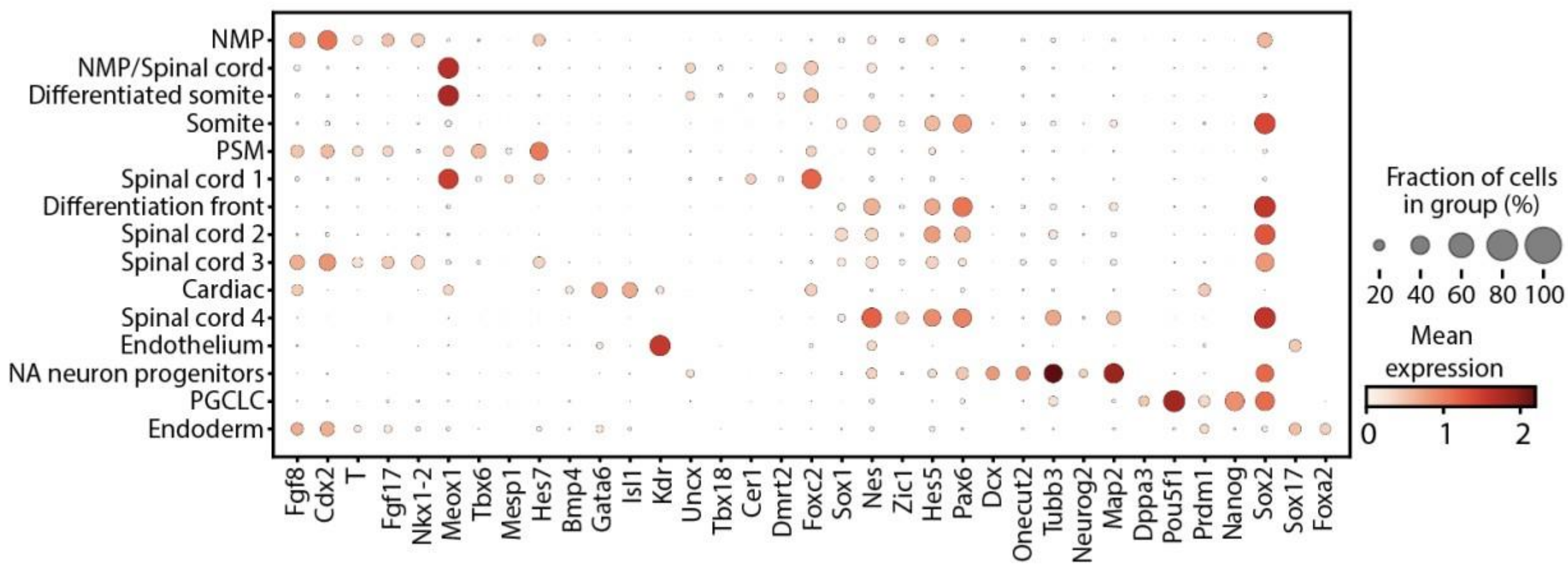

Figure R2.6. Dot plot representing the percentages of different cell types within 120h gastruloids for different concentrations of retinol.

15) Line 151-153, Figure 3F-I: Retinol 600nM and 1200nM seem to have some cell types in common. Have the authors examined within the same cell type cluster gene expression differences across retinol concentrations? It may also be informative to visualize the time-course expression trends of genes involved in RA synthesis across different retinol concentrations and see in parallel with the ones for FGF8.
This is an excellent idea. Following the reviewer suggestion, we focused on cell types that are present in two or more retinol conditions and performed differential gene expression analysis across retinol concentrations.

We first focused on NMP, Somites and Differeniation front, which are observed at 0 and 300 nM retinol. Subsequent gene ontology analysis revealed that Wnt Signaling (through the presence of Wnt6, Wnt10a and Wnt3a) and Neural crest differentiation (Wnt3a, Tbx6, hes5 and Dll3) were upregulated at 0nM in the NMP. No other significant terms were identified using the MSigDB Hallmark database from 2020.

© 2026. Published by The Company of Biologists under the terms of the Creative Commons Attribution License (https://creativecommons.org/licenses/by/4.0/).

Next, we focused on Spinal cord 1-4 populations, which we combined as a single cell type for this analysis. Results showed that genes belonging to the Gene Ontology Nervous Systems Development (GO:0007399) (such as Nlgn1, Adgrv1, Cntn6, St8sia2, Ncan, Pax3, Nav2, Pou3f2, Pou3f3, Dclk1, Dnm3, Nav3, Erbb4, Pax7, Fabp7, Spock2, Dner, Spock1, Fat4, Nes, Neurog1, Mdga1, Gpm6b) were upregulated at 600nM, suggesting that high retinol promotes maturation of neural progenitors. Relevant examples of these genes are given in Fig. R2.7. These results are also discussed in the resubmitted version of the manuscript, and shown in Suppl. Fig. 4D.

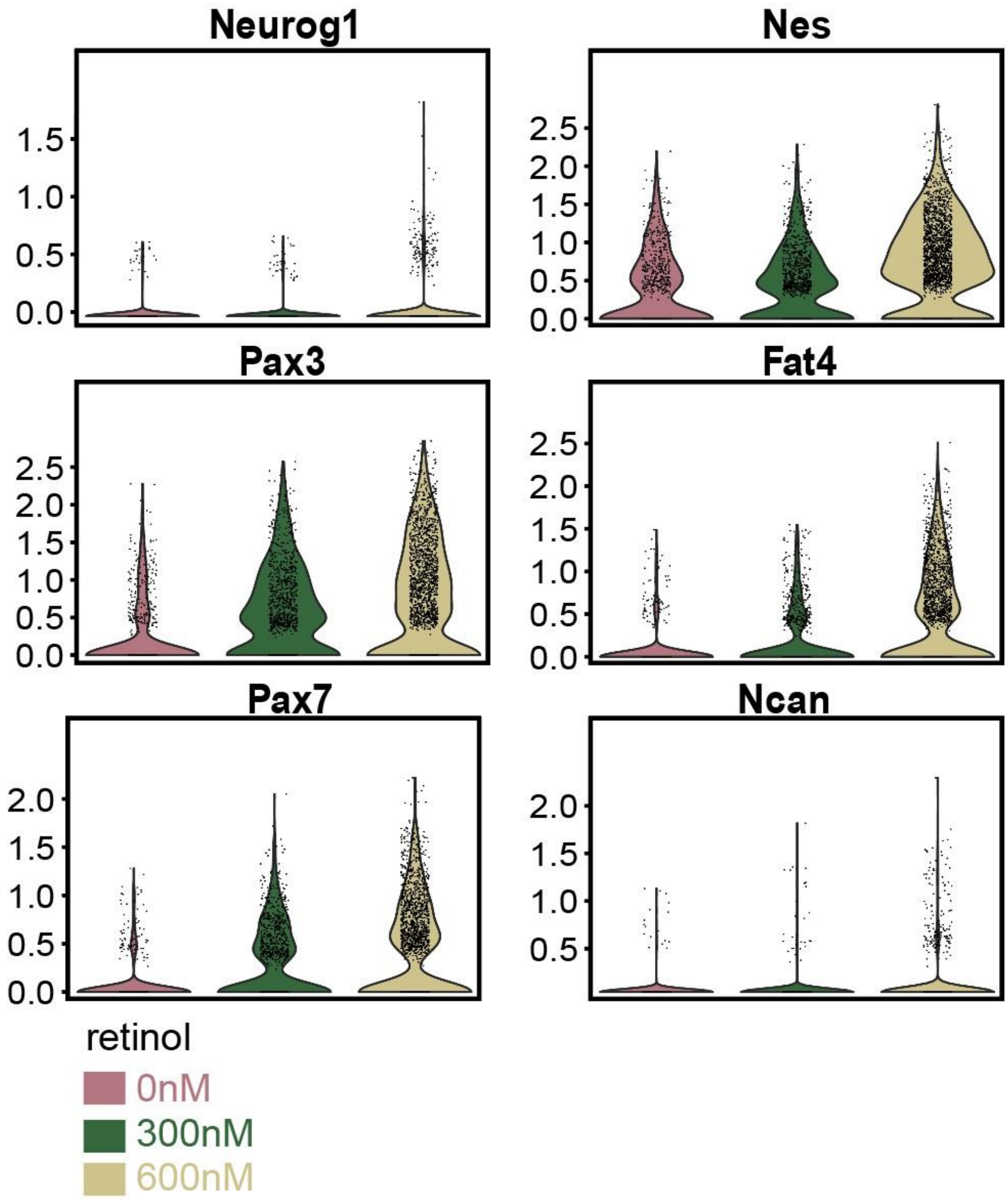

Figure R2.7. Violin plots showing expression of markers from nervous system development detected in the combined spinal cord population, grouped by retinol concentration.

16) Figure 4B & C: Which gastruloids were used to produce this data? All the different conditions at 120h?
We apologize to the reviewer that this was not clear in our manuscript. To generate panels B and C in Fig. 4, we included all retinol conditions, as the observed cell types are highly condition-specific. However, in the revised manuscript, we explored expression patterns of the same genes in stratified cells not only by cell type but also by retinol condition, as shown in Fig. R2.8 and Fig. 5B in the revised version.

© 2026. Published by The Company of Biologists under the terms of the Creative Commons Attribution License (https://creativecommons.org/licenses/by/4.0/).

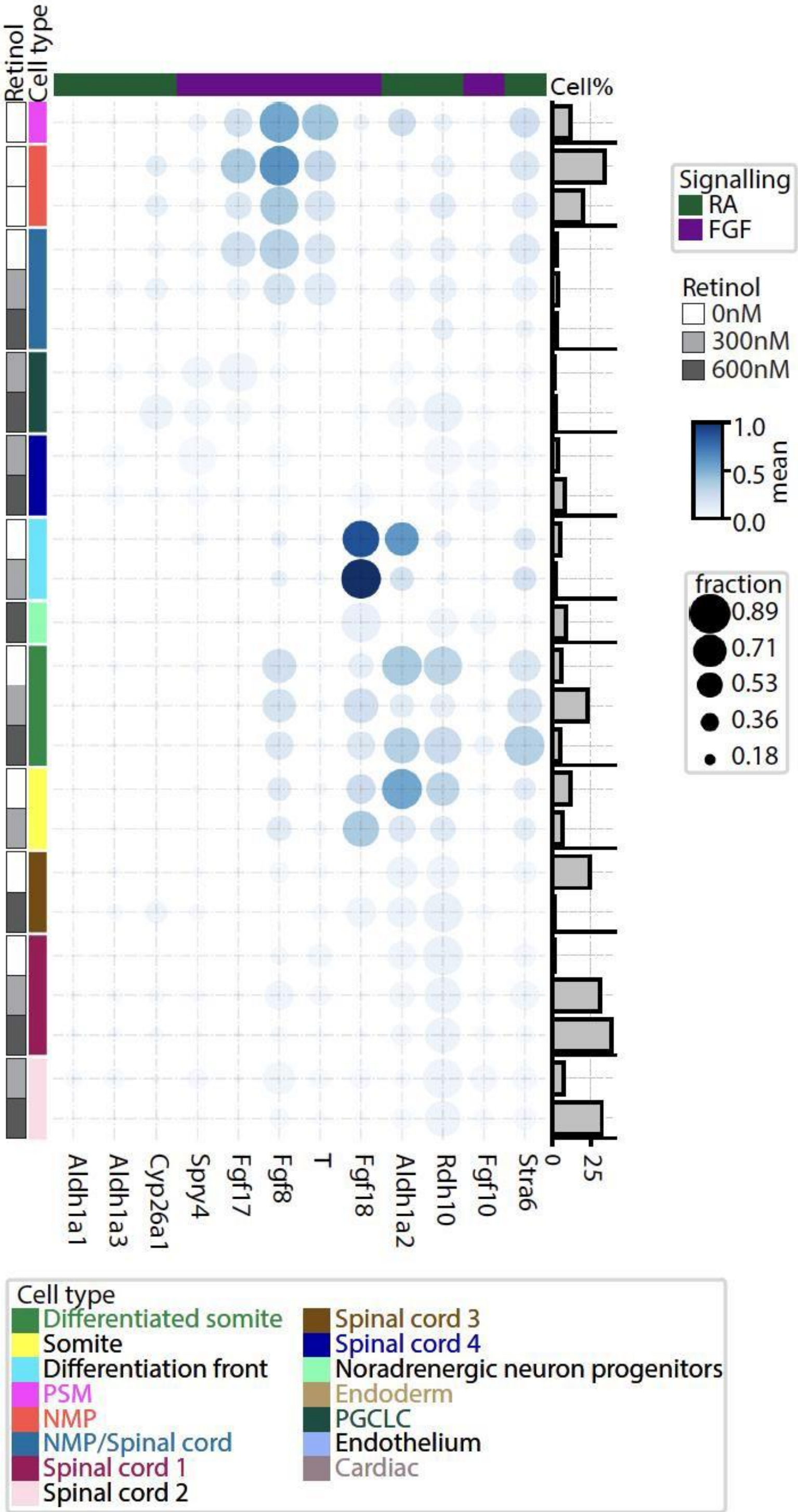

Figure R2.8. Dot plot showing the expression level of retinoic acid signalling-related genes (Stra6, Rdh10, Aldh1a2, Aldh1a1, Aldh1a3, Cyp26a1) and FGF signalling-related genes (Fgf17, Fgf8, Fgf10, Fgf18, Spry4) and tailbud gene T in each cell type within gastruloids treated with 0 nM, 300 nM and 1200 nM retinol. Bar plot in the right side indicates the fraction of cells per condition observed in the corresponding cell type

17) Figure 5: Gastruloid Surface, Elongation Index is never defined. Colors bleed into each other on the graphs making it hard to read.

We thank the reviewer for their comment on the time-lapse imaging graphs. In the revised

© 2026. Published by The Company of Biologists under the terms of the Creative Commons Attribution License (https://creativecommons.org/licenses/by/4.0/).

manuscript, we have adjusted the visualization by dimming the colors of the data points, which we believe improves the clarity and readability of the graphs. New graphs can be seen in Fig. 2D,E; Fig. 6C,F; Suppl. Fig. 8 and Suppl. Fig. 9.
In our new manuscript, we explained how gastruloid surface and elongation index are quantified in the method section, as seen below.

Revised method
"Image analysis was performed using the commercially available Incucyte S3 Spheroid Image Analysis Module (Sartorius). Three parameters were assessed: gastruloid surface area was measured as the “largest brightfield object area (μm²)”, elongation index was quantified using the “brightfield object average eccentricity”, and reporter activity was determined as the “largest brightfield object red/green integrated intensity (RCU × μm²)”.

18) Figure 5B & E: Scale bar info is missing.
Scale bar information and corresponding dimensions have been added to panels 5B and 5E in the figure legends.

19) Figure 6B, D: I don't see how they can make these claims from the data they've shown and discussed. The authors should provide more references and other data sets e.g. from mouse embryo data to back up their proposed model. How good does the single cell data of their in vitro model cluster with the in vivo data?
We thank the reviewer for the critical eye. When performing cell type calling in our scRNAseq data, we performed intersection analysis of differentially expressed genes detected in a well-established scRNAseq-based mouse embryo atlas (PMID: 30787436). This information has now been included in the Suppl. Fig. 4C.

Since the stainings and FACS experiments for cell-specific markers were carried out only in gastruloids (Fig.R2.9, Suppl. Fig.4), the revised manuscript no longer includes a direct comparison with mouse embryos. Accordingly, Fig. 7 (Fig. R.2.10 in this rebuttal) has been adapted to present and summarize the results obtained solely from gastruloid analyses.

20) Considering how in Figure 6, the authors are making comparisons with mouse embryos, it would have been nice to see some staining of representative genes of members of the different cell populations described in Figure 3 and 6D, either by antibody staining or in-situ hybridization.

We agree with the reviewer that, to support the schematic presented in the previous Figure 6, stainings for representative marker genes are necessary. In the revised version of the manuscript, we therefore performed the following stainings and corresponding FACS analyses (Fig. R2.9), which are presented throughout the manuscript and summarized in Fig. R.2.10 and Fig. 7.

Tail bud: T::mCherry (imaging and FACS) (Fig. 4A; Fig. 4E and Supplementary Fig. 5A,B)
Mesoderm: Tbx6 (imaging) (Fig. 4B,C)
Presomitic mesoderm: Meox1 (imaging) (Fig. 4B,C)
Somites: Uncx4 (UNCX4::nGreen) (imaging and FACS) (Fig. 4B,C)
Neural cells: Sox2::Venus and Sox::GFP (imaging and FACS) (Fig. 4A and Supplementary Fig. 5A,B)

As these stainings and FACS analyses were performed only in gastruloids, we did not include a direct comparison with mouse embryos in the revised manuscript. Instead, Figure 7 (Fig. R2.10 in the rebuttal) now summarizes and depicts our observations exclusively in gastruloids.

© 2026. Published by The Company of Biologists under the terms of the Creative Commons Attribution License (https://creativecommons.org/licenses/by/4.0/).

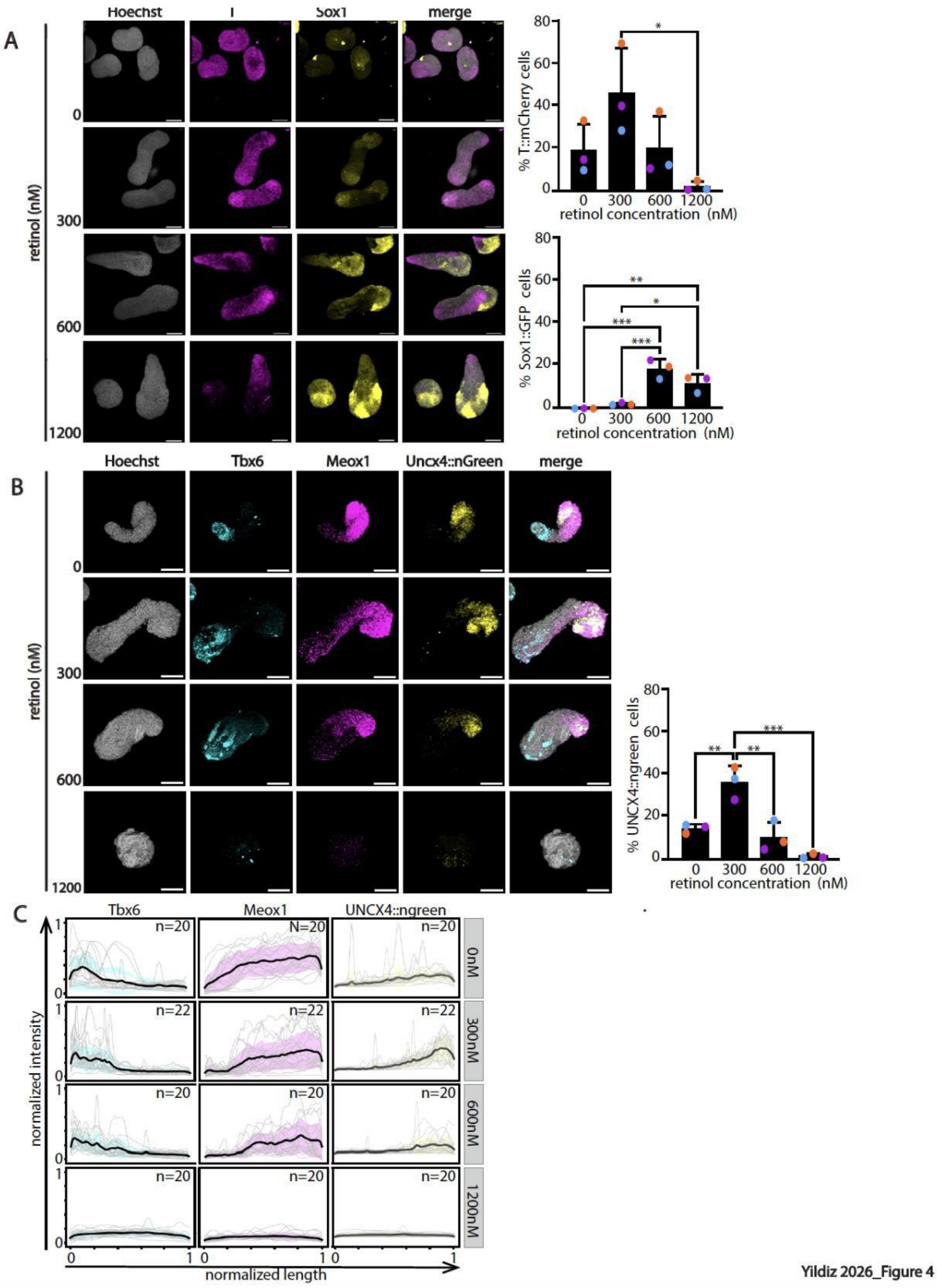

Figure R.2.9 (A) (left) Representative images of 120 h gastruloids expressing T::mCherry (magenta) and Sox1::GFP (yellow), treated with different concentrations of retinol. Grey: Hoechst. Scale bars: 200 µm. (right) Percentages of T::mCherry- and Sox1::GFP-positive cells in 120 h gastruloids, as measured by FACS. Between 40–45 gastruloids were used per experimental group (N = 3). Each colored dot represents a biological replicate. Graphs show mean ± SD. Experimental groups were compared using one-way ANOVA, with P-values adjusted by Tukey's test (P *< 0.05; **P < 0.01; ***P < 0.001). (B) (left) Representative images of Tbx6 (cyan), Meox1 (magenta), and Uncx4.1::NeonGreen (yellow) in gastruloids treated as in (A). Grey: Hoechst. Scale bars: 200 µm. (right) Percentage of Uncx4.1::NeonGreen-positive cells in 120 h gastruloids measured by FACS.

© 2026. Published by The Company of Biologists under the terms of the Creative Commons Attribution License (https://creativecommons.org/licenses/by/4.0/).

Between 40–45 gastruloids were used per experimental group (N = 3). Each colored dot represents a biological replicate. Graphs show mean ± SD. Experimental groups were compared using one-way ANOVA, with P-values adjusted by Tukey's test (**P < 0.01; ***P < 0.001). (C) Relative intensity profiles of Tbx6 (cyan), Meox1 (magenta), and Uncx4.1::NeonGreen (yellow) from posterior to anterior in gastruloids shown in (C). Each gray line represents one gastruloid; shaded areas indicate standard deviation, and black lines indicate the mean. (n = 20 (0 nM retinol), n = 22 (300 nM retinol), n = 20 (600 nM retinol), n = 20 (1200 nM retinol); N = 2 independent biological experiments)

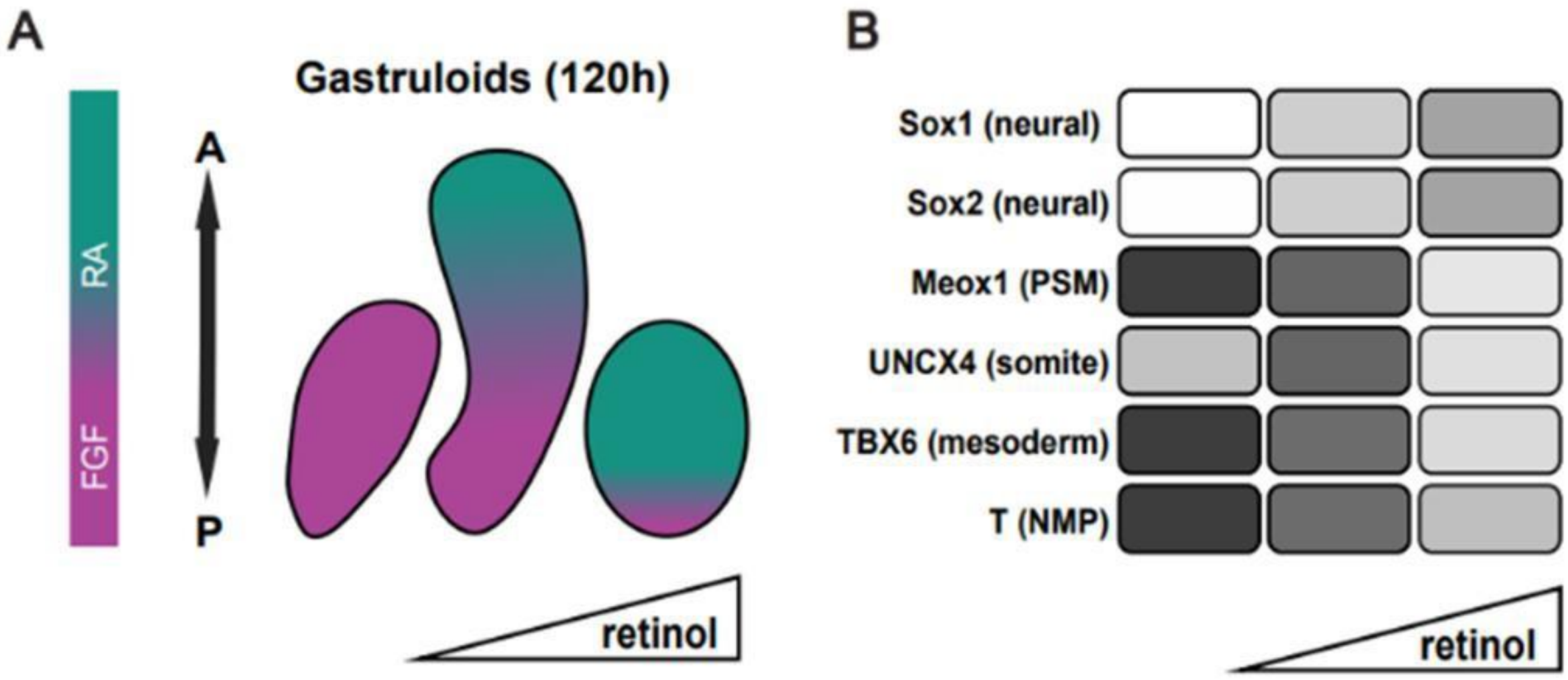

Figure R.2.10. Model for the action of RA on cell specification. (A) Sketch depicting FGF and RA signaling polarization in mouse gastruloids 120 h after aggregation. (B) The effect of retinol on key ectoderm (Sox2, Sox1) and mesoderm (Tbx6, Meox1 and Uncx4.1) markers.

21) Line 452: Formatting: 0,1 decimal comma. In English usually a decimal dot is used
We have corrected the decimal formatting from commas to dots throughout the manuscript, including all figure captions and supplementary data, to adhere to standard English scientific notation.

22) Supplemental Figure 4 & 5: Scale bar info is missing. In general, scale bars are in many cases very difficult to see, likely due to the small image sizes.
We have standardized and enlarged the scale bars across all figures, including Supplemental Figures 4 and 5 (new Suppl. Fig 8,9), to ensure clear visibility. The specific dimensions for each bar are now explicitly stated in the corresponding figure legends.

<u>Second decision letter</u>

MS ID#: dev.205153R1

MS TITLE: Retinoic acid and FGF signaling interact to control elongation and lineage specification in a mouse gastruloid model.

AUTHORS: Mehmet Yildiz; Noëlle Dommann; Tugce Kardelen Hasdemir; Anna Alemany; Pascale Dijkers; Esther De Vries; Niels Geijsen

Dear Dr Geijsen,

© 2026. Published by The Company of Biologists under the terms of the Creative Commons Attribution License (https://creativecommons.org/licenses/by/4.0/).

I am happy to tell you that your manuscript has been accepted for publication in Development, pending our standard publication integrity checks.

Reviewer 3

The authors have thoroughly and sufficiently replied to the previous Reviewers' questions and integrated the necessary corrections. The manuscript in the current form is sound and easy to read.

© 2026. Published by The Company of Biologists under the terms of the Creative Commons Attribution License (https://creativecommons.org/licenses/by/4.0/).
